# HPK1 Dysregulation‐Associated NK Cell Dysfunction and Defective Expansion Promotes Metastatic Melanoma Progression

**DOI:** 10.1002/advs.202400920

**Published:** 2024-06-03

**Authors:** Woo Seon Choi, Hyung‐Joon Kwon, Eunbi Yi, Haeun Lee, Jung Min Kim, Hyo Jin Park, Eun Ji Choi, Myoung Eun Choi, Young Hoon Sung, Chong Hyun Won, Chang Ohk Sung, Hun Sik Kim

**Affiliations:** ^1^ Department of Microbiology Stem Cell Immunomodulation Research Center Asan Medical Center University of Ulsan College of Medicine Seoul 05505 Republic of Korea; ^2^ Department of Dermatology Asan Institute for Life Sciences Asan Medical Center University of Ulsan College of Medicine Seoul 05505 Republic of Korea; ^3^ Department of Cell and Genetic Engineering Asan Medical Center University of Ulsan College of Medicine Seoul 05505 Republic of Korea; ^4^ Department of Pathology Asan Medical Institute of Convergence Science and Technology Asan Medical Center University of Ulsan College of Medicine Seoul 05505 Republic of Korea

**Keywords:** HPK1, Dysfunction, Lung metastasis, Melanoma, Natural killer cell

## Abstract

Distant metastasis, the leading cause of cancer death, is efficiently kept in check by immune surveillance. Studies have uncovered peripheral natural killer (NK) cells as key antimetastatic effectors and their dysregulation during metastasis. However, the molecular mechanism governing NK cell dysfunction links to metastasis remains elusive. Herein, *MAP4K1* encoding HPK1 is aberrantly overexpressed in dysfunctional NK cells in the periphery and the metastatic site. Conditional HPK1 overexpression in NK cells suffices to exacerbate melanoma lung metastasis but not primary tumor growth. Conversely, *MAP4K1*‐deficient mice are resistant to metastasis and further protected by combined immune‐checkpoint inhibitors. Mechanistically, HPK1 restrains NK cell cytotoxicity and expansion via activating receptors. Likewise, HPK1 limits human NK cell activation and associates with melanoma NK cell dysfunction couples to TGF‐β1 and patient response to immune checkpoint therapy. Thus, HPK1 is an intracellular checkpoint controlling NK‐target cell responses, which is dysregulated and hijacked by tumors during metastatic progression.

## Graphical abstract

NK cells are key anti‐metastatic effectors but are dysregulated during metastasis with elusive molecular mechanisms. Using a spontaneous model of metastatic melanoma and clinical samples, HPK1 is dysregulated in peripheral NK cells during metastasis and causally linked to NK cell dysfunction, metastatic progression, and response to immune checkpoint therapy via limiting NK cell cytotoxicity and expansion.

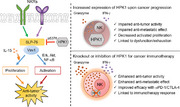

## 1 Introduction

Cancer metastasis is the spread of malignant primary cancer cells to distant tissues and organs for the formation of secondary cancer and accounts for over 90% of cancer‐related mortality. Metastasis is a systemic disease in which systemic factors derived from metastatic cancer can alter host metabolism and immunity locally and systemically to foster metastatic outgrowth.^[^ ^1^, ^2^, ^3^ ^]^ Overt metastasis remains largely incurable due to the dynamic plasticity of metastatic cancer cells, their co‐option of the metastatic niche, and evasion of immune surveillance.^[^ ^1^, ^4^ ^]^ Despite its limited efficacy, the success of immunotherapy in advanced‐stage tumors highlights the importance of tumor‐immune cell interaction in controlling metastasis. Disseminated cancer cells in the circulation and metastatic sites are recognized and eliminated by the antitumor activity of the immune system.^[^ ^2^, ^3^ ^]^ Experimental evidence indicates that few surviving cells escaping immune surveillance (<0.1%) manage to form metastases.^[^ ^5^ ^]^ In these processes, both systemic and local immunity in the tumor microenvironment (TME) are required to mount effective natural and therapeutic antitumor immune responses.^[^ ^2^, ^6^ ^]^ This is supported by the finding of local and systemic immunosuppression prior to metastasis, focusing on adaptive T‐cell responses marked by diminished activation and exhaustion.^[^ ^2^, ^7^, ^8^ ^]^ However, relatively little is known about the contribution of innate immune surveillance beyond T cells to metastasis formation in the metastatic site and the periphery in particular.

Natural killer (NK) cells are key effectors in cancer immunosurveillance with an innate selectivity and capacity to spontaneously eliminate cells undergoing malignant transformation.^[^ ^9^, ^10^ ^]^ NK cells specialize in killing tumor cells with major histocompatibility complex (MHC) class I deficiency that have escaped recognition by CD8 T cells, thus complementing T cells in tumor immune surveillance.^[^ ^11^, ^12^ ^]^ Accumulating evidence suggest that NK cells exert robust control on metastatic dissemination and colonization rather than primary tumor growth.^[^ ^13^, ^14^ ^]^ Supporting this, NK cell deficiency by the antibody‐mediated depletion or genetic targeting of NKp46+ cells leads to marked increases in metastatic colonization but not primary tumor growth in experimental mouse models.^[^ ^15^, ^16^ ^]^ In addition, impaired NK cell cytotoxic activity has been observed in circulation and TME during metastatic progression^[^ ^2^, ^17^, ^18^, ^19^ ^]^ and correlates with metastatic burden in diverse cancer types.^[^ ^20^, ^21^ ^]^ Mechanistic studies have associated the metastatic subversion of NK cell surveillance with altered NK cell phenotypes, characterized by the downregulation of activating receptors or upregulation of inhibitory receptors, along with impaired cytotoxic potential.^[^ ^2^, ^17^, ^19^ ^]^ Moreover, recent studies have revealed functional defects of patient NK cells without cognate receptor alterations in the periphery,^[^ ^18^, ^22^, ^23^ ^]^ suggesting the contribution of distinct mechanisms to NK cell dysfunction beyond phenotypic alteration. However, few studies have been conducted on the cell‐intrinsic molecular mechanism governing NK cell dysfunction that occurs during spontaneous metastasis formation and its causal contribution to metastatic outcomes. It also remains unclear whether this finding can be translated to human as well as mouse models. This study would provide an important insight into tumor‐immune evasion and potential therapeutic targets for metastatic cancer.

Using a spontaneous model of metastatic melanoma, we herein obtained evidence indicating that HPK1 is aberrantly overexpressed in NK cells in the metastatic site and periphery, leading to NK cell dysfunction and metastatic progression. This finding was supported by the targeted overexpression of HPK1 in NK cells and HPK1 deletion using a mouse model of spontaneous and experimental metastasis. We also observed consistent results with clinical samples and public scRNA‐seq data from malignant melanoma patients and propose HPK1 upregulation as an evasion mechanism of NK cell surveillance by metastatic cancer cells and HPK1 as a druggable target for NK cell‐based cancer immunotherapy.

## 2 Results

### 2.1 High HPK1 Expression Correlates with NK Cell Dysfunction During Spontaneous Metastasis Formation

**Figure 1:**
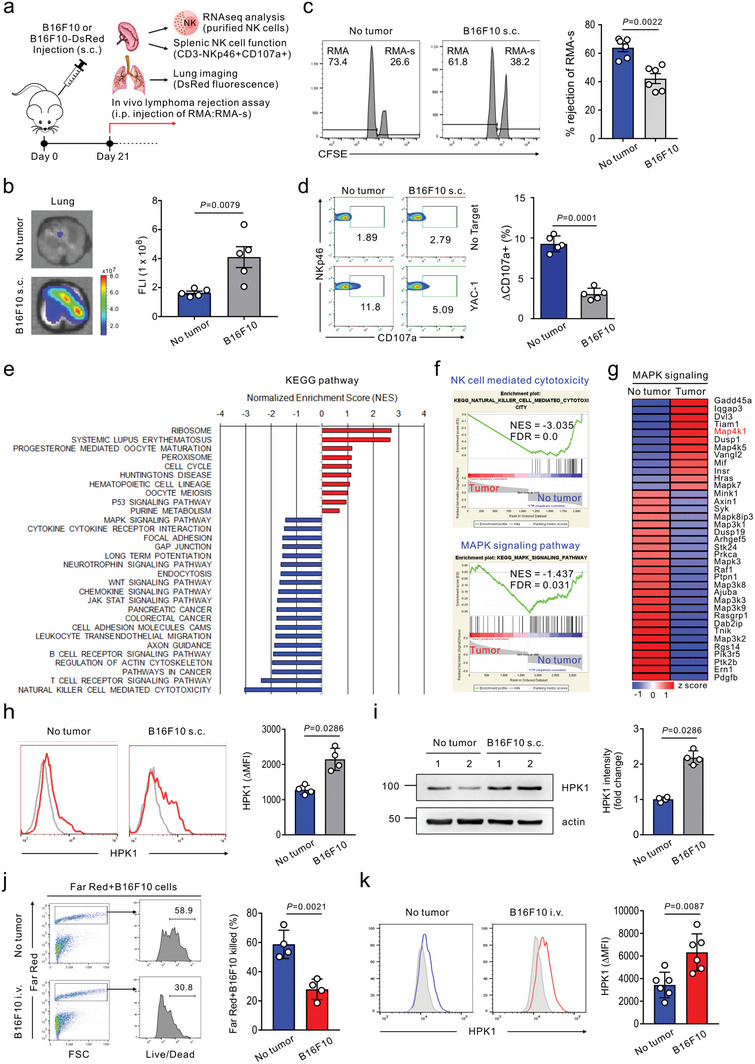
Dysregulated HPK1 expression correlates with peripheral NK cell dysfunction and spontaneous melanoma metastasis. a) Schematic diagram of the experimental design and steps for the in vivo and ex vivo studies 21 days after DsRed‐B16F10 or B16F10 melanoma inoculation. These studies include the imaging of spontaneous lung metastasis, in vivo lymphoma rejection assay, assessment of cytolytic activity of splenic NK cells, and RNA‐seq analysis of purified splenic NK cells. b) Representative lung fluorescence images showing spontaneous lung metastasis (left) and graph of its quantification by fluorescence intensity (FLI) (right) following subcutaneous (s.c.) injection of DsRed‐B16F10 cells (*n* = 5 mice per group). Tumor‐free mice were used as controls. c) NK cell‐mediated lymphoma clearance assay in control tumor‐free or B16F10‐s.c. inoculated mice (*n* = 6 mice per group) showing the representative result (left) and graph (right) of the percent cleared NK‐sensitive RMA‐s cells relative to NK‐resistant RMA cells. d) Degranulation assay of splenic NK cells from control tumor‐free or B16F10‐s.c. inoculated mice (*n* = 5 mice per group) against YAC‐1 cells. Representative result (left) and graph (right) showing the percent increase of CD107a^+^ NK cells after stimulation with YAC‐1 cells relative to CD107a^+^ NK cells without stimulation (ΔCD107a^+^ cells). e) KEGG analysis of genes that are enriched in splenic NK cells from control tumor‐free mice versus B16F10‐s.c. inoculated mice (day 21). Analysis was performed on RNA pooled from 3 individual mice per group. f) GSEA of genes that are upregulated in comparison of NK cell‐mediated cytotoxicity or MAPK signaling pathway between splenic NK cells from control tumor‐free and B16F10‐s.c. inoculated mice (day 21). g) Heatmap of DEGs in comparison of MAPK signaling between splenic NK cells from control tumor‐free and B16F10‐s.c. inoculated mice (day 21). h,i) Flow cytometry (h) and Western blot (i) analysis of HPK1 expression in splenic NK cells from control tumor‐free or B16F10‐s.c. inoculated mice (day 21) (*n* = 4 mice per group). Representative result (left) and graph (right) showing the MFI (h) of HPK1 expression relative to isotype control (ΔMFI) and the intensity (i) of HPK1 expression in NK cells from tumor‐burdened mice relative to NK cells from control tumor‐free mice (fold change). j) Representative flow cytometric analysis of NK cell cytotoxicity against B16F10 cells in the control tumor‐free or metastasis‐bearing lungs of mice (day 21) (*n* = 4 mice per group) (left) and graph showing Live/Dead stain‐positive dead cells (right). Cytotoxicity was measured after in vitro coculture with Far Red‐labeled B16F10 cells for 6 h, followed by staining with Live/Dead Green cell dye. k) Flow cytometric analysis of HPK1 expression in NK cells from control tumor‐free or metastasis‐bearing lungs of mice (day 21) relative to isotype control (ΔMFI) showing the representative result (left) and graph (right) (*n* = 6 mice per group). Data were pooled from 2 independent experiments (b–d and h–k) and are expressed as mean ± SEM (b and c) or mean ± SD (d, h, i, j, and k); each dot represents an individual mouse. Data were analyzed using the Mann‐Whitney U‐test (b, c, h, i, and k) and two‐tailed unpaired t‐test (d and j); actual *P*‐values are indicated.

NK cell cytotoxic activity in the periphery has long been associated with the elimination of disseminated tumor cells and suppression of distant site metastasis.^[^ ^10^, ^13^, ^20^ ^]^ To identify the molecular mechanism underlying systemic NK cell dysfunction linked to metastatic tumor progression, we used a spontaneous metastasis model of B16F10 melanoma cells. B16F10 cells implanted into the skin spontaneously metastasize to the lung and develop detectable metastatic foci 3 weeks after inoculation.^[^ ^24^, ^25^ ^]^ To this end, B16F10 cells expressing DsRed^[^ ^25^ ^]^ were used to facilitate a quantitative assessment of metastatic colonization through fluorescence intensity (**Figure** **1**a). A significant increase in spontaneous metastasis to the lung was observed in mice burdened with melanoma cells after 3 weeks, as measured by fluorescence imaging of pulmonary metastatic growth (Figure 1b). NK cells are key to controlling the systemic metastasis of B16F10 cells attributable to their defect in MHC class I surface expression.^[^ ^25^, ^26^ ^]^ Thus, we studied whether the spontaneous lung metastasis of B16F10 cells correlated with systemic NK cell dysfunction. To assess systemic NK cell cytotoxicity in vivo, we employed a syngeneic lymphoma rejection assay in which MHC class I‐deficient RMA‐s cells but not their parent RMA cells were selectively cleared by NK cells^[^ ^27^ ^]^ (Figure 1a). To facilitate their identification, RMA‐s and RMA cells were labeled with high and low concentrations of carboxyfluorescein succinimidyl ester (CFSE) dye, respectively, and equal numbers of the cells were co‐injected intraperitoneally into mice. We observed a selective clearance of RMA‐s cells relative to RMA cells, which was significantly impaired in tumor‐burdened mice compared with that in control tumor‐free mice (Figure 1c). Consistently, the cytolytic activity of splenic NK cells against YAC‐1 target cells was significantly impaired in mice with metastatic progression of B16F10 cells (Figure 1a,d).

Emerging evidence indicates a discernible alteration of peripheral immune cells that coincides with metastatic tumor progression.^[^ ^1^, ^3^ ^]^ The spleen acts as a reservoir for diverse immune cells including NK cells, and thus contributes to shaping systemic immunity.^[^ ^28^ ^]^ Moreover, many alterations to immune organization in the circulation have been mirrored in the spleen of mouse models.^[^ ^2^ ^]^ Given a distinct dysfunction of splenic NK cells (Figure 1d), we performed bulk RNA sequencing of NK cells purified from the spleen of metastatic tumor‐bearing mice to elucidate the molecular mechanism underlying NK cell dysfunction linked to metastatic progression (Figure 1a). Differentially expressed genes (DEGs) were identified as those upregulated or downregulated by >2‐fold between metastatic tumor‐bearing mice and control mice. Analysis of DEG retrieved Gene Ontology terms that were associated with cell proliferation such as “cell cycle,” “cell division,” or “regulation of cell proliferation” (Figure S1a, Supporting Information). We also noted that some of the terms enriched referred to immune regulation such as “immune system process” or “innate immune response.” The Kyoto Encyclopedia of Genes and Genomes (KEGG) analysis was also conducted and highlighted the downregulation of signaling pathways related to NK cell effector function such as “NK cell‐mediated cytotoxicity,” “T cell receptor signaling pathway,” “JAK‐STAT signaling pathway,” and “MAPK signaling pathway” (Figure 1e). Furthermore, gene set enrichment analysis (GSEA) using RNA‐seq data confirmed a significant decrease in the gene sets of NK cell‐mediated cytotoxicity and MAPK signaling pathway in the NK cells of metastatic tumor‐bearing mice (Figure 1f; Figure S1b, Supporting Information). MAPK ERK and JNK are key signaling molecules linked to the cytotoxic degranulation of NK cells and are commonly triggered by diverse activating receptors with distinct proximal signaling modules, either alone or in combination.^[^ ^12^, ^29^ ^]^ Thus, we focused on genes related to MAPK signaling pathway downregulation. Among potential target candidates, *Gadd45a*, a growth arrest, and DNA damage‐inducible gene implicated in the negative regulation of T cell proliferation^[^ ^30^ ^]^ and p38 MAPK activation,^[^ ^31^ ^]^ as well as *MAP4K1* encoding HPK1, a negative regulator of T cell receptor signaling and MAPK ERK activation,^[^ ^32^, ^33^ ^]^ were highly expressed in dysfunctional NK cells (Figure 1g).

Given HPK1 as a mediator of T cell dysfunction and an attractive immuno‐oncology drug target,^[^ ^34^, ^35^ ^]^ we further investigated the role of aberrant HPK upregulation in NK cell dysfunction and metastatic progression. First, we assessed whether the heightened mRNA expression level of HPK1 could occur at the protein level. A flow cytometry‐based approach was developed to quantify the relative protein levels of HPK1 using a specific antibody for HPK1. Together with Western blot analysis, proper functioning of the measurement was demonstrated by the diminished expression of HPK1 by siRNA‐mediated knockdown and its absence in K562 cells that do not express HPK1^[^ ^36^ ^]^ (Figure S2, Supporting Information). Compared with the expression levels in the tumor‐free control, metastatic progression of B16F10 cells led to HPK1 upregulation in splenic NK cells at the protein level, as determined by flow cytometry (Figure 1h) and Western blot analysis (Figure 1i). To determine whether HPK1 upregulation is linked to metastatic progression, poorly metastatic B16F1 cells instead of highly metastatic B16F10 cells were implanted in the skin of the mice. There was no significant alteration in the cytotoxicity of splenic NK cells against B16F10 or YAC‐1 target cells and the level of HPK1 expression in the NK cells 3 weeks after inoculation (Figure S3, Supporting Information), suggesting HPK1 upregulation probably through a systemic effect of metastatic progression. We next investigated whether NK cell expression of HPK1 is also affected in the metastatic site using an experimental model of metastatic melanoma. B16F10 cells administered via the tail vein primarily develop pulmonary metastatic foci within 2 weeks.^[^ ^37^ ^]^ We found an attenuated cytotoxicity of NK cells against B16F10 cells in the metastasis‐bearing lungs of mice (Figure 1j), which correlated with an aberrant upregulation of HPK1 in NK cells (Figure 1k). Thus, HPK1 upregulation, NK cell dysfunction, and metastatic progression may be linked in the metastatic site and periphery.

### 2.2 HPK1 Overexpression in NK Cells Promotes NK Cell Dysfunction and Metastatic Progression

We next studied the direct role of aberrant HPK1 upregulation in metastasis formation along with NK cell function using transgenic (Tg) mice with NK cell‐specific HPK1 overexpression (Figure S4, Supporting Information). As previously described,^[^ ^38^, ^39^ ^]^ we constructed Tg mouse lines harboring the loxP‐STOP‐loxP‐mouse *MAP4K1* transgene. In this conditional transgene, the expression of a full‐length *MAP4K1* was driven by a strong *CAG* promoter comprising the CMV immediate‐early enhancer and the chicken β‐actin promoter, but its transcription is blocked by the triple polyadenylation signals (3×PolyA) flanked with loxP sequences (Figure S4a, Supporting Information). After removing the loxP‐flanked 3×PolyA by Cre recombinase in the transfected cells, HPK1 protein was efficiently induced from the supercoiled construct and linearized transgene (Figure S4b,c, Supporting Information). Using the linearized transgene construct, we established and characterized 2 positive Tg mouse lines (*MAP4K1* ^Tg^ and *MAP4K1* ^Tg7^) with differential expression levels of the transgene (high vs moderate level) (Figure S4d, Supporting Information). To conditionally express *MAP4K1* transgene in NK cells, *MAP4K1* ^Tg^ mice were bred with mice expressing improved Cre (iCre) recombinase under the control of *Ncr1* (encoding NKp46) promoter^[^ ^40^ ^]^ to obtain *Ncr1* ^iCre^ *MAP4K1* ^Tg^ mice heterozygous at both loci (Figure S4d, Supporting Information). Similar to *Ncr1* ^iCre^ mice, *MAP4K1* ^Tg^ mice and *Ncr1* ^iCre^ *MAP4K1* ^Tg^ mice were obtained at Mendelian frequencies, exhibited no developmental abnormalities, and had normal reproductive ability. As expected, HPK1 expression was highly upregulated in CD3^−^NK1.1^+^ NK cells from *Ncr1* ^iCre^ *MAP4K1* ^Tg^ mice (≈4‐fold increase) but moderately in NK cells from *Ncr1* ^iCre^ *MAP4K1* ^Tg7^ mice (≈2‐fold increase) (Figure S4f,h, Supporting Information). Moreover, the percentage of NK cells significantly decreased and inversely correlated with the HPK1 expression level (Figure S4e,g, Supporting Information), suggesting that HPK1 may affect NK cell proliferation.

**Figure 2:**
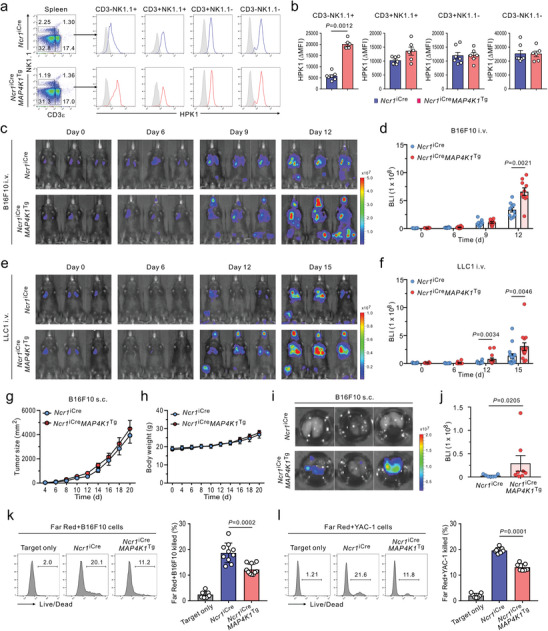
Conditional HPK1 overexpression in NK cells exacerbates spontaneous and experimental melanoma metastasis by driving NK cell dysfunction. a,b) Representative (a) and quantitative (b) flow cytometric analysis of HPK1 expression in lymphocytes, graphed on CD3ε by NK1.1 dot plots, among CD45^+^ cell populations in the spleen of *Ncr1* ^iCre^ (*n* = 7 mice) versus *Ncr1* ^iCre^ *MAP4K1* ^Tg^ (*n* = 6 mice) mice. Selective overexpression of HPK1 in CD3^−^NK1.1^+^ NK cells is shown in *Ncr1* ^iCre^ *MAP4K1* ^Tg^ mice compared with *Ncr1* ^iCre^ mice. c,d) Representative images (c) and quantification (d) of metastasis to multiple organs including the lung in *Ncr1* ^iCre^ or *Ncr1* ^iCre^ *MAP4K1* ^Tg^ mice (*n* = 10 mice per group) after administration of B16F10‐Luc2 cells by luciferase‐based bioluminescence imaging (BLI). (e,f) Representative images e) and quantification f) of metastasis in *Ncr1* ^iCre^ (*n* = 16 mice) or *Ncr1* ^iCre^ *MAP4K1* ^Tg^ (*n* = 14 mice) mice following administration of LLC1‐Luc2 cells by BLI. g–j) B16F10‐Luc2 cells were injected subcutaneously into *Ncr1* ^iCre^ (*n* = 7 mice) or *Ncr1* ^iCre^ *MAP4K1* ^Tg^ mice (*n* = 8 mice). Shown are the tumor sizes g), body weights (h), representative lung bioluminescence images showing spontaneous lung metastases (i), and quantification of lung metastases by BLI (j) on day 20. k,l) Flow cytometric analysis of NK cell cytotoxicity against B16F10 cells (k; *n* = 10 mice per group) and YAC‐1 cells (l; *n* = 7 mice per group) in the spleen of *Ncr1* ^iCre^ or *Ncr1* ^iCre^ *MAP4K1* ^Tg^ mice showing the representative result (left) and graph (right) of Live/Dead stain‐positive dead cells. Data were pooled from 2 or 3 independent experiments and are expressed as mean ± SEM (b, d, f, g, h, and j) or mean ± SD (k and l); each dot represents an individual mouse. Data were analyzed using the Mann‐Whitney U‐test (b, d, f, j) and 2‐tailed unpaired t‐test (k, l); actual *p*‐values are indicated.

Having established Tg mice expressing differential HPK1 levels in NK cells, we next studied the effects of HPK1 overexpression in NK cells on pulmonary metastasis. Among different lymphocyte populations, we confirmed a selective overexpression of HPK1 in CD3^−^NK1.1^+^ splenic NK cells from *Ncr1* ^iCre^ *MAP4K1* ^Tg^ mice (**Figure** **2**a,b). We then challenged *Ncr1* ^iCre^ and *Ncr1* ^iCre^ *MAP4K1* ^Tg^ mice with B16F10 cells that preferentially metastasize to the lung upon intravenous injection, followed by the analysis of pulmonary metastatic growth. *Ncr1* ^iCre^ *MAP4K1* ^Tg^ mice exhibited significantly more severe lung metastasis compared to that in *Ncr1* ^iCre^ mice, as determined by whole‐body bioluminescent imaging (Figure 2c,d). In particular, *Ncr1* ^iCre^ *MAP4K1* ^Tg^ mice were also incapable of clearing metastasis to other organs including the brain as efficiently as *Ncr1* ^iCre^ mice, which was detectable on day 6 and evident around day 9. Consistent with this finding, we observed a similar aggravation of metastasis to the lung and other organs by HPK1 overexpression in NK cells using an experimental metastasis model of NK cell‐sensitive LLC1 lung cancer cells^[^ ^16^ ^]^ (Figure 2e,f). Given the dependence of NK cell activity on MHC class I expression, we investigated the effect of HPK1 overexpression in NK cells on pulmonary metastasis of TC‐1 lung cancer cells that express a high level of MHC class I surface expression (Figure S5a, Supporting Information) and are resistant to NK cell‐mediated cytolysis.^[^ ^41^, ^42^ ^]^ The TC‐1‐derived metastatic load in *Ncr1* ^iCre^ *MAP4K1* ^Tg^ mice was comparable to that in *Ncr1* ^iCre^ mice (Figure S5b,c, Supporting Information), confirming the selective effect of HPK1 overexpression in NK cells on metastasis control of NK‐sensitive tumor cells. We next assessed whether NK cell expression of HPK1 also affects the development of primary tumors at the orthotopic site. B16F10 cells were implanted subcutaneously in *Ncr1* ^iCre^ and *Ncr1* ^iCre^ *MAP4K1* ^Tg^ mice. The growth of primary tumors and body weight over 3 weeks did not differ significantly between these 2 groups (Figure 2g,h). However, spontaneous metastasis to the lung increased significantly 3 weeks following B16F10 melanoma injection in *Ncr1* ^iCre^ *MAP4K1* ^Tg^ mice compared to that in *Ncr1* ^iCre^ mice (Figure 2i,j). This finding indicated that HPK1 overexpression in NK cells suffices to aggravate metastatic progression rather than primary tumor growth, corroborating a key role of NK cells in preventing metastatic dissemination but not primary tumor growth in NK cell‐deficient experimental mouse models.^[^ ^13^, ^15^, ^16^ ^]^ This finding was also supported by the principal control of subcutaneous growth of B16F10 cells by T cells but not NK cells.^[^ ^34^ ^]^ Consistently, NK cell overexpression of HPK1 correlated with the reduced cytotoxicity of splenic NK cells against B16F10 (Figure 2k) or YAC‐1 (Figure 2l) target cells. Thus, impaired NK cell‐mediated clearance of metastatic cancer cells may be a mechanism of aggravated metastatic burden by HPK1 overexpression in NK cells.

Given an inverse correlation between the HPK1 expression level and the NK cell frequency (Figure S4, Supporting Information), metastatic progression may depend on the level of HPK1 expression in NK cells. We assessed the metastatic susceptibility of other *Ncr1* ^iCre^ *MAP4K1* ^Tg7^ mice that express moderate levels (≈2‐fold increase) of HPK1 in NK cells compared to the susceptibility of mice with high levels (≈4‐fold increase) of HPK1 in *Ncr1* ^iCre^ *MAP4K1* ^Tg^ NK cells. We observed a significant but to a lesser extent increase in metastatic burden in the lung and other organs of *Ncr1* ^iCre^ *MAP4K1* ^Tg7^ mice (ΔBLI: ≈2.7 × 10^8^) compared to that in *Ncr1* ^iCre^ *MAP4K1* ^Tg^ mice (ΔBLI: ≈3.2 × 10^8^) following tail vein administration of B16F10 cells (Figure S6a,b, Supporting Information). Moreover, the cytotoxicity of splenic NK cells against B16F10 or YAC‐1 target cells was less significantly impaired by the moderate level of HPK1 overexpression (Δtarget cells killed: ≈3.7% or ≈3.4%; Figure S6c,d, Supporting Information versus ≈6.4% or ≈6.5%; Figure 2k,l). Thus, the HPK1 expression level may correspond to the degree of NK cell dysfunction in terms of cytotoxicity and consequent metastatic susceptibility.

### 2.3 NK Cell Overexpression of HPK1 Controls NK Cell Proliferation and Cytotoxic Capacity Upon Target Cell Recognition

The aggravated metastatic susceptibility of *Ncr1* ^iCre^ *MAP4K1* ^Tg^ mice (Figure 2) may be attributed to defects in NK cell proliferation and cytotoxic function, given a reduced frequency of NK cells (CD3^−^NK1.1^+^) due to HPK1 overexpression (Figure S4, Supporting Information). Supporting this, the proportion of mature NK cells, defined by CD49b (DX5, integrin VLA‐2α) along with NK1.1,^[^ ^43^ ^]^ also significantly decreased in the spleen of *Ncr1* ^iCre^ *MAP4K1* ^Tg^ mice relative to *Ncr1* ^iCre^ mice (Figure S7a,b, Supporting Information). In comparison, the proportion of splenic NK cells (CD3^−^NK1.1^+^, NK1.1^+^CD49b^+^) in *MAP4K1* ^Tg^ mice without Cre recombinase expression was comparable to that in wild‐type (WT) control mice (Figure S7c,d, Supporting Information). Thus, the decreased frequency of NK cells in *Ncr1* ^iCre^ *MAP4K1* ^Tg^ mice may be causally linked to HPK1 overexpression but not to the presence of transgenes. Consistent with the level of HPK1 expression, we observed less severe defects in the proportion of splenic NK cells (CD3^−^NK1.1^+^, NK1.1^+^CD49b^+^) in *Ncr1* ^iCre^ *MAP4K1* ^Tg7^ mice (Figure S7e,f, Supporting Information) relative to *Ncr1* ^iCre^ *MAP4K1* ^Tg^ mice (Figure S7a,b, Supporting Information). Moreover, to confirm the effect of HPK1 overexpression on the NK cell population, flow cytometry was used to analyze different leukocyte populations in peripheral blood and various organs of *Ncr1* ^iCre^ *MAP4K1* ^Tg^ mice (Figure S8, Supporting Information). HPK1 overexpression did not affect the proportion of CD11b^+^Ly6G^+^Ly6C^int^ neutrophils, CD11b^+^Ly6G^−^Ly6C^+^ monocytes, CD11c^+^F4/80^−^MHCII^+^ dendritic cells, or CD11b^+^F4/80^+^MHCII^+^ M1‐like and CD11b^+^F4/80^+^CD206^+^ M2‐like macrophages among the CD45^+^ cell populations (Figure S9a,b, Supporting Information). By contrast, CD3^−^NK1.1^+^ NK cells, but not other lymphocytes including γδ T cells, CD8^+^ T cells, and CD4^+^ T cells, were specifically and significantly downregulated by HPK1 overexpression in the spleen, lung, bone marrow (BM), and blood of *Ncr1* ^iCre^ *MAP4K1* ^Tg^ mice (Figure S9, Supporting Information). Murine NK cells acquire effector function via a three‐stage maturation program, as defined by differential surface expression of CD11b and CD27 (CD11b^−^CD27^+^, CD11b^+^CD27^+^, and fully mature CD11b^+^CD27^−^).^[^ ^44^ ^]^ We observed comparable frequencies of these subsets with comparable levels of diverse surface markers (i.e., KLRG1, NKG2A/C/E, NKG2D, CD122, or CD226) in splenic NK cells (Figure S10, Supporting Information); thus, HPK1 overexpression does not seem critical for the functional maturation and receptor expression of NK cells.

**Figure 3:**
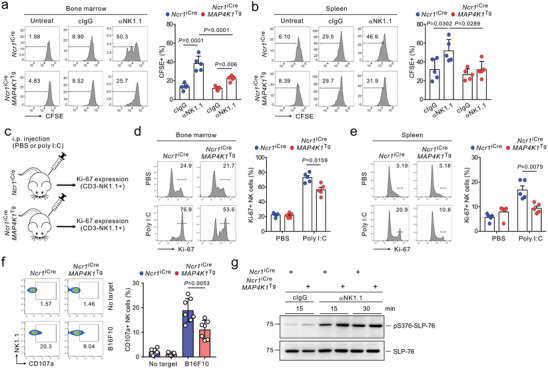
Conditional HPK1 overexpression restrains NK cell cytotoxicity and expansion via activating receptors. a,b) Purified BM NK cells (a) and splenic NK cells (b) from *Ncr1* ^iCre^ or *Ncr1* ^iCre^ *MAP4K1* ^Tg^ mice (*n* = 5 mice per group) were labeled with CFSE and stimulated with plate‐immobilized anti‐NK1.1 Ab in the presence of IL‐15 for 3 days. NK cell proliferation was assessed by CFSE dilution using flow cytometry. Shown is the representative result (left) and graph (right) of the percent increase of CFSE‐diluted proliferating NK cells. c) Schematic diagram showing the analysis of in vivo NK cell proliferation 30 h after poly I:C treatment. d,e) Flow cytometric analysis of in vivo proliferation of BM NK cells (d) and splenic NK cells (e) from *Ncr1* ^iCre^ or *Ncr1* ^iCre^ *MAP4K1* ^Tg^ mice (*n* = 5 mice per group) following poly I:C treatment, as assessed by Ki‐67 expression in CD3^−^NK1.1^+^ NK cells. Shown is the representative result (left) and graph (right) of the percent increase of Ki‐67‐positive NK cells. f) Degranulation assay of splenic NK cells against B16F10 cells (*n* = 7 mice per group). Representative result (left) and graph (right) showing percent increase of CD107a^+^ NK cells. g) Purified NK cells from the spleen of *Ncr1* ^iCre^ or *Ncr1* ^iCre^ *MAP4K1* ^Tg^ mice were stimulated with control IgG or anti‐NK1.1 Ab for the indicated times. Cell lysates were immunoblotted for phospho‐SLP‐76 at serine 376 (pS376) or total SLP‐76. Data are representative of 3 independent experiments. Data were pooled from 2 independent experiments (a‐f) and represent the mean ± SD (a, b, and f) or mean ± SEM (d and e); each dot represents an individual mouse. Data were analyzed using a 2‐tailed unpaired t‐test (a, b, and f) and Mann‐Whitney U‐test (d and e); actual *P*‐values are indicated.

Next, we directly tested the potential effect of HPK1 overexpression on NK cell proliferation. NK cell proliferation is driven by IL‐2 family cytokines, particularly IL‐15, and augmented by signaling through activating receptors that recognize target cells.^[^ ^45^ ^]^ We isolated NK cells from the BM or spleen of *Ncr1* ^iCre^ and *Ncr1* ^iCre^ *MAP4K1* ^Tg^ mice and cultured CFSE‐labeled NK cells in vitro for 3 days upon stimulation with NK1.1 in the presence of IL‐15. Compared with a robust proliferation of *Ncr1* ^iCre^ NK cells, *Ncr1* ^iCre^ *MAP4K1* ^Tg^ NK cell proliferation was significantly impaired, as measured by CFSE dilution (**Figure** **3**a,b). However, the in vitro proliferation did not differ between *Ncr1* ^iCre^ and *Ncr1* ^iCre^ *MAP4K1* ^Tg^ NK cells in response to different IL‐15 concentrations (Figure S11, Supporting Information). Thus, HPK1 overexpression may restrain NK cell proliferation through a significant effect on the stimulation through activating receptors but not IL‐15. To validate this in vivo, *Ncr1* ^iCre^ and *Ncr1* ^iCre^ *MAP4K1* ^Tg^ mice were injected with poly I:C, a TLR3 agonist that can induce NK cell activation and proliferation via cell‐cell contact (e.g., interaction with dendritic cells) and cytokines including IL‐15^[^ ^46^ ^]^ (Figure 3c). We found a vigorous proliferation of NK cells in the BM and spleen of *Ncr1* ^iCre^ mice activated with poly I:C, as measured by a widely used Ki‐67 proliferation marker (Figure 3d,e). By comparison, NK cell proliferation was significantly downregulated in *Ncr1* ^iCre^ *MAP4K1* ^Tg^ mice, indicating that HPK1‐mediated NK cell proliferation also occurs in vivo and may partly account for the reduced frequency of NK cells in *Ncr1* ^iCre^ *MAP4K1* ^Tg^ mice.

We next determined whether HPK1 overexpression is linked to an intrinsic defect in NK cell effector function. The cytolytic activity of NK cells was assessed on a per‐cell basis by flow cytometry‐based analysis of CD107a expression on NK cells. The proportion of activated NK cells responding to B16F10 cells was significantly lower in *Ncr1* ^iCre^ *MAP4K1* ^Tg^ NK cells than in *Ncr1* ^iCre^ NK cells (Figure 3f). Consistently, the cytolytic activity of NK cells was less impaired in *Ncr1* ^iCre^ *MAP4K1* ^Tg7^ mice (ΔCD107a^+^ NK cells: ≈5.28%; Figure S7g, Supporting Information) relative to *Ncr1* ^iCre^ *MAP4K1* ^Tg^ mice (ΔCD107a^+^ NK cells: ≈7.81%; Figure 3f). In T cells, HPK1 negatively regulates T cell receptor‐mediated signaling and activation by inducing the phosphorylation of SLP‐76 at serine residue 376 (pS376).^[^ ^47^ ^]^ NK1.1 activation induced the phosphorylation of SLP‐76 at pS376 in *Ncr1* ^iCre^ NK cells, which was markedly augmented by HPK1 overexpression (Figure 3g). Therefore, the combined defects in proliferation and cytolytic capacity of activated NK cells by HPK1 overexpression may contribute to nullifying the NK cell‐mediated surveillance of metastatic cancer cells.

### 2.4 HPK1 and its Kinase Activity Mediate Human NK Cell Dysfunction

**Figure 4:**
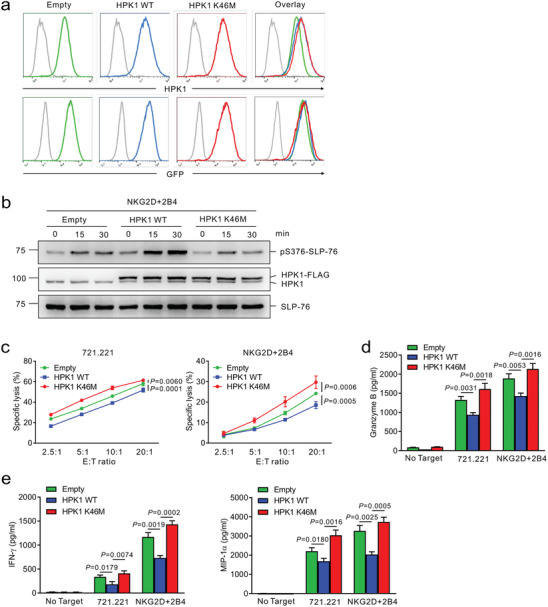
HPK1 overexpression and its kinase activity mediate human NK cell dysfunction. a) Establishment of NKL cell line expressing HPK wild‐type (WT) and its kinase‐dead mutant (K46M) by retroviral transduction. NKL cells transduced with the retrovirus containing sequences for WT or K46M form of HPK1 were sorted for a matched level of GFP expression. The level of HPK1 protein as reflected by GFP expression in the transduced cells was measured by flow cytometry. Representative flow cytometric analysis of HPK1 expression (top) and GFP expression (bottom) in NKL cells transduced with empty vector (green), HPK1 WT (blue), or HPK1 K46M (red). The retroviral construct without an HPK1 sequence (empty) was used as a control. b) NKL cells expressing HPK1 WT or HPK1 K46M were stimulated through a combination of NKG2D and 2B4 for the indicated times. Cell lysates were immunoblotted for phospho‐SLP‐76 at serine 376 (pS376), HPK1, or total SLP‐76. c) Lysis of 721.221 (left) or P815 cells engaging NKG2D and 2B4 (right) by NKL cells expressing HPK1 WT or HPK1 K46M at the indicated effector to target (E:T) cell ratio, as determined by europium assay (triplicate samples per group). d) NKL cells expressing HPK1 WT or HPK1 K46M were stimulated with 721.221 cells or a combination of NKG2D and 2B4 for 2 h. The secretion of granzyme B in the supernatant was measured by ELISA (triplicate samples per group). e) NKL cells expressing HPK1 WT or HPK1 K46M were stimulated with 721.221 cells or a combination of NKG2D and 2B4. After 8 h of incubation, IFN‐γ (left) and MIP‐1α (right) released in the supernatant were measured by ELISA (triplicate samples per group). Data represent the mean ± SD (c, d, and e) and were analyzed using two‐way ANOVA with Dunnett's multiple comparison test (c) and two‐tailed unpaired t‐test (d and e); actual *p*‐values are indicated. All data are representative of at least 3 independent experiments.

An essential question was whether HPK1‐mediated dysfunction of NK cells also occurred during the activation of human NK cells. Besides phosphorylating critical adaptors such as SLP‐76, HPK1 also regulates T‐cell signaling via scaffold‐dependent function independently of its kinase activity.^[^ ^35^, ^48^, ^49^ ^]^ To address these issues, human NK cell line NKL was transduced with retroviruses that express HPK1 WT or its kinase‐dead point mutant form (K46M). Due to the coexpression of green fluorescent protein (GFP) with the cloned HPK1 in the transduced cells, cells were sorted for comparable expression of GFP (**Figure** **4**a). The HPK1 protein levels in the transduced NKL cells were similar between HPK1 WT and HPK1 K46M, as measured by flow cytometry. Overexpression of HPK1 WT enhanced, whereas HPK1 K46M suppressed the phosphorylation of SLP‐76 at serine 376 in response to combined stimulation with NKG2D and 2B4 receptor that complements to induce SLP‐76 phosphorylation at N‐terminal tyrosines required for proper activation of NK cells^[^ ^50^, ^51^ ^]^ (Figure 4b). We then assessed their effects on the functions of NK cells. Cytotoxicity was suppressed by HPK1 WT overexpression but was increased by overexpression of HPK1 K46M mutant upon stimulation with 721.221 target cells and a combination of NKG2D and 2B4, correlating with the cytotoxic release of granzyme B (Figure 4c,d). Likewise, the production of cytokine IFN‐γ and chemokine MIP‐1α was significantly decreased by HPK1 WT overexpression but was enhanced by HPK1 K46M overexpression (Figure 4e). Thus, the effector functions of NK cells could be dysregulated by the level of HPK1 expression in human NK cells, an observation compatible with the results using mice with NK cell‐specific HPK1 overexpression. Moreover, our results suggest HPK1 inactivation by targeting kinase activity as a therapeutic measures to enhance human NK cell activation.

### 2.5 HPK1 Deficiency Confers Resistance to Melanoma Metastasis by Potentiating NK Cells

Having observed HPK1‐mediated NK cell dysfunction in human and mouse NK cells, we tested the effect of HPK1 deficiency on melanoma lung metastasis. We generated a *MAP4K1*‐deficient mouse model by using the CRISPR‐Cas9‐mediated knockout (KO) system (Figure S12a,b, Supporting Information). We established 2 mouse lines with insertion and deletion (indel) mutations (*MAP4K1* KO #1 and *MAP4K1* KO #4) and confirmed the disruption of *MAP4K1* by Sanger sequencing (Figure S12c,d, Supporting Information). For further study, we used and characterized *MAP4K1* KO #1 mouse line (hereafter denoted as *MAP4K1* KO). Phenotypic analysis of peripheral NK cells (CD3^−^NK1.1^+^, NK1.1^+^CD49b^+^) revealed a comparable but slightly increased frequency of NK cells in *MAP4K1* KO mice (Figure S12e,f, Supporting Information). HPK1 expression was abrogated in NK cells of *MAP4K1* KO mice at the protein levels, as measured by flow cytometry (Figure S12g, Supporting Information) and Western blot analysis (Figure S12h, Supporting Information). Consistent with previous studies using *MAP4K1*–deficient mice,^[^ ^33^, ^52^ ^]^ we observed comparable frequencies of other immune cell populations (CD3^+^NK1.1^+^, CD3^+^NK1.1^−^, CD3^−^NK1.1^−^) as well as NK cells (Figure S12i, Supporting Information) and confirmed HPK1 deficiency (Figure S12j, Supporting Information) in *MAP4K1* KO mice.

**Figure 5:**
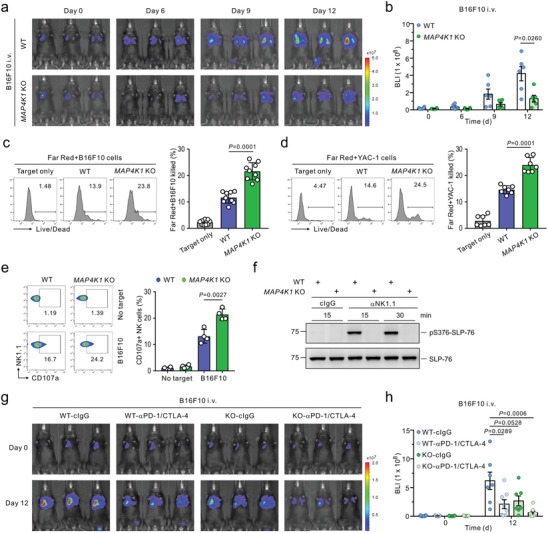
HPK1 deficiency enhances the anti‐metastatic effect of NK cells and the efficacy of checkpoint immunotherapy. a,b) Representative images (a) and quantification (b) of pulmonary metastasis in WT or *MAP4K1* KO mice (*n* = 6 mice per group) following intravenous (i.v.) administration of B16F10‐Luc2 cells by BLI. c,d) Flow cytometric analysis of NK cell cytotoxicity against B16F10 cells (c; *n* = 9 mice per group) and YAC‐1 cells (d; *n* = 7 mice per group) in the spleen of WT or *MAP4K1* KO mice showing the representative result (left) and graph (right) of Live/Dead stain‐positive dead cells. e) Degranulation assay of splenic NK cells against B16F10 cells (*n* = 4 mice per group). Representative result (left) and graph (right) showing percent increase of CD107a^+^ NK cells. (f) Purified splenic NK cells from WT or *MAP4K1* KO mice were stimulated with control IgG or anti‐NK1.1 Ab for the indicated times. Cell lysates were immunoblotted for phospho‐SLP‐76 at serine 376 (pS376) or total SLP‐76. Data are representative of 3 independent experiments. g,h) WT or *MAP4K1* KO mice were i.v. injected with B16F10‐Luc2 cells and treated with either control IgG (500 µg) or anti‐PD‐1 and CTLA‐4 (250 µg each) on days 0 and 3 postinjection, respectively (*n* = 7,8 mice per group). Shown are the representative images (g) and quantification (h) of lung metastasis by BLI (day 12). Data were pooled from 2 or 3 independent experiments (a‐e, g, and h) and are expressed as mean ± SEM (b and h) or mean ± SD (c, d, and e); each dot represents an individual mouse. Data were analyzed using the Mann‐Whitney U‐test (b, h) and two‐tailed unpaired t‐test (c‐e); actual *p*‐values are indicated.

To assess the impact of HPK1 deficiency on melanoma metastasis, we compared the metastatic susceptibility of *MAP4K1* KO and WT control mice after intravenous injection of B16F10 cells, of which host control is primarily mediated by NK cells.^[^ ^16^, ^25^ ^]^ *MAP4K1* KO mice were significantly and highly resistant to experimental metastasis than WT control mice in the lung and other organs on day 12 when challenged with B16F10 cells, as opposed to *Ncr1* ^iCre^ *MAP4K1* ^Tg^ mice (**Figure** **5**a,b). This efficient control of melanoma metastasis correlated with the enhanced cytotoxicity of splenic NK cells against B16F10 or YAC‐1 target cells by HPK1 deficiency (Figure 5c,d). Supporting this, HPK1 deficiency led to the potentiation of cytolytic activity of NK cells on a per‐cell basis (Figure 5e) and the loss of inhibitory phosphorylation of SLP‐76 at serine 376 (Figure 5f). Moreover, HPK1 deficiency significantly attenuated lung metastasis of B16F10 cells that initially colonize in the lung when evaluated at 24 h after inoculation (Figure S13a, Supporting Information), while the frequencies of CD3^−^NK1.1^+^ NK cells and other lymphocytes remained unchanged (Figure S13b,c, Supporting Information). Thus, HPK1 deficiency may impact the cytolytic activity rather than the migration and infiltration of NK cells into metastasis‐bearing lungs despite a requirement for further mechanistic study.

Combined anti‐PD‐1 and anti‐CTLA‐4 checkpoint blockade are currently among the most effective immunotherapy against various cancer types including advanced metastatic melanoma.^[^ ^53^ ^]^ To compare this immune checkpoint therapy (ICT) with HPK1 deficiency, WT control, and *MAP4K1* KO mice were injected intravenously with B16F10 cells and then treated with a control IgG or a combination of anti‐PD‐1 and anti‐CTLA‐4. Metastatic melanoma burden in the lungs was significantly reduced by anti‐PD‐1 and anti‐CTLA‐4 treatment, which was comparable to the protection afforded by HPK1 deficiency alone (Figure 5g,h). Of note, *MAP4K1* KO mice treated with this dual checkpoint blockade were profoundly protected against the development of lung metastasis. Consistent with the notion of anti‐PD‐1 and anti‐CTLA‐4 as established T cell checkpoint inhibitors, this dual checkpoint blockade exhibited therapeutic efficacy in the absence of NK cells, as demonstrated by a significant decrease in lung metastasis of B16F10 cells following dual checkpoint blockade in the mice depleted of NK cells (Figure S14, Supporting Information). Collectively, these results highlighted HPK1 in NK cells as a candidate target for treating metastatic cancer and its cooperative therapeutic combination with anti‐PD‐1 and anti‐CTLA‐4 therapy.

To probe the therapeutic effect of HPK1 deficiency on human NK cells, we silenced the HPK1 expression using siRNA‐mediated knockdown in NKL cells (Figure S15a, Supporting Information). HPK1 knockdown caused a noticeable enhancement of Ca^2+^ mobilization (Figure S15b, Supporting Information) and phosphorylation of Vav1 at tyrosine 174, NF‐κB p65 subunit at serine 536, AKT, and ERK (Figure S15c, Supporting Information), which are required for cytotoxic degranulation and cytokine production.^[^ ^50^, ^51^, ^54^ ^]^ These changes in the phosphorylation of key signaling molecules correlated with the abrogation of inhibitory phosphorylation of SLP‐76 at serine 376 (Figure S15d, Supporting Information). Accordingly, cytotoxicity was markedly augmented by HPK1 depletion in response to NKG2D, 2B4, or both (Figure S15e, Supporting Information). HPK1 knockdown also significantly enhanced the secretion of IFN‐γ and MIP‐1α via NKG2D and 2B4 stimulation (Figure S15f, Supporting Information). Thus, HPK1 could negatively regulate the function of human NK cells through different activating receptors and may function as a checkpoint protein for NK cell activation.

### 2.6 HPK1 Upregulation Correlates with NK Cell Dysfunction in Melanoma Patients (MP)

**Figure 6:**
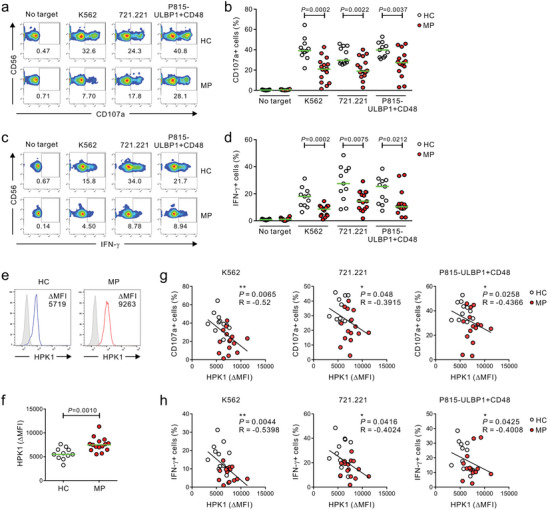
Dysregulated HPK1 upregulation correlates with peripheral NK cell dysfunction in melanoma patients. PBMCs from the healthy control (HC) group (*n* = 11) and melanoma patients (MP) group (*n* = 16) were incubated with conventional K562 or 721.221 target cells, or P815‐ULBP1+CD48 cells that activate NK cells via NKG2D and 2B4. a,b) Cytotoxic degranulation of NK cells showing the representative result (a) and graph (b), as measured by the percent increase of surface expression of CD107a. c,d) Cytokine production by NK cells showing the representative result (c) and graph (d), as measured by percent increase of intracellular expression of IFN‐γ. e,f) Flow cytometric analysis of HPK1 expression in NK cells from HC group (*n* = 11) and MP group (*n* = 15) relative to isotype control (ΔMFI) showing the representative result (e) and graph (f). g,h) The expression of HPK1 (ΔMFI) in NK cells correlates inversely with the percentages of CD107a‐ (g) or IFN‐γ‐positive NK cells (h) after stimulation with all 3 target cells. Data were pooled from 5 independent experiments. Horizontal bars (green) indicate the medians (b, d, and f); each dot represents an individual donor. Data were analyzed using the Mann‐Whitney U‐test (b, d, and f) and Spearman correlation test (g, h); actual *P*‐values are indicated.

Given the correlation of HPK1 expression with systemic NK cell dysfunction in mouse tumor models, we next evaluated the clinical relevance of our results in peripheral NK cells from MP. To assess NK cell effector functions, we used 3 different target cells such as conventional K562 and 721.221 cells, or mouse P815 cells expressing human ULBP1 (a ligand for NKG2D) and CD48 (a ligand for 2B4) for a defined and uniform stimulation.^[^ ^18^, ^23^ ^]^ We compared the effector functions of NK cells from MP with those of healthy controls (HC) using peripheral blood mononuclear cell (PBMC) samples. NK cells from the MP group exhibited a significant impairment in CD107a^+^ cytotoxic degranulation compared with those from the HC group in response to all 3 target cells (**Figure** **6**a,b). Likewise, NK cells from the MP group produced significantly less IFN‐γ than those from the HC group, as measured by intracellular cytokine staining (Figure 6c,d). Thus, on a per‐cell basis, NK cells from the MP group were defective in their capacity to trigger cytotoxic degranulation and IFN‐γ production. However, the frequencies of NK cells (CD3^−^CD56^+^) were comparable between the 2 groups (Figure S16a,b, Supporting Information). NK cell activation against cancer cells is triggered by diverse activating receptors that are often downregulated as a mechanism of systemic NK cell dysfunction in various cancers.^[^ ^2^, ^55^ ^]^ Thus, we assessed the expression of diverse activating receptors on NK cells and found a slight decrease but no significant difference in the expression of NKG2D, 2B4, NKp30, NKp46, or DNAM‐1 on NK cells between the 2 groups (Figure S16c,d, Supporting Information). The lack of correlation between the level of NKG2D and 2B4 and NK cell function in the MP group following activation via NKG2D and 2B4 suggests that mechanisms other than receptor downregulation contribute to systemic NK cell dysfunction in these patients. We next assessed the HPK1 expression level to determine its association with NK cell dysfunction. Compared with the HC group, the MP group exhibited a significant upregulation of HPK1 in NK cells, as determined by flow cytometry (Figure 6e,f). Moreover, HPK1 expression inversely correlated with the frequency of CD107a‐positive or IFN‐γ‐positive NK cells from the 2 groups in response to all 3 target cells (Figure 6g,h). Thus, these results indicate an aberrant upregulation of HPK1 in melanoma patients and its close correlation with NK cell dysfunction.

### 2.7 HPK1 Expression is Linked to TGF‐β level, NK Cell Exhaustion, Survival, and Immunotherapy Response in Melanoma Patients

**Figure 7:**
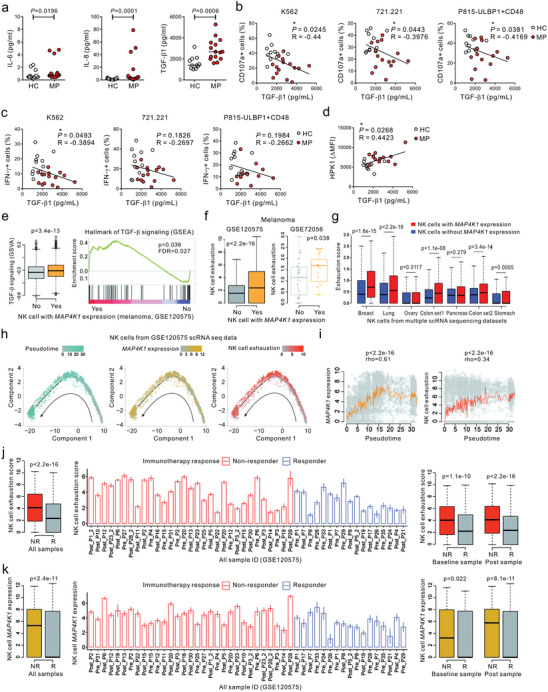
HPK1 expression in NK cells correlates with TGF‐β1 level, NK cell exhaustion, and response to checkpoint immunotherapy. a) The plasma levels of IL‐6, IL‐8, and TGF‐β1 were measured with a Luminex multiplex assay or ELISA in the HC (*n* = 19 for IL‐6 and IL‐8; *n* = 11 for TGF‐β1) and MP group (*n* = 21 for IL‐6 and IL‐8; *n* = 15 for TGF‐β1) from a single experiment. b) The plasma levels of TGF‐β1 correlate inversely with the percentages of CD107a‐positive NK cells after stimulation with all 3 target cells. c) Inverse correlation between plasma TGF‐β1 level and the percentages of IFN‐γ‐positive NK cells after stimulation with K562 cells. d) Positive correlation between HPK1 expression (ΔMFI) in NK cells and plasma TGF‐β1 level. e) NK cells with *MAP4K1* expression in melanoma tissues were significantly enriched in the TGF‐β signaling pathway compared to those in NK cells with no *MAP4K1* expression (GSVA and GSEA analysis). f) NK cells with *MAP4K1* expression exhibited significantly increased exhaustion signatures (*PDCD1*, *TIGIT*, *HAVCR2*, and *LAG3*) in 2 independent melanoma datasets. g) The higher NK cell exhaustion score in NK cells with *MAP4K1* expression was identified in multiple cancer types from 7 scRNA‐seq datasets. h) Trajectory analysis using melanoma scRNA‐seq data reveals that NK cell exhaustion increases as NK cells differentiate toward *MAP4K1* expression. i) *MAP4K1* expression and NK cell exhaustion were upregulated with pseudotime in melanoma. j) NK cell exhaustion level was significantly higher in melanoma samples from non‐responders to checkpoint immunotherapy. k) NK cells with *MAP4K1* expression significantly increased in melanoma samples from non‐responders to checkpoint immunotherapy. Horizontal bars indicate the medians (a); each dot represents an individual donor. Data were analyzed using the Mann‐Whitney U test (a), Spearman correlation test (b‐d, i), and Wilcoxon rank sum test (e‐g, j, and k); actual *P*‐values are indicated.

As the compromised function of NK cells in the MP group was unrelated to cognate receptor downregulation, we speculated the involvement of soluble mediator(s). We measured the levels of soluble cytokines implicated in NK cell dysfunction in the plasma of the study groups using a cytokine multiplex assay. Compared with the HC group, the MP group had significantly higher levels of IL‐6, IL‐8, and, most notably, TGF‐β1, a potent suppressor of NK cell functions^[^ ^56^, ^57^ ^]^ (**Figure** **7**a). By comparison, the levels of other cytokines including IL‐1β, TNF‐α, IL‐10, IL‐12, IL‐15, IL‐18, MIP‐1α, and CXCL1 were downregulated, comparable, or below the detection limit (Figure S17, Supporting Information). Of note, TGF‐β1 level inversely correlated with the frequencies of CD107a‐positive NK cells in response to all 3 target cells (Figure 7b) and of IFN‐γ‐positive NK cells following stimulation with K562 (Figure 7c). By contrast, TGF‐β1 levels positively correlated with HPK1 expression (Figure 7d), implying TGF‐β1 as a potential link between HPK1 upregulation and NK cell dysfunction. In support, treatment with TGF‐β1 impaired cytotoxicity of NK92 cells against K562 target cells (Figure S18a, Supporting Information) and led to the upregulation of HPK1 expression in NK92 cells and CD3^−^CD56^+^ NK cells (Figure S18b,c, Supporting Information), suggesting a possibility of direct contribution of TGF‐β1 to the HPK1 upregulation.

To confirm the clinical relevance of experimental findings in patient cancer tissues, public scRNA‐seq data from several cancer types including malignant melanoma were used. The TGF‐β signaling pathway was significantly enriched in NK cells expressing *MAP4K1* compared to that in NK cells with undetectable *MAP4K1* expression using scRNA‐seq data from malignant melanoma tissues (Figure 7e). Moreover, NK cells with *MAP4K1* expression exhibited significantly increased exhaustion signature (*PDCD1*, *TIGIT*, *HAVCR2*, and *LAG3*) in melanoma tissues (GSE120575 and GSE72056) (Figure 7f). This finding was identified in other multiple solid tumors (Figure 7g), suggesting that the association between *MAP4K1* expression and NK cell exhaustion is present regardless of tumor type. Trajectory analysis revealed that NK cell differentiation exhibited increased exhaustion as the *MAP4K1* was expressed (Figure 7h,i). To determine whether NK cell exhaustion or *MAP4K1* expression correlates with checkpoint immunotherapy response in melanoma, scRNA‐seq data with clinical information (GSE120575) were used. These analyses revealed a significant association between NK cell exhaustion and non‐response to checkpoint immunotherapy in melanoma at both baseline and post‐treatment samples (Figure 7j). Malignant melanoma that did not respond to immunotherapy had more NK cells expressing *MAP4K1* (Figure 7k). These in silico results supported our experimental findings that HPK1 upregulation correlates with NK cell dysfunction and indicated that HPK1 may be a key signaling checkpoint for the anti‐metastatic effects of NK cells and response to immunotherapy.

Finally, we evaluated the effect of NK cell HPK1 expression on patient survival in association with TGF‐β signaling using a refined NK cell scoring system^[^ ^58^ ^]^ and RNA‐seq data with clinical information from The Cancer Genome Atlas (TCGA). We analyzed 76 primary cutaneous melanoma, 365 metastatic cutaneous melanoma, and 8469 pan‐cancer data from the TCGA project (Figure S19, Supporting Information). These analyses revealed that NK score positively correlated with favorable prognosis, whereas *MAP4K1* expression was associated with unfavorable prognosis in contrast to NK score (Figure S19a, Supporting Information). When patients were stratified into 4 groups based on the median values of NK score and *MAP4K1* expression, the group showing low NK score and high *MAP4K1* expression demonstrated the worst prognosis. Consistent with an association between TGF‐β and HPK1 upregulation, *MAP4K1* expression positively correlated with an upregulation of TGF‐β signaling in NK cells. Of note, the survival effect of *MAP4K1* expression in relation to TGF‐β signaling became more significant in metastatic cutaneous melanoma compared to primary cutaneous melanoma (Figure S19b, Supporting Information). Moreover, we validated these findings using large‐scale TCGA pan‐cancer data (Figure S19c, Supporting Information), corroborating the results obtained with the experiments and single‐cell RNA‐seq datasets and supporting the therapeutic value of HPK1 in the context of NK cell‐mediated antitumor immunity.

## 3 Discussion and Conclusion

Accumulating evidence has unveiled the perturbation of systemic as well as local immunity in the TME during metastatic tumor progression.^[^ ^1^, ^2^, ^3^ ^]^ These alterations to the peripheral immune environment include dysregulated function and/or frequency of multiple immune lineages including NK cells in tumor‐burdened patients and mice across multiple organs and blood.^[^ ^8^, ^59^ ^]^ NK cells play a pivotal role in the surveillance against cancer metastasis rather than primary tumor growth.^[^ ^13^, ^14^ ^]^ This specialization of the anti‐metastatic role is supported by the fact that NK cells are abundant in the blood and BM as well as perivascular spaces of diverse organs including the lung that are frequently seeded by metastatic cancer cells.^[^ ^11^ ^]^ Further, NK cells constitute a minor fraction of tumor‐infiltrating immune cells compared with other cytotoxic lymphocytes such as CD8^+^ T cells.^[^ ^13^, ^14^ ^]^ Studies thus far have focused on understanding the mechanisms by which NK cells become dysfunctional within the TME via cancer cell‐mediated dysregulation of NK cell receptors or exposure to inhibitory molecules produced by cancer cells.^[^ ^19^, ^60^ ^]^ Thus, studying the molecular mechanisms driving systemic alterations of NK cells linked to metastatic progression is relevant, given their primary role in preventing metastasis and the correlation between circulating NK cell dysfunction and metastatic burden. Herein, we first demonstrated that HPK1 expression is aberrantly upregulated in dysfunctional NK cells in the periphery and metastasis‐bearing lungs of mice as well as in peripheral blood from melanoma patients. Importantly, we provided evidence that conditional HPK1 overexpression in NK cells exacerbates metastatic spread of malignant melanoma to multiple distant organs including the lung, but not primary tumor growth, via a significant effect on NK cell expansion and cytotoxicity according to HPK1 expression levels. Consistently, HPK1 deficiency rendered NK cells hyperreactive to target cells and thereby conferred resistance to melanoma metastasis in vivo in synergy with a checkpoint blockade of PD‐1 and CTLA‐4. Furthermore, the clinical relevance of this finding was supported by our identification of HPK1 as a critical regulator of human NK cell function and its correlation with NK cell dysfunction and patient response to checkpoint immunotherapy.

HPK1, encoded by *MAP4K1*, is a serine/threonine kinase that is predominantly expressed in hematopoietic cell lineages and functions as a negative regulator of T cells, B cells, and dendritic cells.^[^ ^33^, ^47^, ^61^ ^]^ Upon TCR engagement, HPK1 is activated and mediates the phosphorylation of SLP‐76 at serine 376, leading to the destabilization of the TCR signaling complex and impediment of T cell activation and proliferation.^[^ ^33^, ^47^ ^]^ HPK1 modulates various downstream signaling pathways including MAPK ERK and JNK, AP‐1, and NF‐κB that are involved in cellular proliferation, apoptosis, and immune cell activation.^[^ ^32^, ^62^ ^]^ Germline knockout of *MAP4K1* in mice results in enhanced TCR signaling and antitumor T cell responses to repress primary tumor growth, which is primarily dependent on the kinase activity.^[^ ^49^, ^52^ ^]^ In multiple myeloma (MM) patients, HPK1 expression is upregulated in the tumor‐infiltrating T cells and the implication of HPK1 in mediating T cell exhaustion via HPK1‐NFκB‐Blimp1 axis is validated in *MAP4K1*‐deficient mice.^[^ ^34^ ^]^ However, no study has assessed the direct role of HPK1 upregulation in the control of tumor progression including metastasis in vivo, focusing on antitumor immunity. In this study, HPK1 was expressed in NK cells but dysregulated in the periphery of mice with metastatic melanoma progression. Using conditional Tg mouse lines with graded overexpression of HPK1 in NK cells, we demonstrated that HPK1 overexpression renders NK cells hyporeactive to activation and proliferation and thereby suffices to exacerbate melanoma metastasis rather than primary tumor growth. HPK1 overexpression mediated NK cell dysfunction without concomitant alteration of diverse surface markers related to functional maturation and activation. Moreover, we observed a significant upregulation of HPK1 in NK cells from melanoma patients, which correlated with NK cell dysfunction but unrelated to the expression of activating receptors. Thus, these results suggested a primary role of HPK1 in regulating NK cell function downstream of activating receptors. Unfortunately, we have not yet ascertained the exact mechanism underlying HPK1‐mediated NK cell dysfunction. In our analysis of scRNA‐seq data from malignant melanoma (GSE120575), Blimp1 (*PRDM1*) expression correlated with NK cell exhaustion, non‐response to checkpoint immunotherapy, and *MAP4K1* expression (Figure S20a–d, Supporting Information). However, in the analysis of TCGA RNA‐seq data of metastatic melanoma, the expression of *MAP4K1* but not *PRDM1* was significantly associated with poor prognosis despite their correlated expression (Figure S20e, Supporting Information), suggesting a conserved and divergent role of Blimp1 and HPK1 in NK cells. Unlike T cells, NK cells exhibit fundamental differences in the transcriptional network for the regulation of Blimp1 expression, and Blimp1 is largely dispensable for NK cell effector functions.^[^ ^63^ ^]^ In this regard, it merits further mechanistic studies including the role of Blimp1 in HPK1‐mediated NK cell dysfunction.

Cancer cells, including metastatic melanoma, evolve mechanisms to evade or impair immunosurveillance by NK cells, including the release of soluble factors.^[^ ^19^, ^64^ ^]^ Circulating cytokines and chemokines derived from primary and metastatic tumors can trigger peripheral immune perturbations that coincide with malignant tumor outgrowth.^[^ ^65^ ^]^ Although it remains unclear the mechanisms underlying HPK1 upregulation in peripheral NK cells, we speculate that inflammation is likely a contributing factor. This notion is supported in part by a study on the downregulated expression of HPK1 in overactive CD4^+^ T cells from patients with autoimmune disease (i.e., systemic lupus erythematosus).^[^ ^66^ ^]^ Systemic inflammation and immunosuppression have been associated with an increased risk of metastatic disease and poor patient outcomes.^[^ ^18^, ^67^ ^]^ In this study, we observed significantly elevated levels of TGF‐β1 along with IL‐6 and IL‐8 in the plasma of MP. Furthermore, TGF‐β1 level correlated inversely with NK cell function but positively with HPK1 expression, suggesting HPK1 as a potential mechanistic link between TGF‐β1 and NK cell dysfunction. Supporting this, blood TGF‐β1 levels are significantly elevated in patients with lung, colorectal, melanoma, pancreatic cancers (PC), and MM compared with those in HC and involved in the dysfunction of patient NK cells.^[^ ^18^, ^23^, ^68^ ^]^ Furthermore, we observed an upregulation of HPK1 in dysfunctional NK cells from PC patients (unpublished result) and following treatment with TGF‐β1. TGF‐β1 is a major cytokine that impairs NK cell‐mediated cancer immunosurveillance.^[^ ^56^ ^]^ The repression of IL‐15‐induced mTOR activation by TGF‐β1 is an essential mechanism to inhibit the proliferation and cytotoxic activity of NK cells.^[^ ^57^ ^]^ Herein, HPK1 upregulation in correlation with TGF‐β1 was likely an additional layer of the mechanism of immune evasion by tumors to inhibit NK cell function by targeting signaling via activating receptors. However, factors other than TGF‐β1 might have been involved, which merits further investigation.

Ameliorating NK cell dysfunction and enhancing its effector function are the desired outcomes of NK cell‐based cancer immunotherapy, particularly for the control of metastatic diseases.^[^ ^13^ ^]^ Currently, immune checkpoint receptors such as PD‐1 and CTLA‐4 have been explored as promising therapeutic targets to treat metastatic cancer by enhancing antitumor immunity, including that mediated by NK cells.^[^ ^4^, ^69^ ^]^ Our studies indicate the therapeutic potential of targeting HPK1 in NK cell‐directed therapies treating metastatic cancer in combination with ICT. Supporting this, HPK1 expression in NK cells is associated with the response to checkpoint immunotherapy. We previously showed that the regulation of NK cell function by SLP‐76 is common to diverse activating receptors that couple to both ITAM and non‐ITAM signaling pathways.^[^ ^12^, ^51^ ^]^ Thus, HPK1 may serve as an intracellular checkpoint molecule for NK cell activation by mediating the inhibitory phosphorylation of SLP‐76 at serine 376. Moreover, as NK cells and T cells express HPK1 and have complementary roles in tumor immunity,^[^ ^11^ ^]^ a blockade of HPK1 in both cell types (e.g., kinase inhibitor drugs) can lead to combined therapeutic effects. Antitumor immunity is often compromised by tumor‐derived suppressive factors such as TGF‐β, PGE2, and adenosine, which represent a major source of resistance to immunotherapies.^[^ ^70^ ^]^ The depletion and inhibition of HPK1 renders T cells resistant to such immunosuppression.^[^ ^52^, ^71^ ^]^ Thus, HPK1 modulation may help overcome immunosuppression and extend the effector function of NK cells. However, its therapeutic utility including the use of HPK1 inhibitor remains to be determined in various types of metastatic disease, alone or in combination with ICT.

Although we identified HPK1 as a pivotal regulator of NK cell‐mediated anti‐metastatic function, this study has some limitations. We cannot rule out the contribution of other regulatory mechanisms to NK cell dysfunction besides HPK1 during metastatic progression, given our focus on genes linked to the MAPK signaling pathway. Similar to TGF‐β1, activin‐A, a TGF‐β superfamily member, also impairs NK cell function and proliferation in a SMAD2/3‐dependent mechanism.^[^ ^72^ ^]^ Thus, the involvement of other cytokines in HPK1 upregulation and NK cell dysfunction cannot be excluded. While we demonstrated NK cells as key effectors in preventing lung metastasis of B16F10 cells^[^ ^25^ ^]^ and enhanced cytotoxicity of NK cells in *MAP4K1* KO mice, HPK1 may also affect other leukocytes linked to metastatic outcomes. In this regard, it warrants further investigation using *MAP4K1* conditional KO mouse targeting specific cell types to confirm our results. The size of the patient cohort and public RNA‐seq data from malignant melanoma used in this study are not sufficient to establish a definitive relationship among HPK1 expression, NK cell dysfunction, and metastatic melanoma progression for clinical translation despite their significant association in our study. Accordingly, it merits further study with the inclusion of a sufficient number of cases and public datasets including different cancer stages, metastatic sites, expression levels of HPK1, and patient outcomes for an additional in silico analysis and experimental validation.

In summary, we herein demonstrate HPK1 upregulation during metastatic melanoma progression as a previously unappreciated aspect of systemic NK cell dysfunction by impacting activating receptor pathways. Such regulation by tumors may “hijack” and “amplify” the normal negative regulatory function of HPK1 as a mechanism of tumor immune escape for metastasis. The pathophysiological relevance was supported by our analyses of peripheral blood samples and public RNA‐seq data from melanoma patients. Our findings also propose HPK1 as a promising target to optimize the anti‐metastatic effect of NK cells and its expression in peripheral NK cells as a predictive prognostic marker.

## 4 Experimental Section

### Cell Culture and Reagents

B16F10, B16F10‐Luc2, and B16F1 melanoma cell lines (ATCC) and LL/2 (LLC1)‐Luc2 Lewis lung carcinoma cell line (ATCC) were cultured in DMEM containing 10% FBS and harvested for tumor model experiments using Detachin cell detachment solution (Gelantis). TC‐1 lung cancer cell line was cultured in RPMI1640 medium containing 10% FBS and 2 mM L‐glutamine.^[^ ^25^ ^]^ YAC‐1 (ATCC), RMA, and RMA‐s murine lymphoma cell lines were cultured in RPMI1640 medium containing 5% FBS and 2 mM L‐glutamine. P815 (ATCC) and 721.221 cell lines were cultured in IMDM medium containing 10% FBS and 2 mM L‐glutamine. The human NK cell line NKL (gift of M. Robertson) was cultured in RPMI medium containing 10% FBS, 1 mM sodium pyruvate, and 200 U mL^−1^ recombinant IL‐2. NK92 cell line (gift of K. S. Campbell) was cultured in α‐MEM medium containing 20% FBS, 1% MEM vitamin solution, 0.1 mM 2‐mercaptoethanol, and 200 U mL^−1^ recombinant IL‐2. To measure the effector function of NK cells from melanoma patients, three different target cells were used: conventional K562 (ATCC) and 721.221 cells (gift of J. Gumperz and P. Parham) as well as mouse P815 cells (ATCC) with stable surface expression of ULBP1 (a ligand for human NKG2D) and CD48 (a ligand for human 2B4) for defined and uniform stimulation.^[^ ^18^ ^]^ K562, 721.221, and P815‐ULBP1+CD48 cells were cultured in IMDM supplemented with 10% FBS and 2 mM L‐glutamine. Plat‐A retroviral packaging cell line (Cell Biolabs) was cultured in DMEM containing 10% FBS, 1 µg/mL puromycin, and 10 µg/mL blasticidin. DsRed‐tagged B16F10 cells were previously described^[^ ^25^ ^]^ and used to assess spontaneous lung metastasis. TC‐1‐Luc cells were generated by transducing TC‐1 cells with firefly luciferase retroviral construct. Retroviral particles were produced by transfection of the Plat‐A retroviral packaging cell line with pQCXIP‐F‐luc (Addgene #73 058). TC‐1 cells were transduced with virus‐containing supernatant by spinfection in the presence of 10 µg/mL polybrene. Thereafter, TC‐1 transductants were selected by 3 µg/mL puromycin and grown as pure cultures. The cells were confirmed to be free of mycoplasma contamination. Recombinant human IL‐2 was obtained from Roche, recombinant mouse IL‐15/IL‐15R complex from eBioscience, recombinant human TGF‐β1 from PeproTech and carboxyfluorescein succinimidyl ester (CFSE), CellTrace Far Red cell proliferation kit, Live/Dead Fixable Green Dead Cell Stain, and Live/Dead Fixable Aqua Dead Cell Stain from Molecular Probes.

### Mice

C57BL/6 mice were purchased from Koatech (Gyeong‐gi, Korea). *Ncr1* ^iCre^ knock‐in mice on a C57BL/6 background^[^ ^40^ ^]^ were kindly provided by Prof. Eric Vivier (Aix‐Marseille University, France) via The European Mouse Mutant Archive. *Ncr1* ^iCre^ genotyping was carried out using the following PCR primers: NKp46icreF, 5′‐GGAACTGAAGGCAACTCCTG‐3′; NKp46icreKIR, 5′‐CCCTAGGAATGCTCGTCAAG‐3′; NKp46icreWTR, 5′‐TTCCCGGCAACATAAAATAAA‐3′; with expected product sizes of 300 bp for the WT allele and 247 bp for the modified allele. *Ncr1* ^iCre^ mice were crossed with conditional *MAP4K1* transgenic mice (*MAP4K1* ^Tg^) to generate *Ncr1* ^iCre^ or *Ncr1* ^iCre^ *MAP4K1* ^Tg^ mice. Six to nine‐week‐old littermates with a matched age and sex were used for tumor model experiments. Animals were fed ad libitum and kept under specific pathogen‐free conditions. All experimental protocols were approved by the Institutional Animal Care and Use Committee of the Asan Institute for Life Sciences (Approval No. 2021‐13‐122). For in vivo experiments, investigators were blinded to the genotype of the experimental groups.

### Generation of Conditional *MAP4K1* Transgenic Mice

To generate a conditional transgene expression vector, a pCAG‐19‐LSL‐tdTomato vector was constructed by modifying the Ai9 plasmid (Addgene # 22 799).^[^ ^38^ ^]^ Briefly, after successively deleting the diphtheria toxin fragment A cassettes using SgrDI and XhoI restriction enzymes and the NeoR cassette using SmaI and AscI restriction enzymes from the Ai9 plasmid, the PmeI and SpeI‐digested 5.1 kb DNA fragment was isolated and subcloned into the XbaI and HincII sites of the pUC19 cloning vector to generate the pCAG‐19‐LSL‐tdTomato vector. After the tdTomato cDNA was removed by FseI digestion from the pCAG‐19‐LSL‐tdTomato vector, a full‐length mouse *MAP4K1* cDNA obtained by PCR amplification and FseI digestion was inserted into the vector. Constructs were verified by Sanger sequencing at Macrogen Incorporate (Seoul, Korea). The linearized transgene was prepared by isolating the DrdI‐digested 6.9‐kb DNA fragment. All restriction enzymes, except for SgrDI (Thermo Scientific, Inc.), were purchased from New England BioLabs, Inc. The dependence of transgene expression on the Cre recombinase was tested by transfection of the circular form or linearized transgene constructs into 293T cells with or without the Cre recombinase construct, and cell lysates were immunoblotted for HPK1 or β‐actin. After validation of Cre‐mediated HPK1 (encoded by *MAP4K1*) protein expression from the transgene, standard techniques were used to generate the transgenic mice. In brief, the linearized transgene was injected into the pronuclei of fertilized eggs of C57BL/6N mice. The injected eggs were then transferred into the oviducts of pseudopregnant ICR mice. Founder mouse screening and routine genotyping were conducted by polymerase chain reaction (PCR) using genomic DNAs extracted from tail biopsies as templates and primer pairs common for the mouse *MAP4K1* gene and the transgene: 5′−GGAGGAATTAGGGACGATGGG−3′ and 5′−TCCTTCCTTGGGGGTACGA−3′ (WT, 271 bp; Tg, 197 bp).

### Generation of *MAP4K1*‐Deficient Mice

The *Map4k1*‐deficient mouse model was established by CRISPR/Cas9‐mediated gene targeting (Figure S12, Supporting Information). The genomic DNA sequence of mouse *Map4k1* gene (NC_000073.7 REGION: 28 681 475.28702704) was analyzed using SnapGene software (Dotmatics, USA), and to target an asymmetric exon 4 (91 bp, transcript_id = XM_0 065 39999.1) present in all annotated *Map4k1* transcripts is determined. To select single guide RNAs (sgRNAs) having minimal off‐target effects, the exon 4 sequence was analyzed using the Benchling software (https://benchling.com/) and selected *Map4k1*‐RG1 and *Map4k1*‐RG2 exhibiting possible off‐target sites with 3 or more mismatches. To generate sgRNAs, the following oligomers were annealed and cloned into the BsaI sites of the pUC57‐sgRNA vector (Addgene #51 132):^[^ ^73^ ^]^ 5′−TAGGGAGAATCTCCTTCTGAAGGG−3′ and 5′−AAACCCCTTCAGAAGGAGATTCTC−3′ for *MAP4K1*‐RG1; 5′−TAGGAAGCCACGATGTTGGCGTGC−3′ and 5′−AAACGCACGCCAACATCGTGGCTT−3′ for *MAP4K1*‐RG2. Constructs were verified by Sanger sequencing at Macrogen Incorporate (Seoul, Korea). For the in vitro transcription, the template DNA was PCR‐amplified from the cloned plasmids using a pair of primers specific for the pUC57‐sgRNA vector (5′−GTAAAACGACGGCCAGT−3′ and 5′−GCACCGACTCGGTGCCACT−3′), and the sgRNA was synthesized using the MEGAshortscript T7 kit (Ambion) according to the manufacturer's instructions. CRISPR/Cas9‐mediated gene targeting in mice was performed as described previously.^[^ ^74^ ^]^ Briefly, *Streptococcus pyogenes Cas9* mRNA was prepared by in vitro transcription as described.^[^ ^74^ ^]^ In vitro transcribed RNAs were dissolved in diethyl pyrocarbonate (DEPC, Sigma)–treated injection buffer (0.25 mM EDTA, 10 mM Tris, pH 7.4). C57BL/6N (B6N) and ICR mice (OrientBio, Korea) were used as zygote donors and foster mothers, respectively, and were prepared as previously described.^[^ ^75^ ^]^ 2 sgRNAs, *Map4k1*‐RG1 (250 ng/µL) and *Map4k1*‐RG2 (250 ng µL^−1^) were simultaneously microinjected with *Streptococcus pyogenes Cas9* mRNA (50 ng µL^−1^) into the cytoplasm of mouse zygotes from B6N, and the manipulated embryos were transferred into the oviducts of ICR foster mothers. Genomic DNA samples were prepared from tail biopsies of newborn mice, and PCR genotyping was conducted using the following oligomers: 5′−CCTCCCCACAGATGATGACG−3′ and 5′−GTTGGGGGATGGGCTAAGAC−3′ (127 bp). Mutant sequences were identified by Sanger sequencing at Macrogen Incorporate (Seoul, Korea) after cloning the amplicons using a T‐Blunt PCR Cloning Kit (SolGent Co., Ltd. Korea).

### Healthy Control and Patient Samples

Among the patients admitted to the Department of Dermatology of the Asan Medical Center, 22 patients with malignant melanoma who consented to participate in the study were included, between September 2019 and August 2022. Peripheral blood samples were collected, and PBMCs and plasma were harvested within 1 h and then cryopreserved until use as described.^[^ ^18^ ^]^ The diagnosis, tumor size, laboratory test, pathological characteristics, melanoma subtype, AJCC stage, and treatment and follow‐up data were determined by pathologic examination. As a control, 19 healthy volunteers were included. The clinical and demographic characteristics of patients are summarized in Tables S1 (Supporting Information). The study complied with the Declaration of Helsinki, and the protocol was reviewed and approved by the Institutional Review Board (IRB) of Asan Medical Center (IRB No. 2019–1238).

### Mouse Tumor Model

To generate mouse models of primary tumor growth along with spontaneous metastasis, 1 × 10^6^ DsRed‐tagged B16F10 cells or B16F10 cells in 100 µL Dulbecco's phosphate‐buffered saline (DPBS) were subcutaneously injected into the shaved flank of mice as described.^[^ ^25^ ^]^ Tumor size was measured using digital calipers and calculated as V = (L × W^2^)/2. After 3 weeks of primary tumor growth, spontaneous lung metastases were assessed by *ex vivo* fluorescence imaging of excised lungs using the IVIS Lumina II system (PerkinElmer). For experimental metastasis, luciferase‐tagged B16F10‐Luc2 cells, LL/2 (LLC1)‐Luc2 cells, or TC‐1‐Luc cells (5 × 10^5^ cells) in 200 µL DMEM medium containing 5% FBS were injected into mice via the tail vein as described.^[^ ^25^ ^]^ Metastases in the lung and other organs were assessed by flow cytometric and bioluminescent imaging analyses on the indicated days after tumor cell injection. Luminescence was normalized to day 0 for each mouse, to account for any differences in injection efficiency of tumor cells between mice. To assess the effect of HPK1 overexpression on spontaneous lung metastasis, luciferase‐tagged B16F10‐Luc2 cells (1 × 10^6^ cells) were subcutaneously injected into the shaved flank of *Ncr1* ^iCre^ or *Ncr1* ^iCre^ *MAP4K1* ^Tg^ mice. The primary tumor size was measured using digital calipers every other day, and pulmonary metastases were assessed by *ex vivo* bioluminescent imaging of excised lungs on day 20 post‐injection of tumor cells using IVIS Lumina II system. To assess the effect of HPK1 deficiency in combination with ICT, WT and *MAP4K1* KO mice received intraperitoneal injection of control IgG (500 µg) or a combination of anti‐PD‐1 (250 µg; clone RMP1‐14; Bio X Cell) and anti‐CTLA‐4 (250 µg; clone UC10‐4F10‐11; Bio X Cell) on days 0 and 3 after intravenous injection of B16F10‐Luc2 cells (5 × 10^5^ cells) as described.^[^ ^76^ ^]^ Pulmonary metastases were assessed on day 12 after tumor cell injection using an IVIS Lumina II system.

### NK Cell Depletion

To deplete NK cells, mice received intraperitoneal injections of anti‐NK1.1 antibody (150 µg; clone PK136; Bio X Cell) while the control mice were injected with matching isotype mouse IgG2a (150 µg; clone C1.18.4; Bio X Cell). The injections were performed 1 day before intravenous injection of B16F10 cells and then twice a week until euthanasia. The depletion of NK cells was confirmed by flow cytometry.

### NK Cell‐Mediated Lymphoma Clearance Assay

To assess whether systemic NK cell activity in vivo was affected by melanoma lung metastasis, a lymphoma clearance assay was performed as previously described.^[^ ^25^ ^]^ Briefly, MHC class‐I‐sufficient (RMA) or ‐deficient (RMA‐s) lymphoma cells were labeled with 1 and 4 µM CFSE, respectively. The cells were mixed in a 1:1 ratio (1 × 10^6^ cells per cell type) and injected i.p. into control mice or mice with melanoma lung metastasis. After 6 h post‐challenge of RMA and RMA‐s cells, the rejection of NK cell‐sensitive RMA‐s cells relative to NK cell‐resistant RMA cells in the peritoneal cavity was measured using flow cytometry and calculated as follows: 1–([CFSE^low^/CFSE^high^]input/[CFSE^low^/CFSE^high^]output) × 100%.

### Flow Cytometric Analysis of Mouse NK Cells

Single‐cell suspensions from the spleen, lung, blood, and BM were prepared as described.^[^ ^25^ ^]^ Isolated cells were washed, treated with RBC lysis buffer (eBioscience), and filtered through a 70 µm strainer. To analyze NK cell populations, cell suspensions were blocked with mouse BD Fc Block (clone 2.4G2; 1:50 dilution; 553 142), and CD3ε^−^NK1.1^+^ or NK1.1^+^CD49b^+^ NK cells were identified after lymphocyte gating. To analyze different leukocyte populations including NK cells, cell suspensions were blocked with mouse BD Fc Block (clone 2.4G2; 1:50 dilution; 553 142), incubated with Live/Dead Fixable Aqua Dead Cell Stain (Molecular Probes; 1:200 dilution; L34957), and then incubated with fluorochrome‐conjugated antibodies. The following antibodies were used for staining: anti‐CD45‐APC‐eFluor 780 (eBioscience; clone 30‐F11; 1:50 dilution; 47–0451), anti‐CD11b‐PE‐Cy7 (BD Biosciences; clone M1/70; 1:100 dilution; 552 850), anti‐Ly6G‐PerCP‐Cy5.5 (BD Biosciences; clone 1A8; 1:100 dilution; 560 602), anti‐Ly6C‐APC (BD Biosciences; clone AL‐21; 1:100 dilution; 560 595), anti‐F4/80‐FITC (eBioscience; clone BM8; 1:100 dilution; 11–4801), anti‐CD11c‐PE (BD Biosciences; clone HL3; 1:100 dilution; 557 401), anti‐MHC II‐PerCP‐Cy5.5 (BD Biosciences; clone M5/114.15.2; 1:100 dilution; 562 363), anti‐CD206‐APC (BioLegend; clone C068C2; 1:100 dilution; 141 707), anti‐CD3ε‐PerCP (BD Biosciences; clone 145‐2C11; 1:100 dilution; 553 067), anti‐NK1.1‐PE (BD Biosciences; clone PK136; 1:100 dilution; 557 391), anti‐CD27‐FITC (eBioscience; clone LG.7F9; 1:100 dilution; 11–0271), anti‐CD4‐APC (BD Biosciences; clone RM4‐5; 1:100 dilution; 553 051), anti‐CD8a‐PE (BD Biosciences; clone 53–6.7; 1:100 dilution; 553 032), anti‐γδTCR‐FITC (BD Biosciences; clone GL3; 1:100 dilution; 561 996), anti‐CD49b‐PE (BD Biosciences; clone DX5; 1:100 dilution; 553 858), anti‐NKp46‐PE (BD Biosciences; clone 29A1.4; 1:100 dilution; 560 757), anti‐CD107a‐FITC (BD Biosciences; clone 1D4B; 1:100 dilution; 553 793), anti‐MHC I‐PE (BD Biosciences; clone AF6‐88.5; 1:100 dilution; 553 570), anti‐KLRG1‐FITC (BioLegend; clone 2F1; 1:100 dilution; 138 410), anti‐NKG2A/C/E‐FITC (BD Biosciences; clone 20d5; 1:100 dilution; 550 520), anti‐CD314/NKG2D‐PE (BD Biosciences; clone CX5; 1:100 dilution; 558 403), anti‐CD122‐FITC (BD Biosciences; clone TM‐β1; 1:100 dilution; 553 361), anti‐CD226/DNAM‐1‐PE (BioLegend; clone 480.1; 1:100 dilution; 132 006), mouse IgG1 isotype control (BD Biosciences; clone MOPC‐21), and mouse IgG2a isotype control (BD Biosciences; clone G155‐178). Labeled cell populations were measured using a BD Accuri C6 Plus (BD Biosciences) or BD FACSCanto II (BD Biosciences), and data were analyzed using FlowJo version 10 (Tree Star). Pre‐gating on single‐cell, viable, and CD45^+^ cell populations was applied to all the immunophenotyping analyses of immune cells including NK cells. Myeloid cell populations were identified as CD45^+^CD11b^+^Ly6G^+^Ly6C^int^ neutrophils, CD45^+^CD11b^+^Ly6G^−^Ly6C^+^ monocytes, CD45^+^CD11c^+^F4/80^−^MHCII^+^ dendritic cells, or CD45^+^CD11b^+^F4/80^+^MHCII^+^ M1‐like and CD45^+^CD11b^+^F4/80^+^CD206^+^ M2‐like macrophages. In addition, lymphocyte cell populations were identified as CD45^+^CD3^−^NK1.1^+^ NK cells, CD45^+^CD3^+^NK1.1^−^ T cells, CD45^+^CD3^+^γδ T cells, CD45^+^CD3^+^CD8^+^ T cells, and CD45^+^CD3^+^CD4^+^ T cells.

### Flow Cytometric Analysis of HPK1 Expression

Single cells from the spleen or lungs of tumor‐burdened mice were isolated, treated with red blood cell lysis buffer (eBioscience), and filtered through a 70 µm strainer (BD Biosciences). Thereafter, cell suspensions were blocked with mouse BD Fc Block (clone 2.4G2; 1:50 dilution; 553 142) and stained with surface marker antibodies [anti‐CD3ε‐PerCP (BD Biosciences; clone 145‐2C11; 1:100 dilution; 553 067), anti‐NK1.1‐PE (BD Biosciences; clone PK136; 1:100 dilution; 557 391), and anti‐CD45‐APC (BioLegend; clone 30‐F11; 1:100 dilution; 103 112)]. After washing with FACS buffer, the cells were incubated in BD Cytofix/Cytoperm solution for 20 min at 4 °C and washed twice with BD Perm/Wash buffer. Thereafter, the cells were blocked with 5% normal goat serum (Invitrogen) in BD Perm/Wash buffer, and stained with normal rabbit IgG (Cell Signaling; 1:100 dilution; 2729) or HPK1‐specific antibody (Cell Signaling; 1:100 dilution; 4472) overnight at 4 °C. After washing twice with BD Perm/Wash buffer, cells were incubated with Alexa Fluor 488‐conjugated goat anti‐rabbit IgG (Jackson ImmunoResearch; 1:500 dilution; 111‐546‐045) for 1 h at 4 °C, washed again, and analyzed using flow cytometry. As a control, HPK1 expression in NKL cells was analyzed with a similar procedure without surface marker staining.

For the analysis of HPK1 expression in human PBMCs, isolated PBMCs were blocked with Human Fc receptor‐binding inhibitor (eBioscience; 1:50 dilution; 14‐9161‐73) and stained with surface marker antibodies [anti‐CD3‐PerCP (clone SK7; 1:100 dilution; 347 344) and anti‐CD56‐PE (clone NCAM16.2; 1:100 dilution; 340 363)]. The cells were washed with FACS buffer and fixed with 4% paraformaldehyde at 37 °C for 10 min. The fixed cells were then permeabilized with 40% methanol on ice for 30 min and blocked with 1% normal goat serum (Invitrogen) in FACS buffer for 30 min at 4 °C. Then, cells were stained with normal rabbit IgG (Cell Signaling; 1:100 dilution; 2729) or HPK1‐specific antibody (Cell Signaling; 1:100 dilution; 4472) for 1 h at RT. After washing, the cells were incubated with Alexa Fluor 488‐conjugated goat anti‐rabbit IgG (Jackson ImmunoResearch; 1:500 dilution; 111‐546‐045) for 1 h at RT, washed again, and analyzed using flow cytometry.

### Mouse NK Cell Degranulation Assay

Cytotoxic degranulation was determined by measuring the cell surface expression of CD107a as described.^[^ ^25^ ^]^ IL‐2‐activated NK cells isolated from splenocytes by negative selection using a mouse NK cell isolation kit (STEMCELL Technologies) were stimulated with B16F10 cells for 4 h. Lymphocytes were gated on forward scatter/side scatter, and the CD107a surface expression on CD3ε^−^NK1.1^+^ NK cells was analyzed by flow cytometry. NK cell degranulation was determined by the percent increase of CD107a^+^ NK cells after stimulation with B16F10 cells relative to CD107a^+^ NK cells without stimulation (ΔCD107a^+^ cells).

### Mouse NK Cell Cytotoxicity Assay

To assess NK cell cytotoxicity in the metastasis‐bearing lungs or splenocytes of mice, CellTrace Far Red‐labeled B16F10 cells (1 × 10^4^ cells) or YAC‐1 cells (1 × 10^4^ cells) were co‐cultured with lung single cells (1 × 10^5^ cells) for 6 h or splenocytes (5 × 10^5^ cells) for 4 h at 37 °C in the 96‐well V bottom plate (Nunc). For the spontaneous death control, CellTrace Far Red‐labeled B16F10 or YAC‐1 cells were cultured alone under the same conditions. Thereafter, the cells were stained with the Live/Dead Fixable Green Dead Cell Stain at RT for 30 min. B16F10 or YAC‐1 cells were gated on forward scatter/side scatter, and the Live/Dead stain‐positive dead cells among Far Red‐labeled B16F10 or YAC‐1 cells were analyzed by flow cytometry.

### Mouse NK Cell Proliferation Assay

To assess in vitro proliferation, NK cells were purified from the BM and spleen by negative selection using a mouse NK cell isolation kit (STEMCELL Technologies) and labeled with 2 µM of CFSE (Invitrogen) in DPBS for 10 min at 37 °C. CFSE‐labeled NK cells (2 × 10^4^ cells/well) were cultured for 72 h at 37 °C in 100 µL of proliferation medium (RPMI1640 supplemented with 10% FBS, 10 mM HEPES, 1 mM nonessential amino acid, 25 µM 2‐mercaptoethanol, and 1 mM sodium pyruvate) with a recombinant mouse IL‐15/IL‐15R complex (eBioscience, herein referred to as IL‐15; 1.25 to 2.5 ng mL^−1^) in 96‐well flat‐bottom plates previously coated with 10 µg/mL isotype‐control antibody or anti‐NK1.1 antibody (PK136). To assess the effect of IL‐15 on NK cell proliferation, CFSE‐labeled NK cells were incubated at 37 °C for 72 h with different concentrations of mouse IL‐15 before flow cytometric analysis. NK cell proliferation was measured by CFSE dilution on CD3ε^−^NK1.1^+^ or NK1.1^+^CD49b^+^ NK cells after Fc receptor blocking and lymphocyte gating.

To assess in vivo proliferation, mice were injected with 200 µg of poly I:C (HMW, Invivogen) or PBS. Thereafter, single‐cell suspensions from the BM and splenocytes were harvested from the mice 18 h post‐injection, blocked with mouse BD Fc Block, and stained with surface marker antibodies (anti‐CD3ε and anti‐NK1.1) for 1 h at 4 °C. After washing with FACS buffer, the cells were fixed, permeabilized, and washed using Foxp3 staining buffer set (eBioscience). Then, the cells were blocked with 2% normal rat serum (Invitrogen) in Perm buffer, and stained with an anti‐Ki‐67‐PE antibody (eBioscience; SolA15; 1:100 dilution; 12‐5698‐82) for 1 h at RT. After washing twice with Perm buffer, the cells were analyzed using flow cytometry.

### Retroviral Transduction of HPK1 WT and Kinase‐Dead Mutant

The plasmid encoding human *MAP4K1* (#23 484) was obtained from Addgene. The *MAP4K1* K46M mutant was generated by a QuickChange site‐directed mutagenesis kit (Stratagene). All constructs were verified by sequencing. To generate a stable human NKL cell line expressing HPK1 WT or kinase‐dead K46M mutant, Plat‐A retroviral packaging cell line was transfected with GFP retroviral vectors (pMX‐IRES‐GFP) that allows for the co‐expression of cloned HPK1 and GFP using X‐tremeGENE 9 (Roche). After medium change 24 h post‐transfection, the virus‐containing supernatant was collected after another 24 h, mixed with fresh medium at a 1:1 ratio, and 10 µg mL^−1^ polybrene and 200 U mL^−1^ rIL‐2 were added to make the transducing mix. NKL cells for transduction (0.5 × 10^6^) were resuspended in 2.4 mL of transducing mix and then transferred to a 12‐well plate. The plate was centrifuged at 700 × g for 30 min at 32 °C and incubated for 3 h at 37 °C. After another centrifugation at 700 × g for 30 min at 32 °C and incubation for 6 h, the cells were washed and resuspended with fresh medium. Three days post‐infection, the cells with a matched level of GFP expression were isolated by a FACS Aria cell sorter.

### Human *MAP4K1* Knockdown

Human NKL cells were transfected with siRNA (300 pmol) with the Amaxa Nucleofector II system, as described.^[^ ^54^ ^]^ NKL cells (1.2 × 10^6^) were resuspended in 100 mL of Amaxa kit solution V (Lonza) and transfected with program O‐017 after mixing with siRNA. After 48 h of incubation at 37 °C in the presence of IL‐2 (200 U mL^−1^), the cells were assayed as indicated. The siRNAs specific for human *MAP4K1* were part of TriFECTa Dicer‐substrate kit obtained from Integrated DNA Technologies (IDT). ON‐TARGETplus SMARTpool siRNAs specific for *MAP4K1* (L‐003586) were also obtained from Dharmacon. Similar results were obtained with either of the siRNAs used. The results shown in this paper were those obtained with the former set of siRNA oligonucleotides. The negative siRNA controls were obtained from IDT and Dharmacon.

### Antibodies for the Flow Cytometric Analysis of Human NK Cells

NK cell function was evaluated using the following fluorochrome‐conjugated monoclonal antibodies (mAbs) by flow cytometry: anti‐CD3‐PerCP (clone SK7; BD Biosciences; 1:100 dilution; 347 344), anti‐CD56‐PE (clone NCAM16.2; BD Biosciences; 1:100 dilution; 340 363), anti‐CD107a/LAMP1‐FITC (clone H4A3; BD Biosciences; 1:50 dilution; 555 800), and anti‐IFN‐γ‐FITC (clone 25 723.11; BD Biosciences; 1:50 dilution; 340 449). The phenotype of NK cells was evaluated using the following fluorochrome‐conjugated mAbs by flow cytometry: anti‐CD3‐PerCP (clone SK7; BD Biosciences; 1:100 dilution; 347 344), anti‐CD56‐FITC (clone NCAM16.2; BD Biosciences; 1:100 dilution; 340 410), anti‐CD314/NKG2D‐PE (clone 149 810; R&D Systems; 1:100 dilution; FAB139P), anti‐CD244/2B4‐PE (clone C1.7; BioLegend; 1:100 dilution; 329 508), anti‐NKp30‐PE (CD337; clone Z25; Beckman Coulter; 1:100 dilution; IM3709), anti‐NKp46‐PE (CD335; clone 9E2; BD Biosciences; 1:100 dilution; 557 991), anti‐DNAM‐1‐PE (CD226; clone DX11; BD Biosciences; 1:100 dilution; 559 789), and mouse IgG1 isotype control (clone MOPC‐21; BD Biosciences; 1:100 dilution; 555 749).

### Cytotoxic Degranulation and Intracellular IFN‐γ Staining Assay of Human NK cells

PBMCs were separated from peripheral blood by density gradient using a lymphocyte separating medium (MP Biomedicals), frozen in FBS (Gibco) containing 10% DMSO (Sigma‐Aldrich), kept at −80 °C for 24 h, and then stored at −196 °C in liquid nitrogen. Cryopreserved PBMCs including paired melanoma patients and healthy controls were thawed rapidly at 37 °C in a single day and suspended in complete RPMI medium (RPMI1640 medium supplemented with 10% FBS, 2 mM L‐glutamine, 100 U mL^−1^ penicillin, and 100 µg mL^−1^ streptomycin in the presence of 50 U mL^−1^ of DNase I (Roche). The cells were washed twice and resuspended with a complete RPMI medium, followed by overnight resting.

Cytotoxic degranulation of NK cells was assessed by CD107a expression on the cell surface, as described.^[^ ^18^ ^]^ Briefly, PBMCs (1 × 10^5^ cells) were mixed with an equal number of K562, 721.221, or P815‐ULBP1+CD48 cells for 2 h at 37 °C. After incubation, the cell pellets were recovered by centrifugation, resuspended in FACS buffer (PBS with 1% FBS), and stained with anti‐CD3‐PerCP, anti‐CD56‐PE, and anti‐CD107a/LAMP1‐FITC for 30 min in the dark at 4 °C. Lymphocytes were identified by forward and side scatter (FSC and SSC) characteristics, and the CD107a expression on CD3^−^CD56^+^ NK cells among lymphocytes was analyzed by flow cytometry using FlowJo software (ver.10, Tree Star).

For the analysis of IFN‐γ production by NK cells, PBMCs (1 × 10^5^ cells) were stimulated with an equal number of the indicated target cells for 1 h at 37 °C. Thereafter, the samples were added with brefeldin A (GolgiPlug; BD Bioscience) and monensin (GolgiStop; BD Bioscience), followed by an additional incubation for 5 h, for a total of 6 h. Then, the cells were first stained with anti‐CD3‐PerCP and anti‐CD56‐PE for 30 min in the dark at 4 °C. After washing twice with a FACS buffer, the samples were fixed and permeabilized with a BD Cytofix/Cytoperm solution (BD Bioscience) for 20 min in the dark at 4 °C. The cells were then washed twice with BD Perm/Wash buffer (BD Bioscience), stained with anti‐IFN‐γ‐FITC overnight in the dark at 4 °C, washed again, and analyzed by flow cytometry gated on CD3^−^CD56^+^ NK cells.

### NK Receptor‐Mediated Stimulation of NK Cells

For the antibody‐mediated stimulation of NK receptors, NK cells were preincubated with an isotype control mAb or mAbs specific for NK receptors (10 µg mL^−1^) for 30 min on ice. Thereafter, NK cells were stimulated by crosslinking with 25 µg mL^−1^ goat anti‐mouse F(ab′)_2_ secondary antibody (Jackson ImmunoResearch) at 37 °C for the indicated times. For the stimulation of NK receptors mediated by a plate‐immobilized antibody, 96‐well EIA/RIA Stripwell plates (Costar) were coated overnight at 4 °C with mAbs specific for NK receptors (10 µg/mL). Then, NK cells were added into NK receptor mAb‐coated plates for the indicated times. Beads used to study mouse NK cells were prepared by coating 10 µL of protein A/G beads (Pierce) with 3 µg of antibody in PBS containing 0.01% Tween‐20 for 1 h at 4 °C. After washing the beads thrice with PBS containing 0.01% Tween‐20 and 1% FBS, the beads and 2 × 10^6^ mouse NK cells separately chilled on ice were mixed, pelleted, and moved to water bath at 37 °C for the indicated times. Thereafter, the cells were moved onto ice, washed with DPBS, and lysed for further analysis.

### Europium‐Based Cytotoxicity Assay

To assess target cell lysis by NK cells, 721.221 and P815 cells were loaded with 40 µM BATDA reagent (Perkin Elmer) for 30 min at 37 °C. P815 cells were resuspended at 1 × 10^6^ cells mL^−1^ and incubated for 30 min at room temperature with mAbs (10 µg mL^−1^) to NKG2D and/or 2B4 in the medium containing 1 mM sulfinpyrazone (Sigma). The cells were then washed and incubated with NK cells in the presence of sulfinpyrazone for 2 h at 37 °C. The plates were mixed briefly and centrifuged at 1,400 rpm for 5 min. The supernatant (20 µL) was incubated with 200 µL of 20% europium solution (Perkin Elmer) in 0.3 M acetic acid for 5 min, and target cell lysis was detected using VICTOR ×4 multi‐label plate leader (Perkin Elmer).

### Measurement of Granzyme B and Cytokine Secretion by Enzyme‐Linked Immunosorbent Assay (ELISA)

Granzyme B (BioLegend), IFN‐γ (Pierce), and MIP‐1α (R&D Systems) secreted from NK cells after stimulation with 721.221 cells or beads coated with mAbs specific to NKG2D and/or 2B4 were measured by ELISA as described.^[^ ^54^ ^]^

### Flow Cytometric Analysis of Ca^2+^ Mobilization

Intracellular Ca^2+^ mobilization was assessed by flow cytometry after the labeling of cells with Fluo‐4 AM (Invitrogen) as described.^[^ ^51^ ^]^ NKL cells were labeled for 30 min at 30 °C with dye loading buffer (HBSS with 1% FBS, 4 µg/mL Fluo‐4 AM, and 4 mM probenecid). The cells were washed twice, resuspended in HBSS with 1% FBS, and incubated with mAbs (10 µg mL^−1^) specific for NKG2D and 2B4 for 30 min on ice. Cells were resuspended in HBSS with 1% FBS, warmed for 5 min at 37 °C in a water bath, and placed on a flow cytometer. After 30 s of data acquisition, 4 µg of crosslinking goat anti‐mouse F(ab′)2 was added and events were acquired for a further 5 min. Data were analyzed with FlowJo software (Tree Star).

### Western Blot Analysis

Cell lysates of purified mouse NK cells or human NKL cells were subject to immunoblotting for the indicated proteins, as described.^[^ ^54^ ^]^ Equal amounts of protein for each sample were resolved on 4%–20% Mini‐PROTEIN TGX precast gel (BioRad; 4 561 095) and subsequently transferred onto PVDF membranes (Millipore) in transfer buffer (25 mM Tris, 192 mM glycine, 20% (v/v) methanol). The membranes were blocked with 5% BSA or skim milk in TBS‐T (Tris‐buffered saline containing 0.1% Tween‐20) for 1 h and subsequently incubated with primary Abs and then with the HRP‐conjugated secondary Abs. Protein bands were visualized using SuperSignal West Pico (Pierce), detected using a LAS‐4000 machine (Fujifilm), and quantified with ImageJ software (NIH). Antibodies for the detection of signaling molecules were obtained from the following sources: HPK1 (Cell Signaling; 1:1000 dilution; 4472), actin (BD Biosciences; 1:5000 dilution; C4), pS376‐SLP‐76 (Cell Signaling; 1:1000 dilution; 13 177), SLP‐76 (Cell Signaling; 1:1000 dilution; 4958), pY174 Vav1 (Abcam; 1:1000 dilution; ab47282), pS536‐p65 (Cell Signaling; clone 93H1; 1:1000 dilution; 3033), pS473 Akt (Cell Signaling; 1:1000 dilution; 9271), and pY202/204 Erk1/2 (Cell Signaling; 1:1000 dilution; 9101).

### Plasma Cytokine Analysis

Plasma fractions were separated from the peripheral blood samples by density gradient centrifugation (Lymphocyte Separation Medium; MP Biomedicals), aliquoted, and stored at −80 °C until analysis. Plasma cytokines were analyzed as previously described.^[^ ^18^, ^23^ ^]^ In brief, the plasma samples were thawed on ice and then centrifuged at 1000 × g for 2 min at 4 °C to obtain the supernatant. The levels of IL‐1β, TNF‐α, and MIP‐1α were quantified using DuoSet ELISA (R&D Systems) according to the manufacturer's instructions. For the measurement of TGF‐β1, the plasma samples were subjected to acid activation using 1 N HCl to activate latent TGF‐β1 to the immunoreactive form, followed by neutralization with 1.2 N NaOH/0.5 M HEPES. The levels of TGF‐β1 were measured using human TGF‐beta 1 DuoSet ELISA (R&D Systems; DY240; detection range 31.3–2000 pg mL^−1^) according to the manufacturer's instructions. Magnetic Luminex assays (R&D Systems) were used to analyze 7 different cytokines (IL‐6, IL‐8, IL‐10, IL‐12 p40, IL‐15, IL‐18, and CXCL1). Briefly, the plasma samples were incubated for 2 h with anticytokine antibody‐coated beads (Human premixed multi‐analyte kit; R&D Systems; RND‐LXSAHM‐07) according to the manufacturer's instructions. The median fluorescence intensity was determined using a Luminex instrument, and the plasma concentrations of each cytokine were calculated by comparison with a standard curve. The detection range for cytokines was 4.07–990 pg mL^−1^ (IL‐6), 3.91–950 pg mL^−1^ (IL‐8), 4.24–1030 pg mL^−1^ (IL‐10), 503–122340 pg mL^−1^ (IL‐12 p40), 5.76–1400 pg/mL (IL‐15), 16.5–4020 pg mL^−1^ (IL‐18), and 50.6–12290 pg mL^−1^ (CXCL1).

### QuantSeq 3′ mRNA Sequencing and Analysis

Splenocytes were excised from mice with melanoma lung metastasis, and then NK cells were purified by negative selection using an EasySep mouse NK cell isolation kit (STEMCELL Technologies). Thereafter, NK cells were placed immediately in an RNAlater solution (Invitrogen) for RNA extraction, and total RNA was isolated using Trizol (Invitrogen) according to the manufacturer's instructions. RNA quality was assessed by an Agilent 2100 bioanalyzer using the RNA 6000 Pico kit (Agilent Technologies). The experiment was performed on RNA pooled from 3 individual mice per group. Library construction using the QuantSeq 3′ mRNA‐Seq Library Prep Kit (Lexogen Inc.) and QuantSeq 3′ mRNA sequencing was performed by ebiogen (Seoul, Korea), a specialized RNA‐Seq company. QuantSeq 3′ mRNA sequencing was a method of sequencing the 3′ untranslated region of mRNA with poly(A) tails, which enables accurate and affordable gene expression measurement (https://www.nature.com/articles/nmeth.f.376). In brief, an oligo‐dT primer containing an Illumina‐compatible sequence at its 5′end was hybridized to the total RNA, and reverse transcription was performed. After degradation of the RNA template, second strand synthesis was initiated by a random primer containing an Illumina‐compatible linker sequence at its 5′end. The double‐stranded library was purified by using magnetic beads to remove all reaction components. The library was amplified to add the complete adapter sequences required for cluster generation. The finished library was purified from PCR components. The high‐throughput sequencing was performed as single‐end 75‐bp sequencing using NextSeq 500 (Illumina, Inc., USA) to obtain more than 20 million reads per sample. The sequencing depths for control tumor‐free and tumor samples were 0.826991397 and 0.592431172, respectively. For data analysis, the sequenced reads were aligned using Bowtie2.^[^ ^77^ ^]^ DEGs were determined based on counts from unique and multiple alignments using coverage in Bedtools.^[^ ^78^ ^]^ Then the raw read count data were processed based on the quantile normalization method using the EdgeR program from Bioconductor.^[^ ^79^ ^]^ Thereafter, transcriptomic analysis was carried out using DAVID (https://david.abcc.ncifcrf.gov/) for gene functional annotation, and GSEA (https://software.broadinstitute.org/gsea/index.jsp) and KEGG (https://www.genome.jp/kegg/mapper/) for function and pathway enrichment analyses. Hierarchical cluster analysis was performed, and clusters and heatmaps were visualized using MeV 4.9.0. The QuantSeq data of mouse NK cells in this study were available at the NCBI Gene Expression Omnibus (GEO) with the accession number GSE248264.

### scRNA‐Seq Datasets from the Public Database

The 9 public scRNA‐seq datasets were used in this study; 2 melanoma datasets^[^ ^80^ ^]^ were obtained from the GEO with GSE120575 and GSE72056. For the GSE120575 dataset, clinical response for immunotherapy was available. Ovarian cancer and lung cancer datasets were obtained from the ArrayExpress database under accession numbers E‐MTAB‐8107, E‐MTAB‐6149, and E‐MTAB‐6653.^[^ ^81^ ^]^ The gastric cancer dataset was obtained from https://dna‐discovery.stanford.edu.^[^ ^82^ ^]^ The skin squamous cell carcinoma dataset was obtained from GSE144236.^[^ ^83^ ^]^ The pancreatic cancer dataset was obtained from Genome Sequence Archive (GSA): CRA001160 under project PRJCA001063.^[^ ^84^ ^]^ Two colorectal cancer datasets^[^ ^81^, ^85^ ^]^ were obtained from the GEO with GSE132465 and GSE144735. scRNA‐seq count matrix data with the NormalizeData function with LogNormalize method using Seurat R package v4.1.1 was normalized.^[^ ^86^ ^]^ Unique molecular identifiers (UMIs) of less than 401, expressed genes bigger than 6000 and smaller than 200, and more than 25% of reads mapping to mitochondrial RNA were filtered.

### Gene Set Enrichment and Pathway Analysis for scRNA‐Seq Data

GSEA (v4.1.0)^[^ ^87^ ^]^ or GSEAplot v0.1.0 R package^[^ ^88^ ^]^ was used to identify which gene sets were enriched compared to each other in 2 cell subtypes. Hallmark gene sets from the Molecular Signatures Database (MsigDB v7.2) or KEGG for pathway analysis were used. To assess the gene set enrichment scores of individual cells, gene set variation analysis (GSVA) scores were also calculated from the normalized gene expression data using GSVA v1.24.2.^[^ ^89^ ^]^

### Cell Type Annotation and Exhaustion Score for scRNA‐Seq Data

For the melanoma data (GSE72056), cell type annotation information including NK cell type was also available and this pre‐existing information for NK cell type was used. For other datasets, the SingleR package (v1.8.1)^[^ ^90^ ^]^ to annotate cell types including NK cells was used. The exhaustion score was measured using the mean expression of genes including *LAG3*, *PDCD1*, *HAVCR2*, and *TIGIT*.

### Trajectory Analysis in scRNA‐seq

Single‐cell trajectories were constructed in datasets for NK cells with *MAP4K1* expression and NK cells with no *MAP4K1* expression. Monocle2 (v2.22.0)^[^ ^91^ ^]^ was used to calculate pseudotime in each cell. The trajectory was constructed according to the unsupervised analysis method with selecting genes (mean_expression ≥ 0.1 and num_cells_expressed > 10). The dimensionality of each dataset was reduced using the “DDRTree” algorithm and ordered by the pseudotime. To identify the trajectory in each dataset with *MAP4K1* expression and exhaustion scores according to the pseudotime, the “plot_cell_trajectory” and “plot_genes_in_pseudotime” functions were used.

### NK Cell Signature Scoring and TCGA Bulk RNA Sequencing Data with Clinical Information

The 20‐gene NK cell signature was used for scoring the NK cell signature in bulk tissue RNA sequencing data.^[^ ^58^ ^]^ To calculate the TGF‐β upregulated signature score, the gene set of HALLMARK_TGF_BETA_SIGNALING from the molecular signature database was used. The scoring for these gene sets was performed using the *singscore* R package v1.20.0. To investigate the significance of signature scores in cancer tissue, upper quartile normalized gene expression data (illuminahiseq_rnaseqv2‐RSEM_gene_normalized) and corresponding clinical data for 8469 primary cancer tissues including 76 patients with primary cutaneous melanoma were downloaded (https://gdac.broadinstitute.org/). In addition, the normalized RNA sequencing expression data for 365 patients with metastatic cutaneous melanoma including clinical information were also downloaded (https://gdac.broadinstitute.org). Disease‐specific survival data was used for the survival analysis.

### Statistical Analysis

Experimental data were analyzed with GraphPad Prism software version 7 or 8 (GraphPad Software). The statistical analyses for scRNA‐seq and the TCGA datasets were performed using R version 4.2.1. Two groups were compared using Student's t‐test for parametric samples or Mann‐Whitney U‐test for non‐parametric samples. Correlation analysis of continuous variables was performed using Spearman′s correlation analysis. Parametric comparisons of more than 2 groups were analyzed by one‐way or 2‐way analysis of variance (ANOVA) followed by Dunnett's multiple comparison test. Non‐parametric comparisons of more than 2 matched groups were analyzed by repeated measure Friedman followed by Dunn's multiple comparison test. Patient clinical and demographic characteristics were analyzed with the SPSS (version 28.0; SPSS). The expression differences were assessed using the Wilcoxon rank‐sum test for 2 groups or the Kruskal‐Wallis test for more than 2 groups. Multivariate analysis was performed using either multivariate logistic regression analysis or Cox regression analysis. Univariate survival analysis was conducted using the log‐rank test and the survival curves were visualized with Kaplan‐Meier plots. All statistical analyses were conducted using two‐tailed tests. Statistical significance was defined as a *p*‐value < 0.05, and the exact *p*‐values were indicated in the figures along with the sample size (n). The *p*‐values less than 0.0001 were presented as *p *= 0.0001. Data were expressed as mean ± SD for in vitro studies or mean ± SEM for in vivo mouse studies. A reasonable sample size was chosen to achieve statistical significance and to ensure adequate reproducibility of the results, which were based on the previous studies including ours.^[^ ^25^, ^50^, ^92^ ^]^ All data points were shown for the results with mouse and patient studies, and no animal and patient data were excluded. Blinding was performed for in vivo mouse studies. All the experiments have been reproduced 2 to 3 independent times with comparable results.

## Conflict of Interest

The authors declare no competing financial interests.

## Author Contributions

W.S.C., H.‐J.K., C.O.S., and H.S.K. contributed equally to this work. W.S. Choi, H.‐J. Kwon, E. Yi, H. Lee, J.M. Kim, H.J. Park, and E.J. Choi were involved in data acquisition. W.S. Choi, H.‐J. Kwon, E. Yi, H. Lee, J.M. Kim, H.J. Park, and H.S. Kim were involved in data analysis and interpretation. M.E. Choi and C.H. Won helped design studies with patient analysis. Y.H. Sung helped design studies with conditional *MAP4K1* Tg and *MAP4K1* KO mice. C.O. Sung helped design studies in bulk RNA‐seq and scRNA‐seq analysis. C.H. Won, C.O. Sung, and H.S. Kim contributed to the conceptual design of the study and writing of the manuscript with an input from all co‐authors.

## Supporting information

Supporting Information

## Data Availability

The data that support the findings of this study are available from the corresponding author upon reasonable request.
