# Supplementary material for: HPK1 Dysregulation‐Associated NK Cell Dysfunction and Defective Expansion Promotes Metastatic Melanoma Progression
Source: Adv Sci (Weinh). 2024 Jun 3;11(29):2400920. doi: 10.1002/advs.202400920 (PMC11304315; doi:10.1002/advs.202400920)
Supplement: Supplementary file 1 — Supporting Information [file ADVS-11-2400920-s001.docx]

Supporting Information

**HPK1 dysregulation-associated NK cell dysfunction and defective expansion promotes metastatic melanoma progression**

Woo Seon Choi, Hyung-Joon Kwon, Eunbi Yi, Haeun Lee, Jung Min Kim, Hyo Jin Park, Eun Ji Choi, Myoung Eun Choi, Young Hoon Sung, Chong Hyun Won, Chang Ohk Sung*, and Hun Sik Kim*

**Supplementary figures**

**
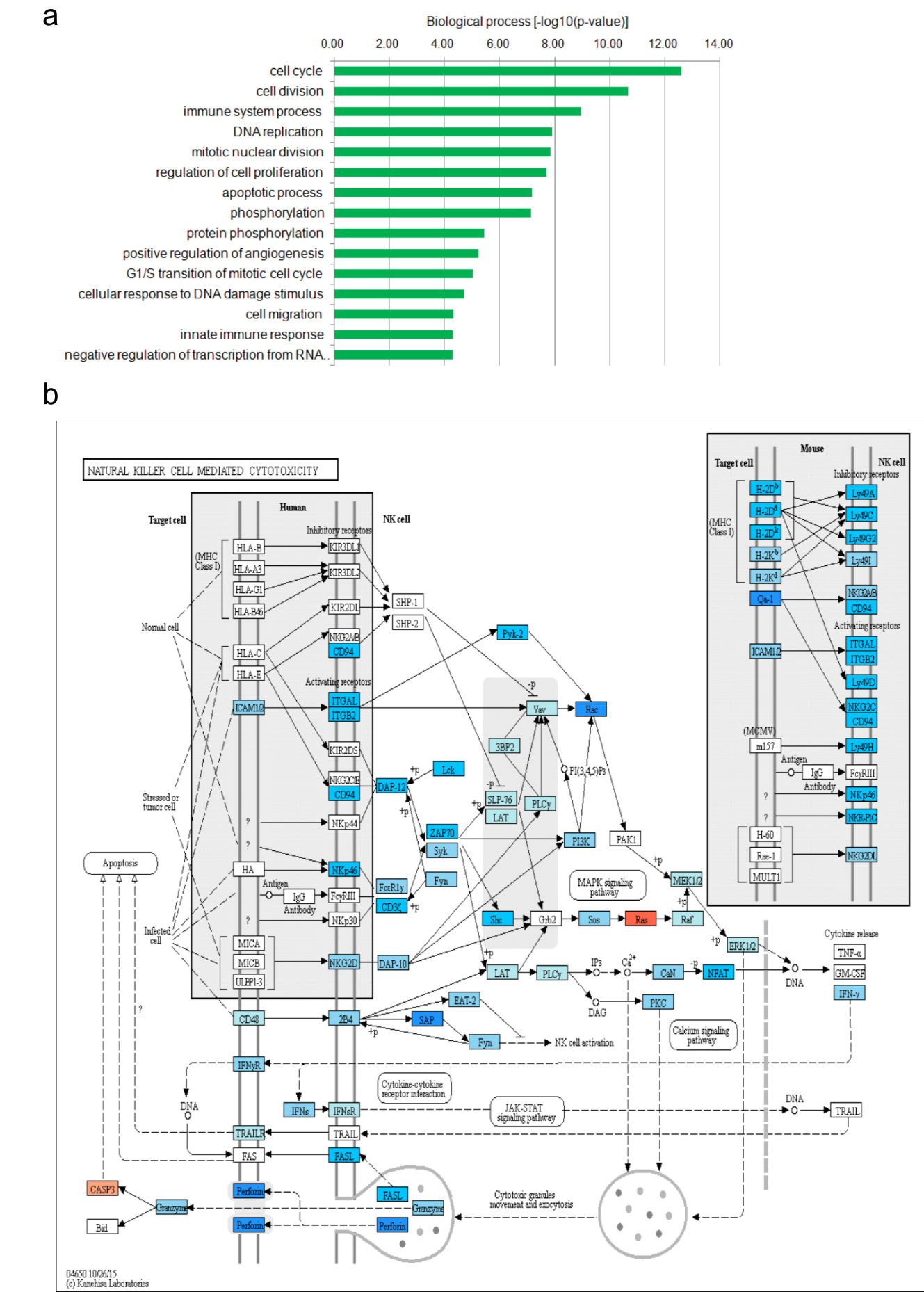
**

**Figure S1. Gene Ontology (GO) term and Kyoto Encyclopedia of Genes and Genomes (KEGG) analysis of splenic NK cells linked to metastatic progression.**

(**a**) GO term (biological process) enrichment analysis of genes that displayed > 2-fold change in expression in dysfunctional NK cells from the spleen of metastatic tumor-bearing mice compared with NK cells from the spleen of control mice. The analysis results were shown as a count of gene plots of the top 15 pathways.

# (b) KEGG map of NK cell-mediated cytotoxicity. Map of the "Natural Killer cell mediated cytotoxicity" pathway derived from KEGG (hsa04650). The genes that were upregulated and down-regulated are presented as red and blue color, respectively.


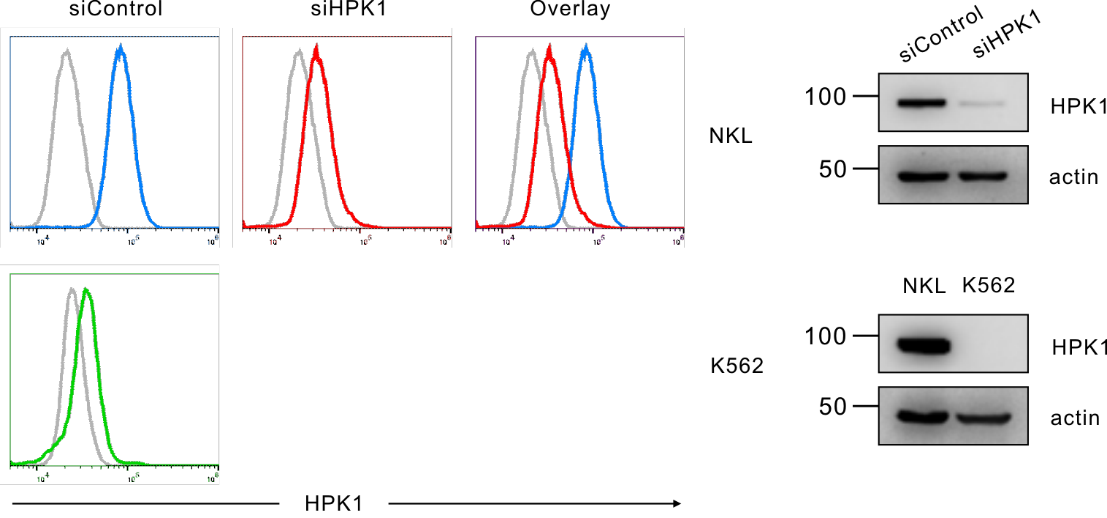


**Figure S2. Establishment of flow cytometry-based quantification of HPK1 expression in NK cells.**

NKL cells transfected with control siRNA or HPK1-specific siRNA were fixed, permeabilized, stained with normal rabbit IgG or Ab specific to HPK1, then Alexa Fluor 488-conjugated goat anti-rabbit IgG, and analyzed using flow cytometry. Representative flow cytometric analysis of HPK1 expression (left) in NKL cells transfected with control siRNA (blue), HPK1-specific siRNA (red), or in K562 cells (green). HPK1-deficient K562 cells were used as a control. The gray solid lines indicate normal rabbit IgG staining. Proper functioning of the flow cytometry-based measurement (left) was confirmed by immunoblot analysis (right). Data are representative of at least three independent experiments.


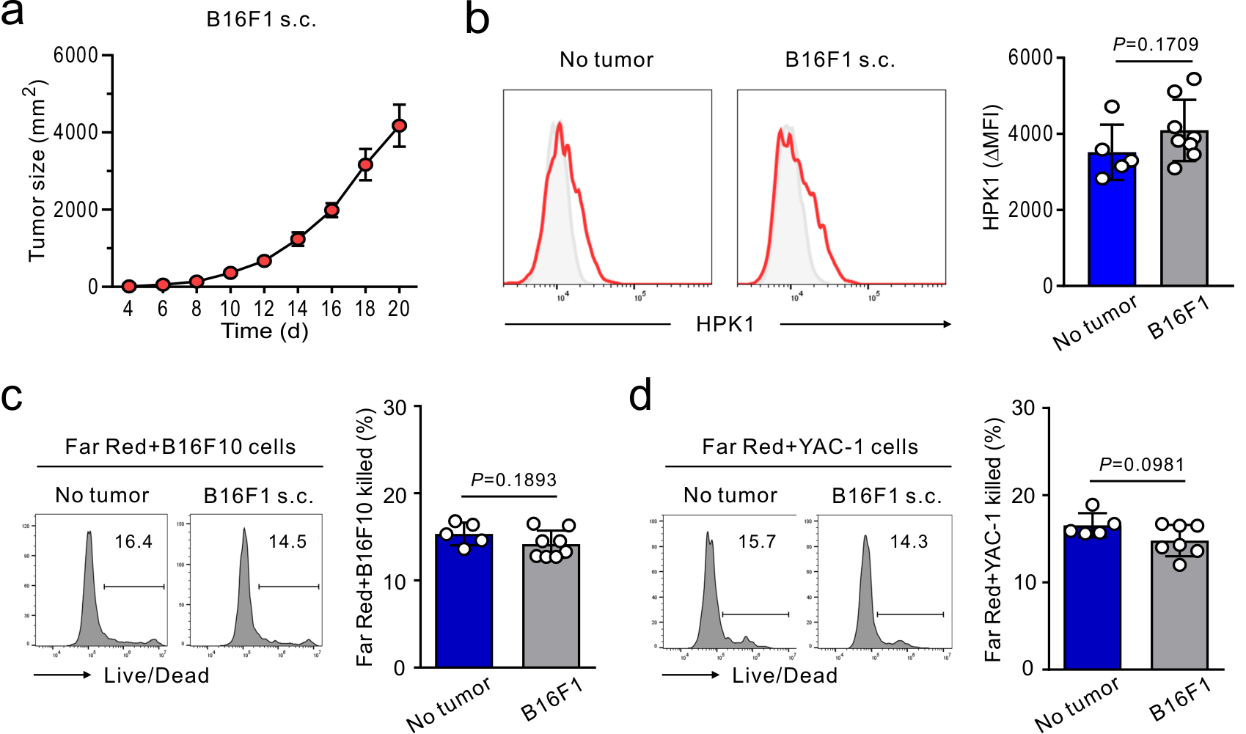


**Figure S3. Poorly metastatic B16F1 cells does not affect the cytotoxicity and the level of HPK1 expression in NK cells.**

(**a**) B16F1 cells were injected subcutaneously into shaved flank of C57BL/6 mice (n = 8 mice). Shown are the tumor sizes determined at the indicated time points.

(**b**) Flow cytometry analysis of HPK1 expression in splenic NK cells from control tumor-free (n = 5 mice) or B16F1-s.c. inoculated mice (n = 8 mice) on day 21. Representative result (left) and graph (right) showing the MFI of HPK1 expression relative to isotype control (ΔMFI).

(**c, d**) Flow cytometric analysis of NK cell cytotoxicity against B16F10 cells (c) and YAC-1 cells (d) in the spleen of control tumor-free and B16F1-s.c. inoculated mice. Shown are the representative result (left) and graph (right) of Live/Dead stain-positive dead cells.

Data represent the mean ± SD (b, c, and d); each dot represents an individual mouse. Data were analyzed using Mann-Whitney U-test (b) and two-tailed unpaired t-test (c, d); actual *P*-values are indicated.


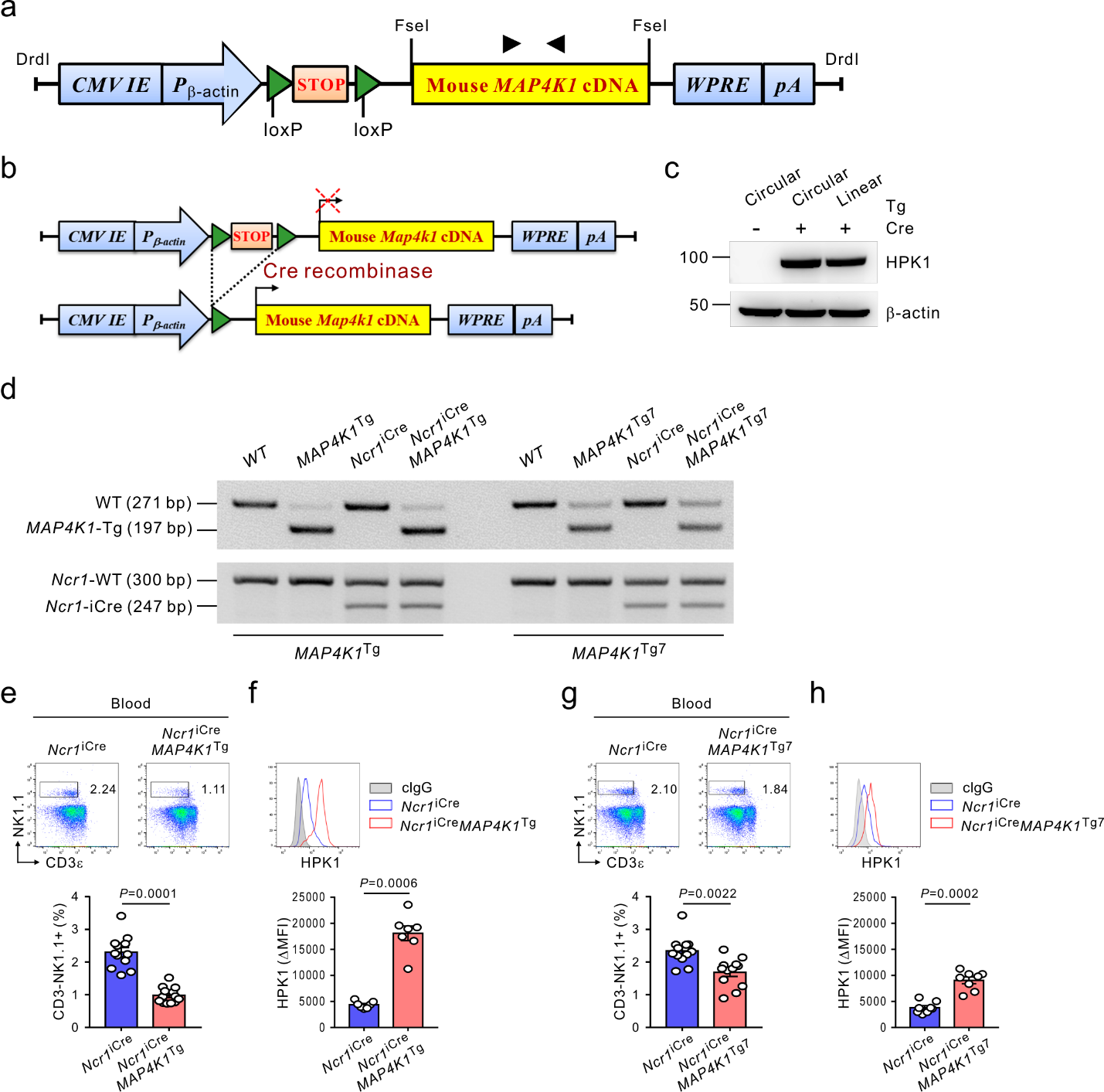


**Figure S4. Generation of conditional *MAP4K1* transgenic mice.**

(**a, b**) Schematic map of the conditional *MAP4K1* transgene construct (a). A full-length mouse *MAP4K1* cDNA was subcloned using FseI restriction enzyme sites downstream of the loxP-flanked 3×PolyA signals for the Cre recombinase-mediated expression of the transgene (b). The transgene was linearized using the DrdI restriction enzyme for the pronuclear microinjection of fertilized mouse eggs. Arrowheads denoted on the mouse *MAP4K1* cDNA indicated the PCR genotyping primer pair specific for both endogenous *MAP4K1* gene (271 bp) and the transgene (197 bp). CMV IE, CMV immediate early enhancer; P_β-actin_, chicken β-actin promoter and intron; triangles, loxP sequences; 3×PolyA, triple SV40 polyA signals; WPRE, woodchuck hepatitis virus posttranscriptional regulatory element; pA, bovine growth hormone polyadenylation signal.

(**c**) The Cre-mediated HPK1 (encoded by *MAP4K1*) protein expression from the transgene. The supercoil circular form or linearized transgene constructs were transfected into 293T cells with or without Cre recombinase construct, and immunoblot analysis was conducted using a HPK1-specific antibody. Tg, transgene. β-actin protein was used as a loading control.

(**d**) A representative genotyping data. An NK cell-specific Cre (*Ncr1*-iCre) transgenic mouse were crossed to our conditional *MAP4K1* transgenic mice (*MAP4K1*^Tg^ or *MAP4K1*^Tg7^), and progenies were genotyped using the genomic DNA samples obtained from their tail biopsies.

(**e, f**) Flow cytometric analysis of the frequencies of CD3^-^NK1.1^+^ NK cells (e) and HPK1 expression in the NK cells among CD45^+^ cell populations (f) in the blood of *Ncr1*^iCre^ or *Ncr1*^iCre^*MAP4K1*^Tg^ mice (E; n = 12-13 mice per group, F; n = 7 mice per group) is shown as the representative result (top) and graph (bottom).

(**g, h**) Flow cytometric analysis of the frequencies of CD3^-^NK1.1^+^ NK cells (g) and HPK1 expression in the NK cells (h) in the blood of *Ncr1*^iCre^ or *Ncr1*^iCre^*MAP4K1*^Tg7^ mice (g; n = 12 mice per group, h; n = 8 mice per group) is shown as the representative result (top) and graph (bottom).

Data were pooled from two or three independent experiments (e-h) and are expressed as mean ± SEM (e-h) and were analyzed using Mann-Whitney U-test (e-h); actual *P*-values are indicated.


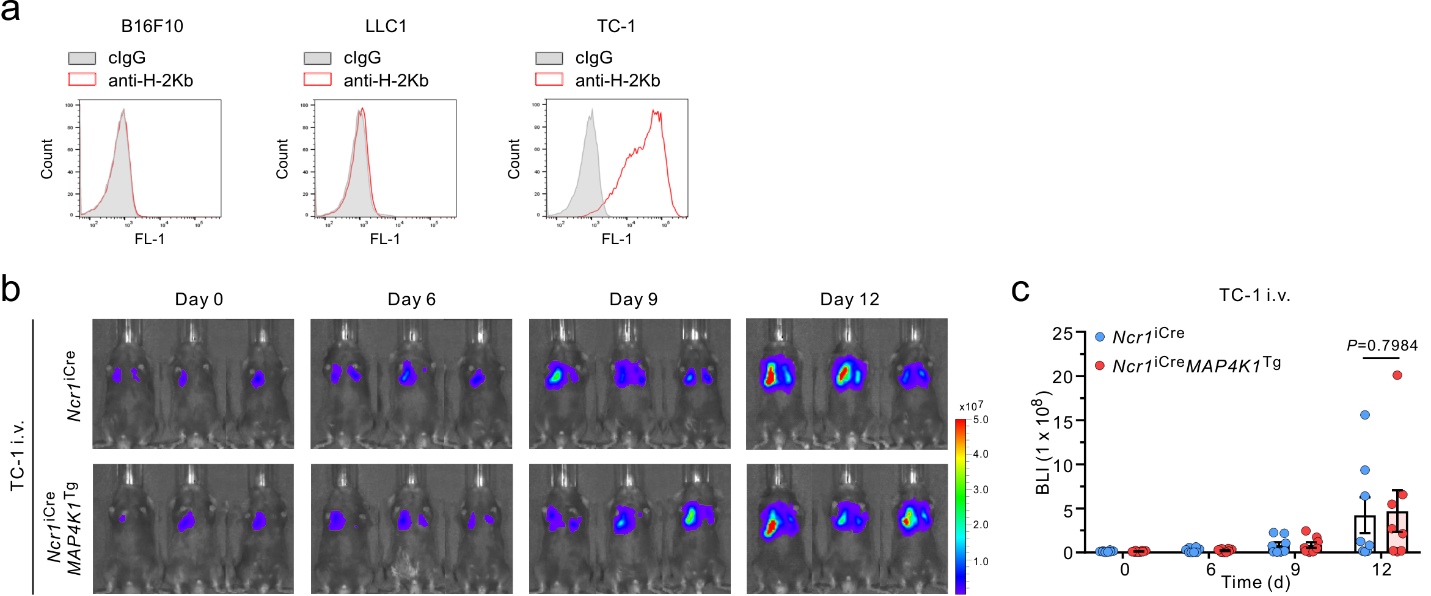


**Figure S5. Conditional HPK1 overexpression in NK cells does not affect experimental metastasis of TC-1 cells expressing high level of MHC class I surface expression.**

(**a**) Representative flow cytometry profile showing the expression levels of the MHC class I (H-2Kb) on B16F10 cells, LLC1 cell, and TC-1 cells (red solid line). Isotype control staining is shown as a shaded histogram. Data are representative of three independent experiments.

(**b, c**) Representative images (b) and quantification (c) of lung metastasis in *Ncr1*^iCre^ or *Ncr1*^iCre^*MAP4K1*^Tg^ mice (n = 8 mice per group) after i.v. administration of TC-1-Luc cells by luciferase-based BLI.

Data were pooled from two independent experiments and are expressed as mean ± SEM (c); each dot represents an individual mouse. Data were analyzed using Mann-Whitney U-test (c); actual *P*-values are indicated.

**
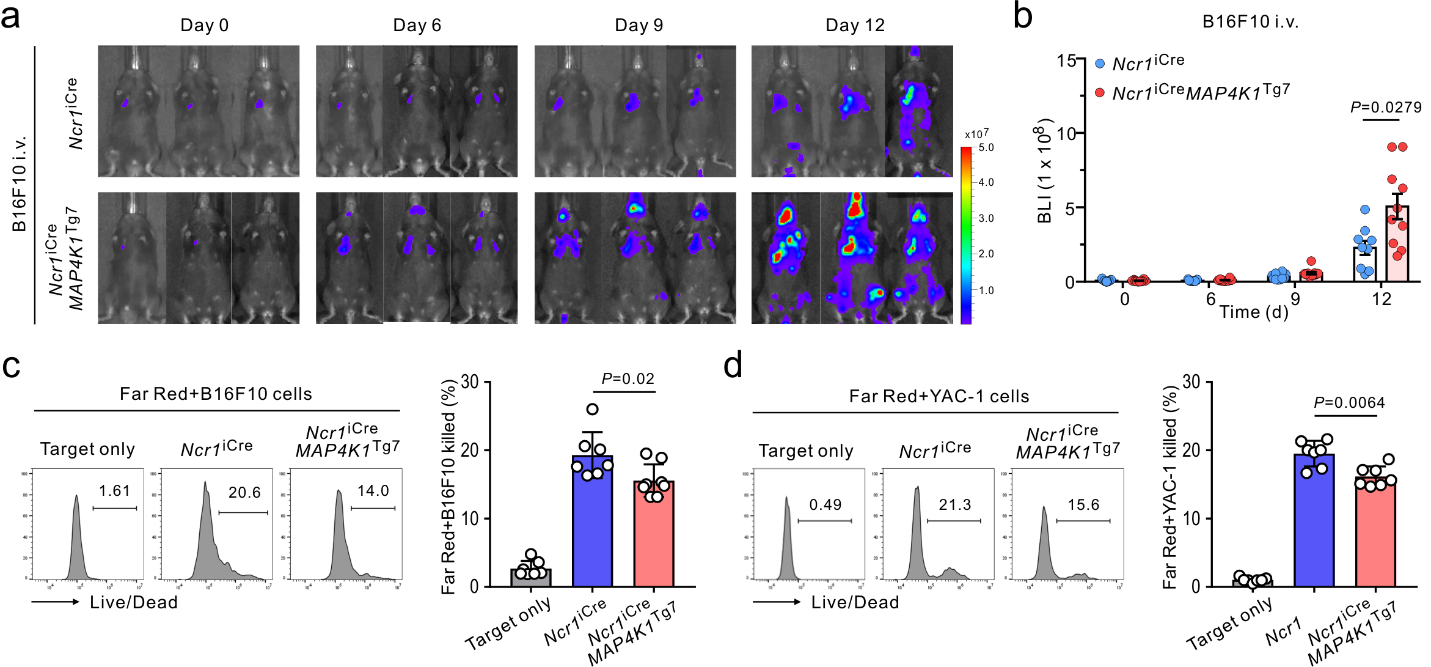
**

**Figure S6. HPK1 expression corresponds to the degree of NK cell dysfunction and metastatic susceptibility.**

(**a, b**) Representative images (a) and quantification (b) of metastasis to the lung and other organs in *Ncr1*^iCre^ (n = 9 mice) or *Ncr1*^iCre^*MAP4K1*^Tg7^ mice (n = 10 mice) after administration of B16F10-Luc2 cells by luciferase-based bioluminescence imaging (BLI).

(**c, d**) Flow cytometric analysis of NK cell cytotoxicity against B16F10 cells (c; n = 7-8 mice per group) and YAC-1 cells (d; n = 7 mice per group) in the spleen of *Ncr1*^iCre^ or *Ncr1*^iCre^*MAP4K1*^Tg7^ mice showing the representative result (left) and graph (right) of Live/Dead stain-positive dead cells.

Data were pooled from two independent experiments and are expressed as the mean ± SEM (b) or mean ± SD (c and d); each dot represents an individual mouse. Data were analyzed using Mann-Whitney U-test (b), two-tailed unpaired t-test (c and d); actual *P*-values are indicated.


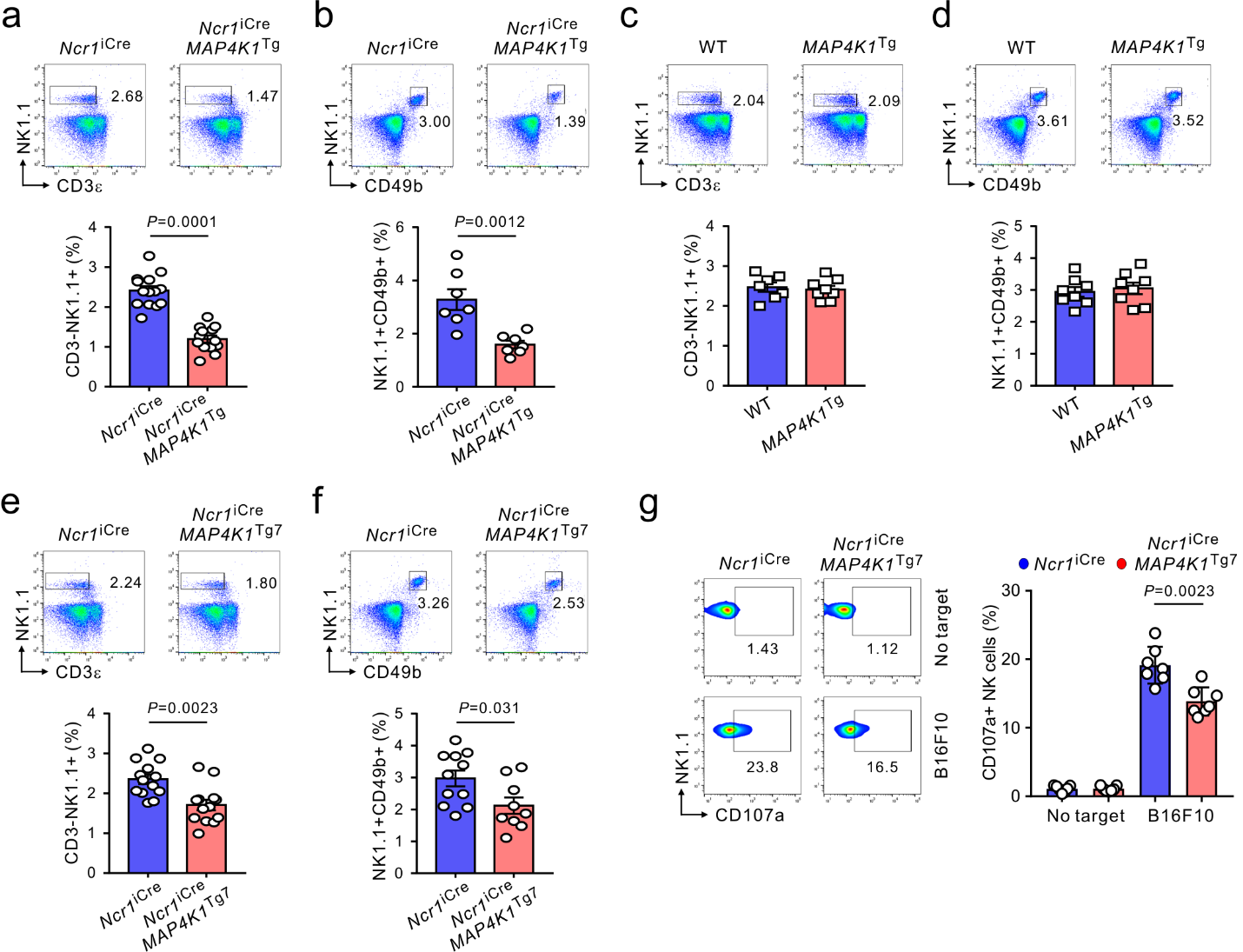


**Figure S7. HPK1 expression correlates with the degree of NK cell frequency.**

(**a, b**) Flow cytometric analysis of the frequencies of CD3^-^NK1.1^+^ NK cells (a) and NK1.1^+^CD49b^+^ NK cells (b) in the spleen of *Ncr1*^iCre^ or *Ncr1*^iCre^*MAP4K1*^Tg^ mice (a; n = 13 mice per group, b; n = 7 mice per group) is shown as the representative result (top) and graph (bottom).

(**c, d**) Flow cytometric analysis of the frequencies of CD3^-^NK1.1^+^ NK cells (c) and NK1.1^+^CD49b^+^ NK cells (d) in the spleen of WT or *MAP4K1*^Tg^ mice (c; n = 7-8 mice per group, d; n = 8 mice per group) is shown as the representative result (top) and graph (bottom).

(**e, f**) Flow cytometric analysis of the frequencies of CD3^-^NK1.1^+^ NK cells (e) and NK1.1^+^CD49b^+^ NK cells (f) in the spleen of *Ncr1*^iCre^ or *Ncr1*^iCre^*MAP4K1*^Tg7^ mice (e; n = 13 mice per group, f; n = 9-11 mice per group) is shown as the representative result (top) and graph (bottom).

(**g**) Degranulation assay of splenic NK cells against B16F10 cells (n = 7 mice per group). Representative result (left) and graph (right) showing percent increase of CD107a^+^ NK cells.

Data were pooled from two or three independent experiments and are expressed as mean ± SEM (a-e) or mean ± SD (g); each dot represents an individual mouse. Data were analyzed using Mann-Whitney U-test (a-e) and two-tailed unpaired t-test (g); actual *P*-values are indicated.


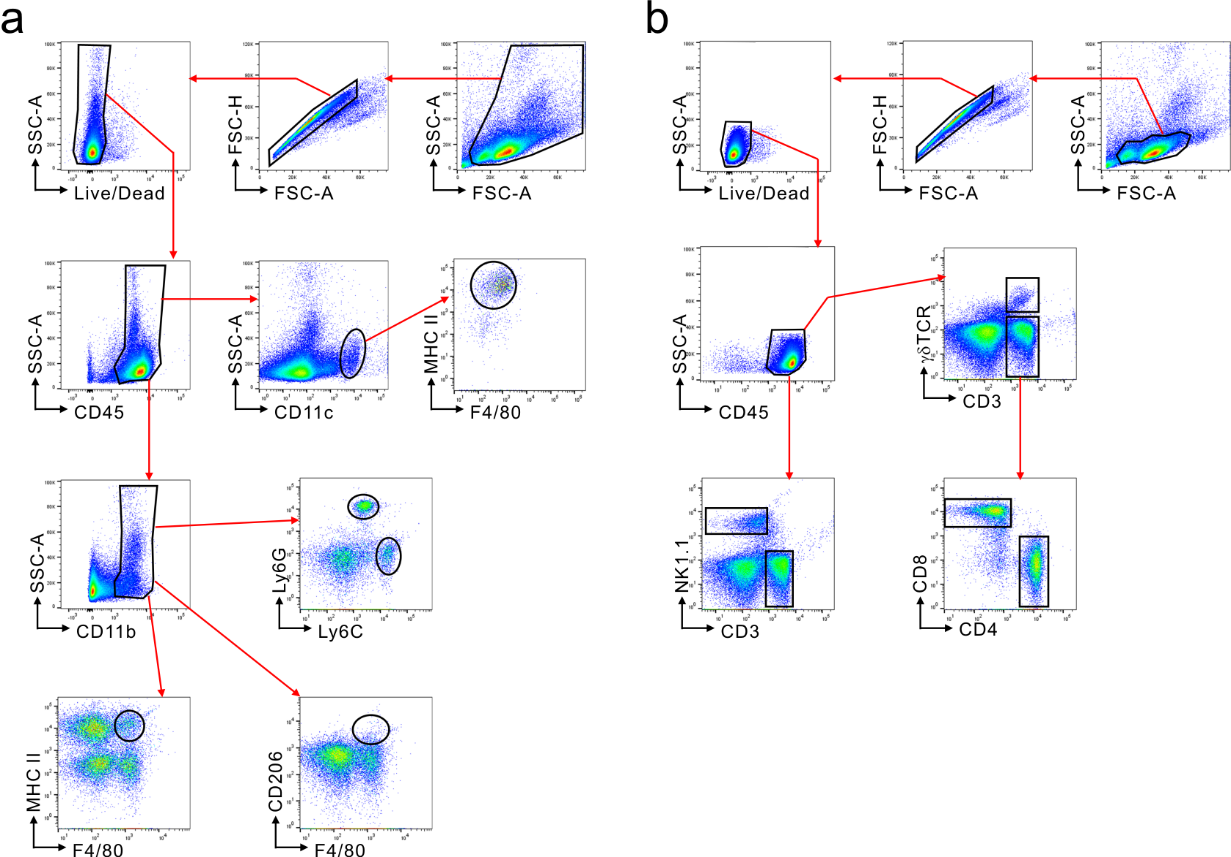


**Figure S8. Flow cytometric gating strategy.**

Profiles showing the gating strategy for identifying different myeloid cells and lymphocytes in various tissues and blood of this study.

(**a**) Gating strategy for myeloid panel: FSC vs. SSC, FSC-Area vs. FSC-Height, Live/Dead vs. SSC, CD45 vs. SSC, then CD11c vs. SSC and F4/80 vs. MHC II, or CD11b vs. SSC, F4/80 vs. MHC II, Ly6C vs. Ly6G, and F4/80 vs. CD206. Myeloid cell populations were identified as CD45^+^CD11b^+^Ly6G^+^Ly6C^int^ neutrophils, CD45^+^CD11b^+^Ly6G^-^Ly6C^+^ monocytes, CD45^+^CD11c^+^F4/80^-^MHCII^+^ dendritic cells, or CD45^+^CD11b^+^F4/80^+^MHCII^+^ M1-like and CD45^+^CD11b^+^F4/80^+^CD206^+^ M2-like macrophages.

(**b**) Gating strategy for lymphocyte panel: FSC vs. SSC, FSC-Area vs. FSC-Height, Live/Dead vs. SSC, CD45 vs. SSC, then CD3 vs. NK1.1 and CD11b vs. CD27, or CD3 vs. γδTCR and CD4 vs. CD8. Lymphocyte cell populations were identified as CD45^+^CD3^-^NK1.1^+^ NK cells, CD45^+^CD3^+^NK1.1^-^ T cells, CD45^+^CD3^+^γδ T cells, CD45^+^CD3^+^CD8^+^ T cells, and CD45^+^CD3^+^CD4^+^ T cells.


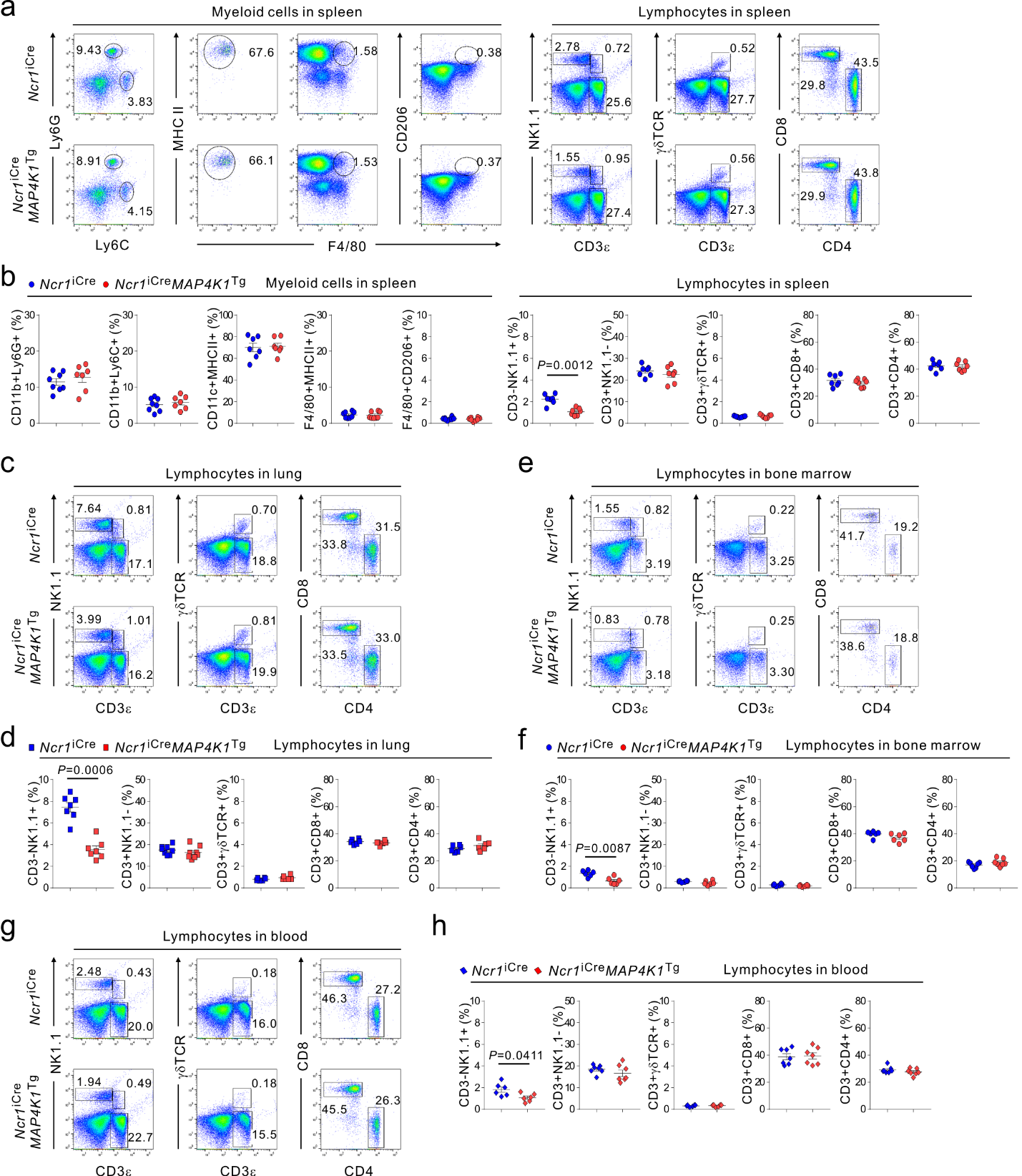


**Figure S9. Effect of HPK1 overexpression in NK cells on diverse immune cell populations.**

(**a, b**) Representative (a) and quantitative (b) flow cytometric analysis of different myeloid cells (CD11b^+^Ly6G^+^ neutrophils, CD11b^+^Ly6C^+^ monocytes, CD11c^+^MHCII^+^ dendritic cells, and F4/80^+^MHCII^+^ M1-like and F4/80^+^CD206^+^ M2-like macrophages) and lymphocytes (CD3^-^NK1.1^+^ NK cells, CD3^+^γδ T cells, CD8^+^ T cells, and CD4^+^ T cells) among CD45^+^ cell populations in the spleens of control *Ncr1*^iCre^ and *Ncr1*^iCre^*MAP4K1*^Tg^ mice (n = 7-8 mice per group).

(**c, d**) Representative (c) and quantitative (d) flow cytometric analysis of different lymphocytes among CD45^+^ cell populations in the lungs of control *Ncr1*^iCre^ and *Ncr1*^iCre^*MAP4K1*^Tg^ mice (n = 7 mice per group).

(**e-h**) Representative (e, g) and quantitative (f, h) flow cytometric analysis of different lymphocytes among CD45^+^ cell populations in the bone marrows (e, f) and peripheral blood (g, h) of control *Ncr1*^iCre^ and *Ncr1*^iCre^*MAP4K1*^Tg^ mice (n = 6 mice per group).

Data were pooled from two independent experiments. Horizontal bars indicate the means and each dot represents an individual mouse. Data were analyzed using Mann-Whitney U-test (b, d, f, and h); actual *P*-values are indicated.

**
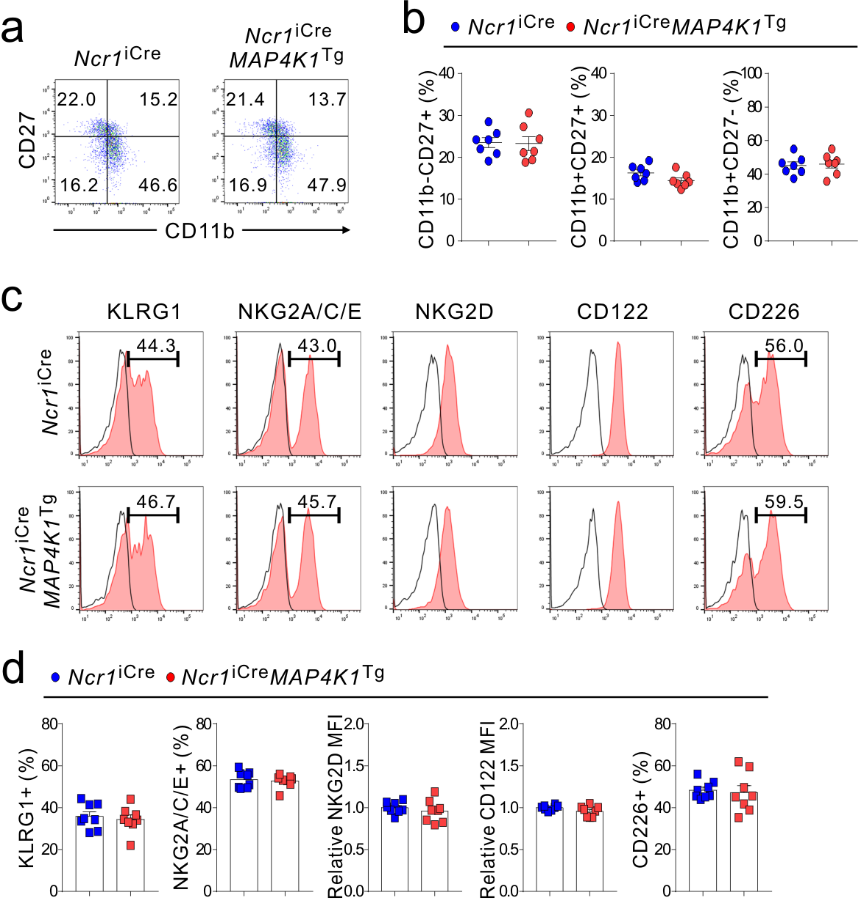
**

**Figure S10. HPK1 overexpression does not affect NK cell functional maturation.**

(**a, b**) Representative FACS profiles (a) and quantitative (b) flow cytometric analysis of CD11b and CD27 expression on CD3^-^NK1.1^+^ NK cells among CD45^+^ cell populations in the spleen of *Ncr1*^iCre^ or *Ncr1*^iCre^*MAP4K1*^Tg^ mice (n = 7 mice per group).

(**c, d**) Representative FACS profiles (c) and graph (d) showing the surface expression of KLRG1, NKG2A/C/E, NKG2D, CD122, and CD226 (red shaded histograms) on splenic NK cells from *Ncr1*^iCre^ or *Ncr1*^iCre^*MAP4K1*^Tg^ mice (n = 8 mice per group). The solid lines indicate the staining of the isotype control.

Horizontal bars indicate the means (b) and mean ± SEM (d); each dot represents an individual mouse.

**
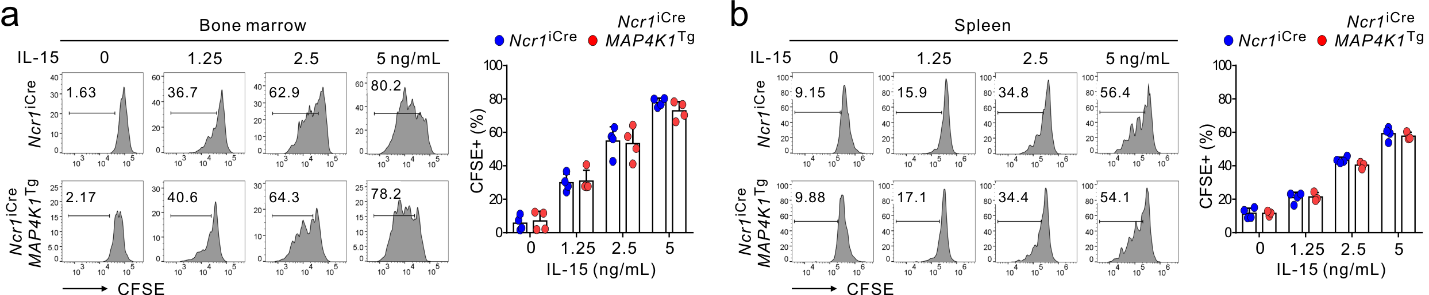
**

**Figure S11. Conditional HPK1 overexpression does not affect NK cell expansion via IL-15.**

(**a, b**) CFSE-labeled BM NK cells (a) and splenic NK cells (b) purified from *Ncr1*^iCre^ or *Ncr1*^iCre^*MAP4K1*^Tg^ mice (n = 4 mice per group) were cultured at the indicated concentrations of IL-15 for three days, followed by flow cytometric analysis of CFSE dilution. Shown is the representative result (left) and graph (right) of the percent increase of CFSE-diluted proliferating NK cells. Data represent the mean ± SD; each dot represents an individual mouse.


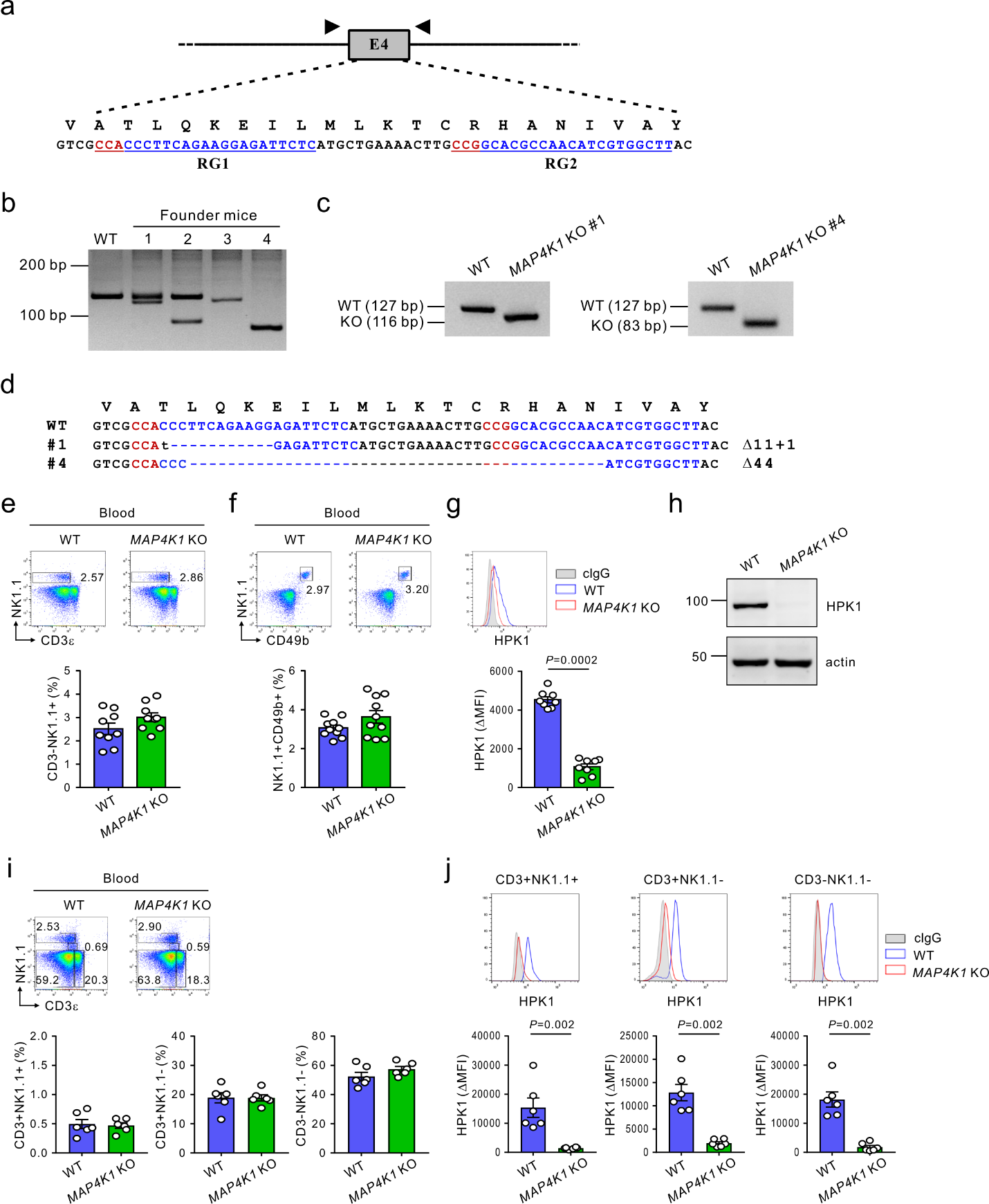


**Figure S12. Generation of *MAP4K1*-deficient mice.**

(**a**) The mouse *MAP4K1* gene targeting strategy using CRISPR-Cas9. Two sgRNAs specifically targeting the exon 4 of mouse *MAP4K1* gene were simultaneously used. The PCR genotyping primers are denoted as arrowheads. Target and protospacer-adjacent motif (PAM) sequences are denoted with blue and red colors, respectively.

(**b, c**) Founder mouse screen (b) and representative genotyping results of the WT and established *MAP4K1*-deficient mouse lines (c). Mutant founders with insertion and deletion (indel) mutations were identified by the agarose gel electrophoresis. WT, wild-type. Data are representative of at least three independent experiments.

(**d**) Sequence analyses of mutant alleles. ‘-’ denotes deleted nucleotides. Sequences in lower case represent nucleotide insertions.

(**e, f**) Flow cytometric analysis of the frequencies of CD3^-^NK1.1^+^ NK cells (e) and NK1.1^+^CD49b^+^ NK cells (f) among CD45^+^ cell populations in the blood of WT or *MAP4K1* KO mice (e; n = 9 mice per group, f; n = 10 mice per group) is shown as the representative result (top) and graph (bottom).

(**g**) Flow cytometric analysis of HPK1 expression in CD3^-^NK1.1^+^ NK cells in the blood of WT or *MAP4K1* KO mice (n = 8 mice per group) relative to isotype control (ΔMFI) is shown as the representative result (top) and graph (bottom).

(**h**) Western blot analysis of HPK1 expression in splenic NK cells from WT or *MAP4K1* KO mice. Cell lysates were immunoblotted for HPK1 or actin as a loading control. Data are representative of three independent experiments.

(**i**) Flow cytometric analysis of the frequencies of CD3^+^NK1.1^+^ cells, CD3^+^NK1.1^-^ cells, or CD3^-^NK1.1^-^ cells among CD45^+^ cell populations in the blood of WT or *MAP4K1* KO mice (n = 6 mice per group) is shown as the representative result (top) and graph (bottom).

(**j**) Flow cytometric analysis of HPK1 expression in CD3^+^NK1.1^+^ cells, CD3^+^NK1.1^-^ cells, or CD3^-^NK1.1^-^ cells in the blood of WT or *MAP4K1* KO mice (n = 6 mice per group) relative to isotype control (ΔMFI) is shown as the representative result (top) and graph (bottom).

Data were pooled from two independent experiments and are expressed as the mean ± SEM (e, f, g, i and j); each dot represents an individual mouse. Data were analyzed using Mann-Whitney U-test (g and j); actual *P*-values are indicated.


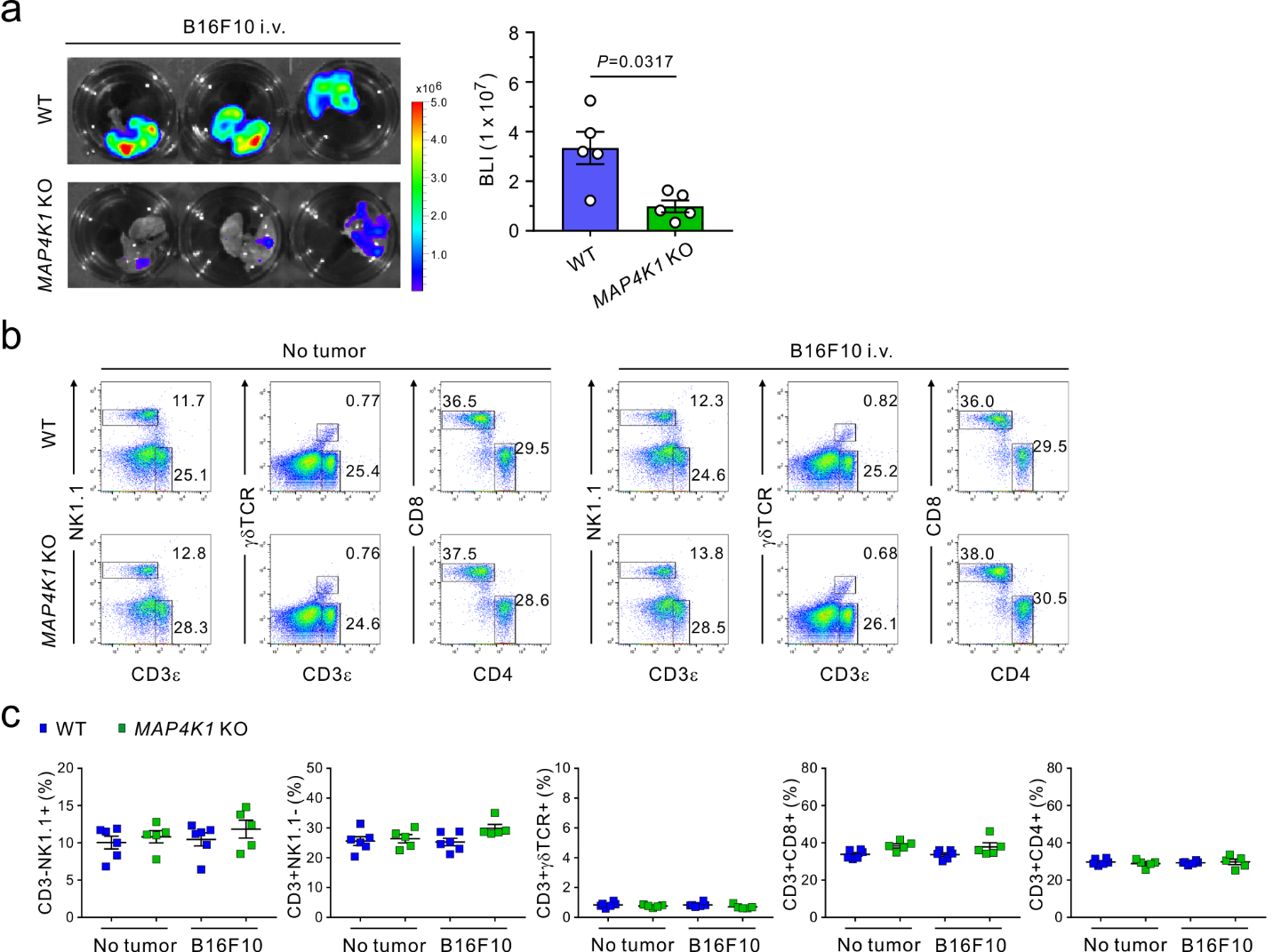


**Figure S13. HPK1 deficiency enhances anti-metastatic effect but not infiltration of NK cells in the metastasis-bearing lung.**

(**a**) WT or *MAP4K1* KO mice (n = 8 mice per group) were i.v. injected with B16F10-Luc2 cells and were euthanized at 1 day later for the measurement of BLI. Shown are the representative bioluminescence images of the lungs (left) and quantification of lung metastases by BLI (right) at 1 day post-challenge of B16F10-Luc2 cells.

(**b, c**) Representative (b) and quantitative (c) flow cytometric analysis of different lymphocytes (CD3^-^NK1.1^+^ NK cells, CD3^+^γδ T cells, CD8^+^ T cells, and CD4^+^ T cells) among CD45^+^ cell populations in the metastasis-bearing lungs of WT (n = 6) or *MAP4K1* KO mice (n = 5).

Data are expressed as mean ± SEM (a), and horizontal bars indicate the means (c); each dot represents an individual mouse. Data were analyzed using Mann-Whitney U-test (a); actual *P*-values are indicated.


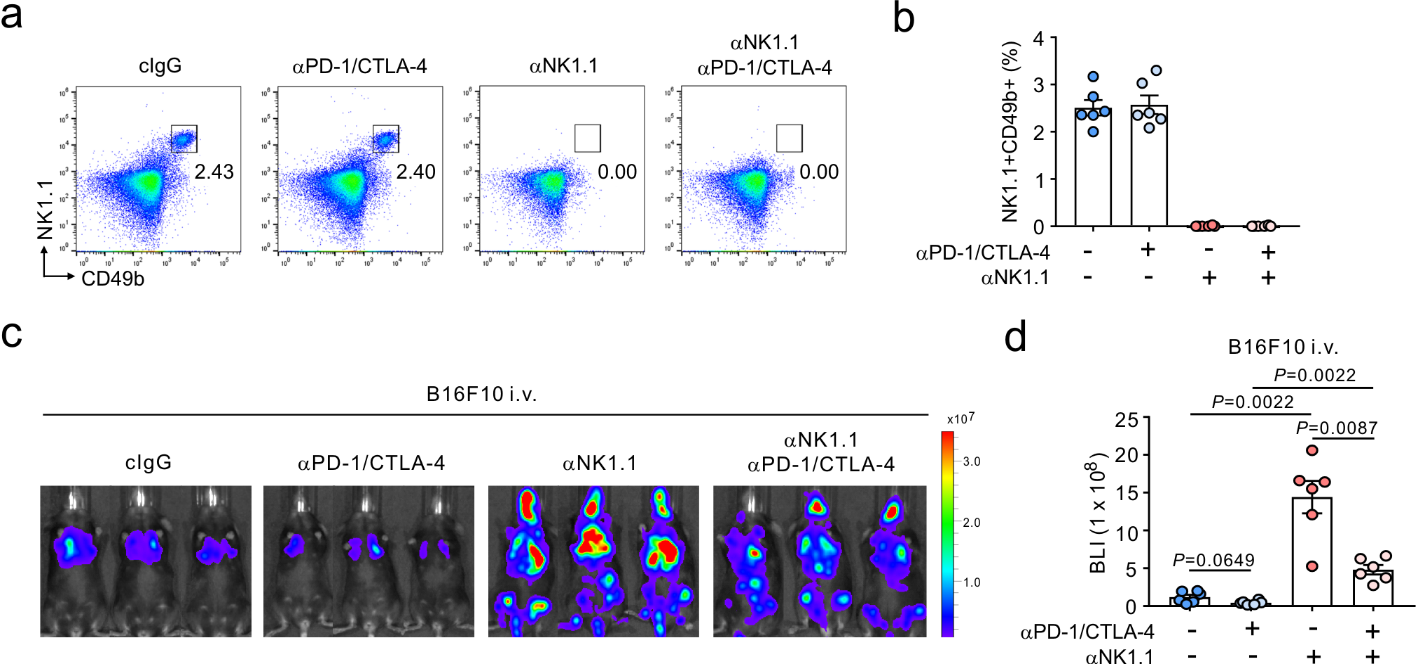


**Figure S14. Anti-metastatic effect of dual checkpoint blockade independently of NK cells.**

(**a, b**) C57BL/6 mice (n = 6 per group) were treated with i.p. injection of control IgG (150 μg) or anti-NK1.1 antibody (150 μg) before an i.v. injection of B16F10-Luc2 cells and twice a week treatment thereafter. Mice for dual checkpoint blockade were treated with either control IgG (500 μg) or anti-PD-1 and CTLA-4 (250 μg each) on days 0 and 3 post-injection of B16F10-Luc2 cells, respectively. After 12 days later, mice were euthanized and the frequencies of NK1.1^+^CD49b^+^ NK cells in the spleen of each group are shown as the representative result (a) and graph (b).

(**c, d**) Representative images (c) and quantification (d) of melanoma metastasis by BLI (day 12) showing the increased metastases to the lung and other organs of mice treated with anti-NK1.1 antibody, which was significantly attenuated by dual checkpoint blockade.

Data are expressed as mean ± SD (b) or mean ± SEM (d); each dot represents an individual mouse. Data were analyzed using Mann-Whitney U-test (d); actual *P*-values are indicated.


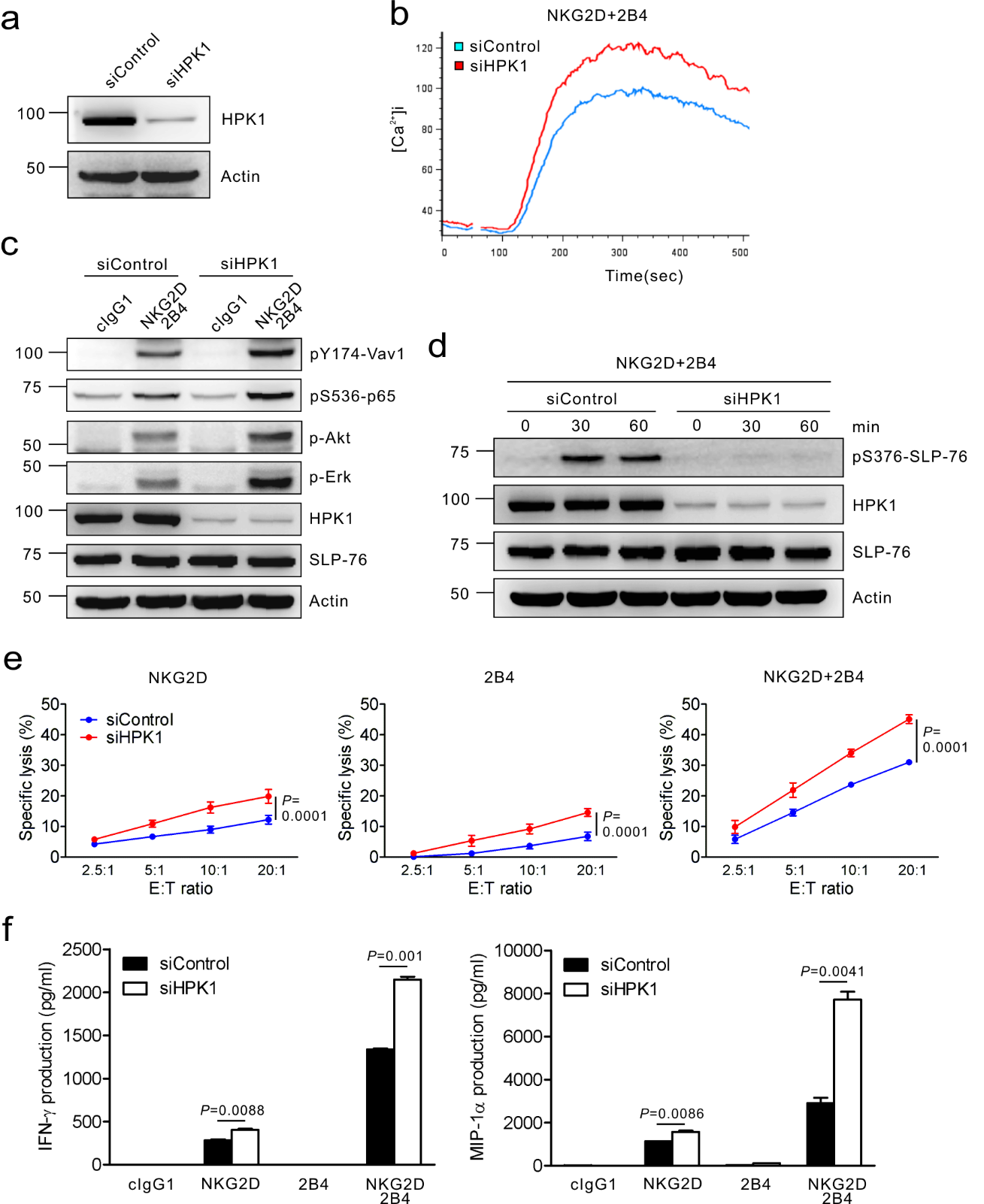


**Figure S15. HPK1 knockdown enhances human NK cell activation and effector function.**

(**a**) Cell lysates of NKL cells transfected with control siRNA or siRNA specific for HPK1 were immunoblotted for HPK1 and actin.

(**b**) NKL cells transfected with control siRNA or siRNA specific for HPK1 were loaded with Fluo-4 dye and then stimulated through NKG2D and 2B4 after the measurement of baseline Ca^2+^ flux for 30 s.

(**c**) NKL cells transfected with control siRNA or siRNA specific for HPK1 were stimulated through control IgG1 or NKG2D and 2B4 for 5 min. Cell lysates were immunoblotted for phospho-Vav1 at tyrosine 174 (pY174), phospho-NF-κB p65 at serine 536 (pS536), phospho-Akt at serine 473, phospho-Erk1 and 2, HPK1, SLP-76, or actin.

(**d**) NKL cells transfected with control siRNA or siRNA specific for HPK1 were stimulated through NKG2D and 2B4 for the indicated times. Lysates were immunoblotted for phospho-SLP-76 at serine 376 (pS376), HPK1, SLP-76, or actin.

(**e**) Lysis of P815 cells engaging NKG2D, 2B4, or both by NKL cells transfected with control siRNA or HPK1-specific siRNA at the indicated effector to target (E:T) cell ratio, as determined by europium assay (triplicate samples per group).

(**f**) NKL cells transfected with control siRNA or HPK1-specific siRNA were stimulated with NKG2D, 2B4, or both. After 8 h incubation, IFN-γ (left) and MIP-1α (right) released in the supernatant were measured by ELISA (triplicate samples per group).

Data represent the mean ± SD (e and f) and were analyzed using two-way ANOVA with Dunnett’s multiple comparison test (e) and two-tailed unpaired t-test (f); actual *P*-values are indicated. All data are representative of at least three independent experiments.


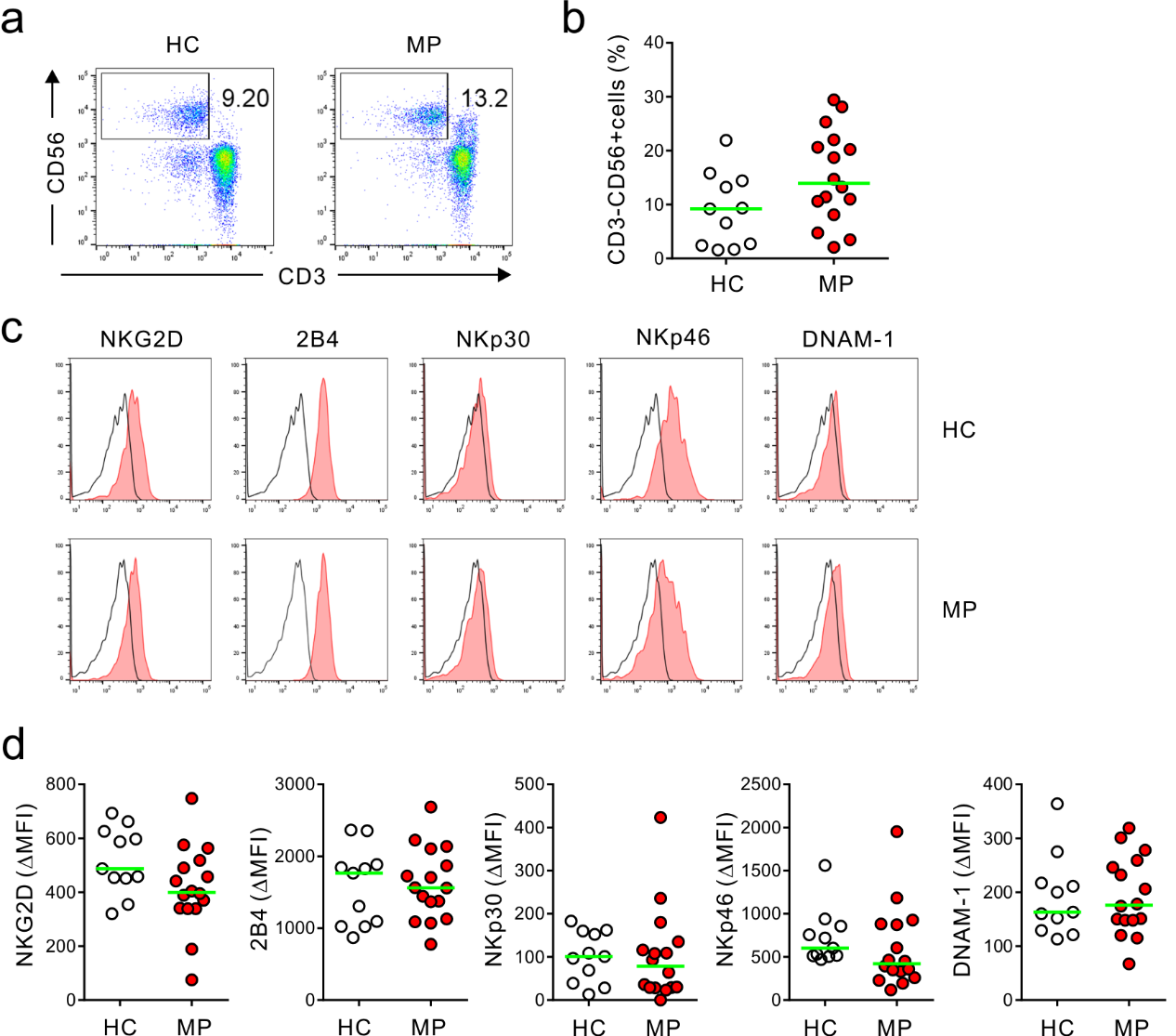


**Figure S16. The MP group has comparable NK cell frequencies and activating receptor levels to the HC group.**

(**a, b**) Representative FACS profiles (a) and graph (b) showing the frequency of CD3^-^CD56^+^ NK cells in the PBMCs from the HC group (n = 11) and MP group (n = 16).

(**c, d**) Representative FACS profiles (c) and graph (d) showing the expression of NKG2D, 2B4, NKp30, NKp46, and DNAM-1 (red shaded histograms) on gated NK cells in the HC group and MP group. The solid lines indicate the staining of the isotype control.

Data were pooled from five independent experiments. Horizontal bars (green) indicates the medians (b and d); each dot represents an individual donor.


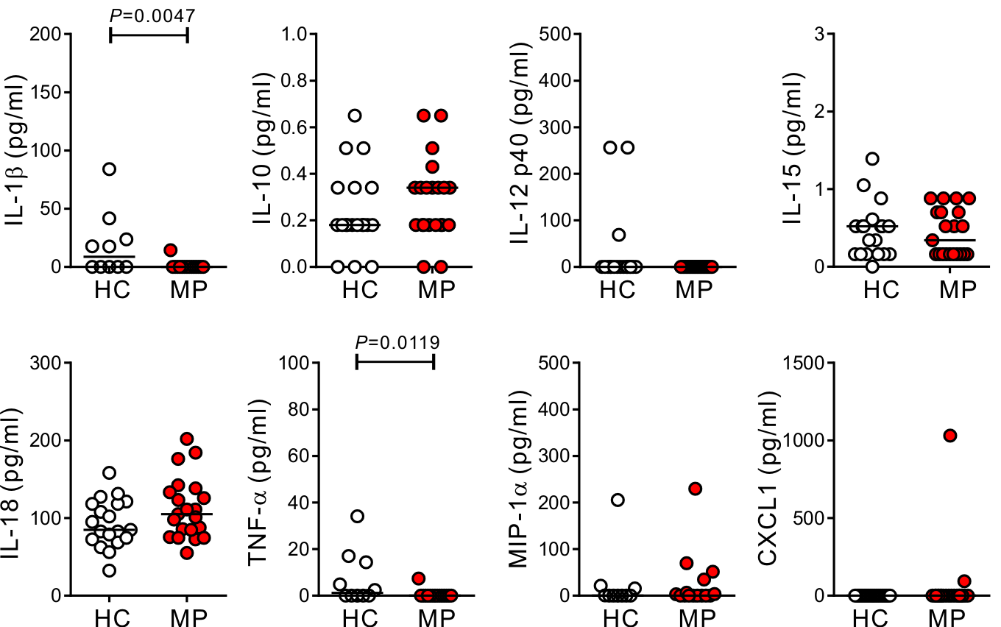


**Figure S17. Comparison of the levels of multiple cytokines and chemokines between the HC and MP group.**

The plasma levels of IL-1β, IL-10, IL-12 p40, IL-15, IL-18, TNF-α, MIP-1α, and CXCL1 were measured by Luminex multiplex assay or ELISA in the HC group (n = 10 for IL-1β, TNF-α, and MIP-1α; n = 19 for others) and MP group (n = 15 for IL-1β, TNF-α, and MIP-1α; n = 21 for others). Horizontal bars indicates the medians; each dot represents an individual donor. Data were analyzed using Mann-Whitney U-test; actual *P*-values are indicated.


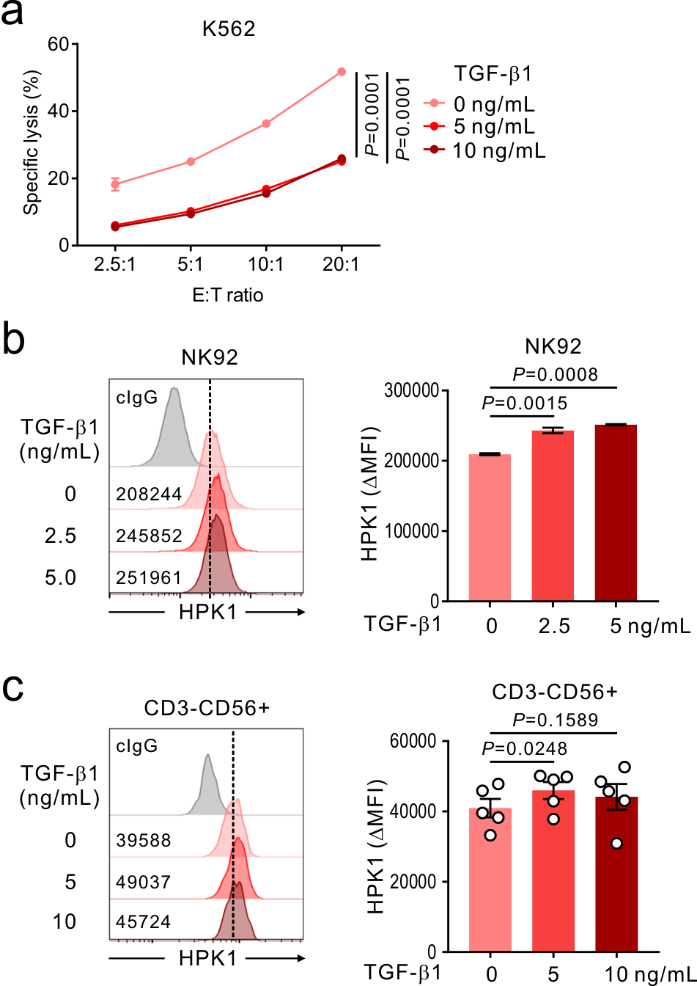


**Figure S18. TGF-β1 upregulates the expression of HPK1 in NK cells.**

(**a**) NK92 cells were pretreated with the indicated concentrations of TGF-β1 for 24 h. The cytotoxicity was then measured against K562 cells at the indicated effector to target (E:T) cell ratio using the europium assay (triplicate samples per group).

(**b**) NK92 cells were treated with the indicated concentrations of TGF-β1 for 6 h. Thereafter, HPK1 expression relative to isotype control (ΔMFI) in the NK92 cells was analyzed by flow cytometry. Representative result (left) and graph (right) are shown (duplicate samples per group).

(**c**) PBMCs were treated with the indicated concentrations of TGF-β1 for 24 h. The expression of HPK1 relative to isotype control (ΔMFI) in the NK cells was then analyzed by flow cytometry gated on CD3^-^CD56^+^ NK cells. Representative result (left) and graph (right) are shown.

Data represent the mean ± SD (a-c); each dot represents an individual donor (c). Data were analyzed using two-way ANOVA (a) or one-way ANOVA (b) with Dunnett’s multiple comparison test and repeated measure ANOVA Friedman with Dunn’s multiple comparison test (c); actual *P*-values are indicated. Data are representative of three independent experiments (a and b).


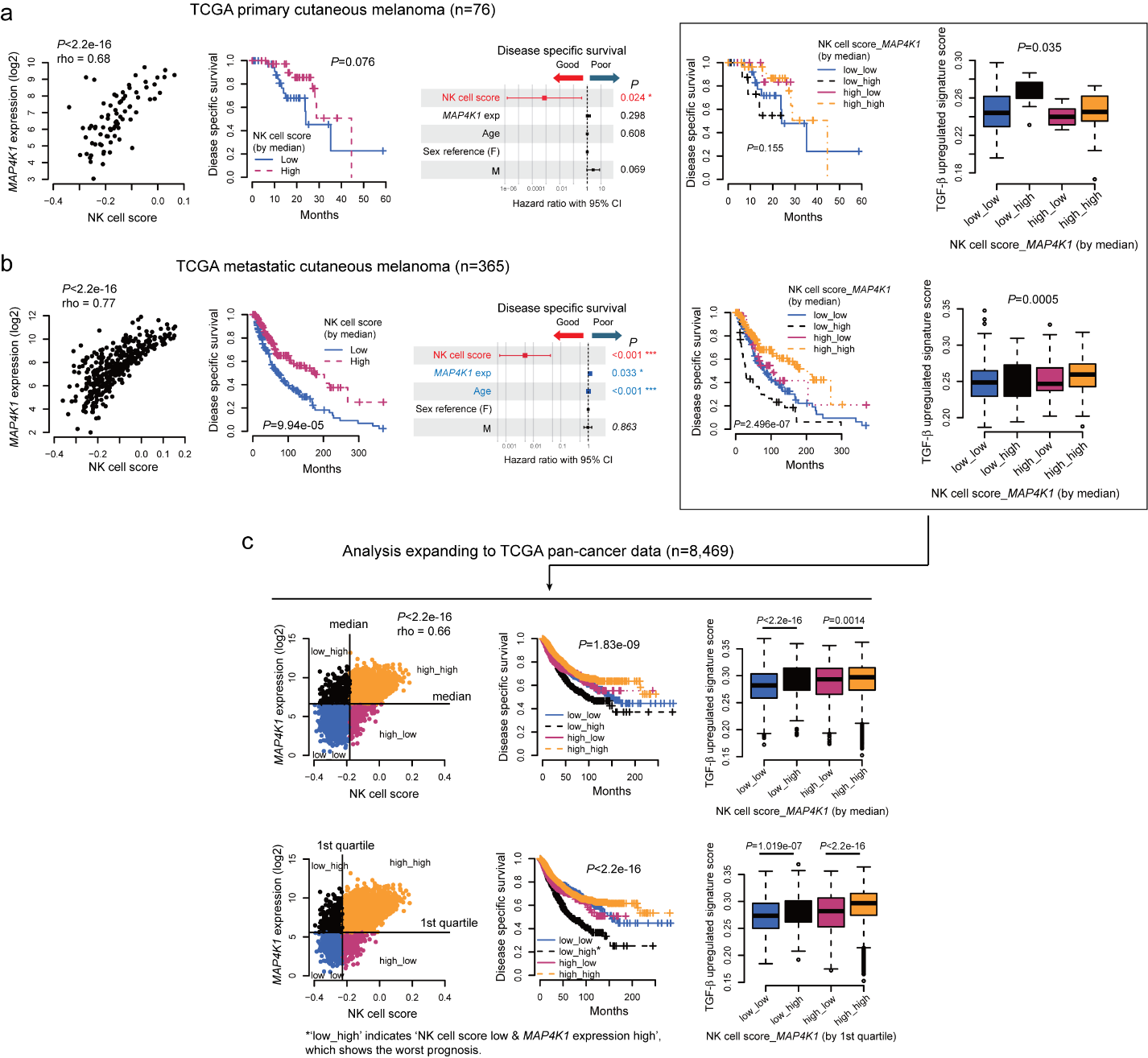


**Figure S19. NK cell signature score and *MAP4K1* expression in patients with malignant melanoma.**

(**a**) In 76 primary cutaneous melanoma patients from TCGA datasets, there was a significant correlation between NK score and *MAP4K1* expression (Spearman correlation test). Patients with high NK scores exhibited a favorable prognosis trend (Log-rank test), and when adjusted for *MAP4K1* expression, there was a significant favorable prognosis associated with NK score (Multivariate Cox regression analysis). When the patients were stratified into four groups based on the median values of NK score and *MAP4K1* expression, the NK score_low and *MAP4K1*_high group showed a trend towards poorer prognosis (Log-rank test). The group with high *MAP4K1* expression showed elevated TGF-β signaling compared to the group with low expression (Kruskal-Wallis test).

(**b**) When the same approach was applied to a larger sample of metastatic melanoma patients from TCGA datasets, the survival effect of *MAP4K1* in relation to TGF-β signaling became more significant. Specifically, *MAP4K1* expression was significantly associated with worse prognosis in contrast to the NK score (Multivariate Cox regression analysis), and the patients with NK score_low and *MAP4K1*_high exhibited the worst prognosis (Log-rank test).

(**c**) Applying the same approach to pan-cancer data from TCGA datasets further elucidated the worst prognosis associated with NK score_low and *MAP4K1*_high group (Log-rank test) and correlation of *MAP4K1* expression with TGF-β signaling. Actual *P*-values are indicated.


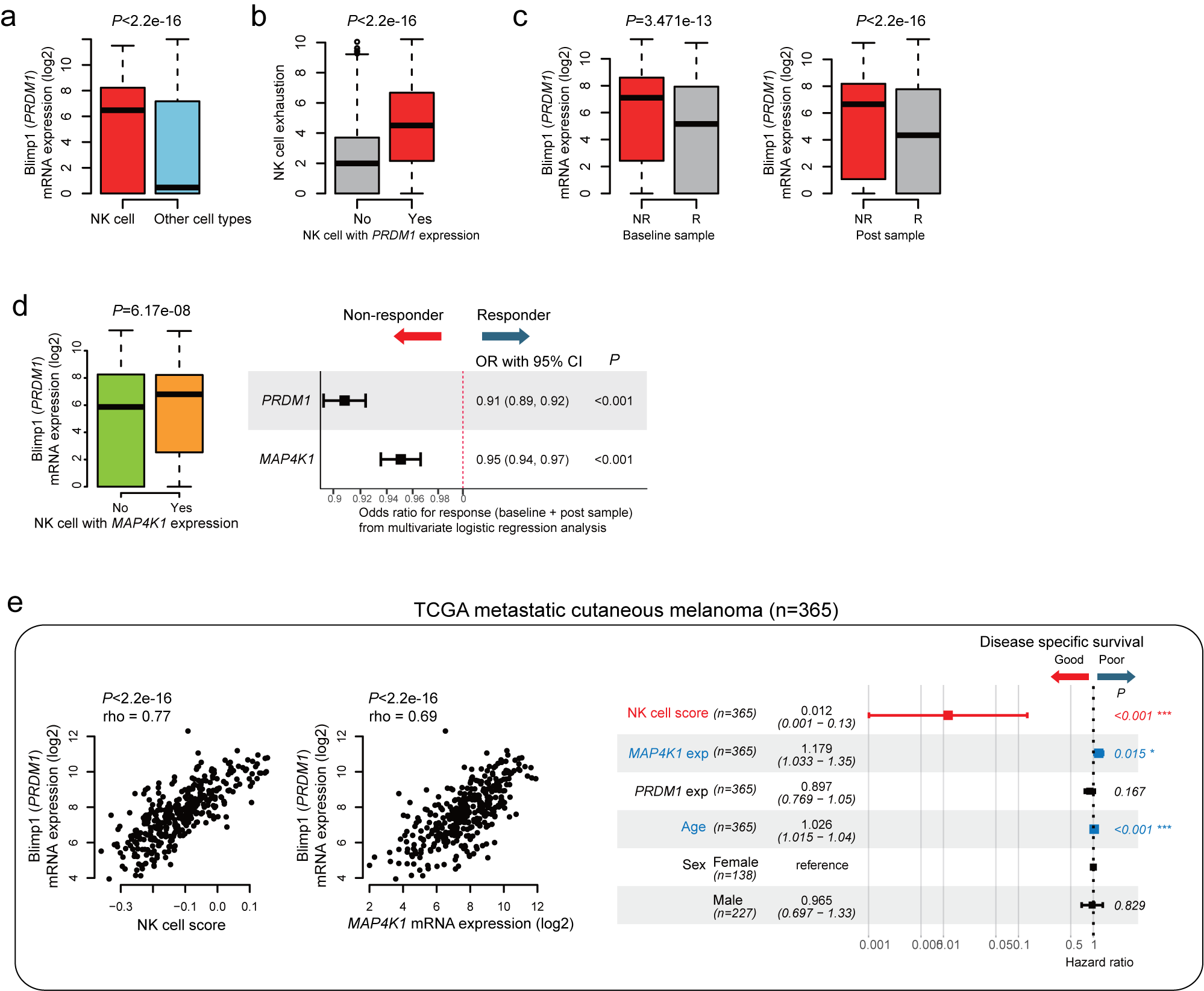


**Figure S20. Blimp1 expression and its association with HPK1 in NK cells.**

(**a**) The expression of Blimp1 (*PRDM1*) in NK cells obtained from melanoma single cell RNA-seq data (GSE120575). The expression of *PRDM1* was high in NK cells, indicating NK cells as an important source of *PRDM1* expression (Wilcoxon rank-sum test).

(**b**) NK cells with high *PRDM1* expression exhibited significantly higher exhaustion score compared to those with low *PRDM1* expression (Wilcoxon rank-sum test).

(**c**) NK cells with high *PRDM1* expression were associated with non-response to checkpoint immunotherapy (Wilcoxon rank-sum test).

(**d**) The high expression of *PRDM1* in NK cells correlated with *MAP4K1* expression. However, they appeared to be independent with respect to non-response to immunotherapy, suggesting a differential role in the immunotherapy resistance (Wilcoxon rank-sum test and multivariate logistic regression analysis).

(**e**) When calculating the NK score in RNA-seq data from TCGA metastatic melanomas (n = 365), the expression of *PRDM1* significantly correlated with NK score (left) and *MAP4K1* expression in NK cells (right) (Spearman correlation test). However, when adjusting for the NK score, the expression of *MAP4K1* but not *PRDM1* was associated with poor prognosis (Multivariate Cox regression analysis). Thus, HPK1 and Blimp1 may have divergent roles in NK cells depending on the context. Actual *P*-values are indicated.

**Table S1**. The demographics and clinicopathological characteristics of melanoma patients.

| **Characteristics** | **Total melanoma**  **(n = 22), n (%)** | **Non-metastatic melanoma**  **(n = 19), n (%)** | **Metastatic melanoma**  **(n = 3), n (%)** | ***P* value** |
| --- | --- | --- | --- | --- |
|  |  |  |  |  |
| **Clinical characteristics** | | | | |
| **Age of diagnosis (years)** | 68.0 (22-84) | 67.0 (22-82) | 72.0 (62-84) | 0.389 |
| **Sex** |  |  |  | >0.999 |
| Male | 11 (50.0) | 10 (52.6) | 1 (33.3) |  |
| Female | 11 (50.0) | 9 (47.4) | 2 (66.7) |  |
| **BMI (kg/m^2^)** | 23.4 (19.3-31.5) | 22.9 (19.3-31.5) | 26.6 (24.1-27.3) | 0.292 |
| **Clinical diameter at the time of diagnosis (mm)** | 19.0 (5.0-56.0) | 15.5 (5.0-40.0) | 35.0 (30.0-56.0) | 0.044 |
| **Location** |  |  |  | 0.312 |
| Head and neck | 5 (22.7) | 3 (15.8) | 2 (66.7) |  |
| Trunk | 2 (9.1) | 2 (10.5) | 0 (0) |  |
| Upper extremities | 3 (13.6) | 3 (15.8) | 0 (0) |  |
| Lower extremities | 12 (54.5) | 11 (57.9) | 1 (33.3) |  |
| **Cancer history before diagnosis of melanoma** | 3/22 (13.6) | 1/19 (5.3) | 2/3 (66.7) | 0.038 |
| **History of sun exposure** | 3/22 (13.6) | 2/19 (10.5) | 1/3 (33.3) | 0.371 |
| **BRAF mutation** | 3/9 (33.3) | 3/6 (50.0) | 0/3 (0) | 0.464 |
| **Amelanotic lesion** | 3/22 (13.6) | 3/19 (15.8) | 0/3 (0) | >0.999 |
| **Laboratory test at the time of research** | | | | |
| **Hb (g/dL)** | 13.3 (11.1-15.9) | 12.9 (11.1-15.9) | 13.9 (13.3-14.1) | 0.615 |
| **WBC (10^3^/µL)** | 6.1 (3.4-9.5) | 6.1 (3.4-7.6) | 6.4 (5.7-9.5) | 0.513 |
| **Neutrophil (%)** | 58.5 (45.1-70.7) | 56.2 (45.1-70.7) | 58.6 (51.4-68.8) | 0.615 |
| **Lymphocyte (%)** | 33.6 (15.7-47.4) | 35.8 (15.7-47.4) | 33.6 (17.2-38.3) | 0.451 |
| **Monocyte (%)** | 7.7 (2.0-9.6) | 7.6 (2.0-9.1) | 7.7 (6.1-9.6) | 0.546 |
| **Pathological characteristics** | | | | |
| **Ulceration** | 2/22 (9.1) | 2/19 (10.5) | 0/3 (0) | >0.999 |
| **Breslow depth (mm)** | 2.5 (0-13) | 0.9 (0-10) | 7.0 (7-13) | 0.012 |
| **Perineural invasion** | 2/20 (10.0) | 1/17 (5.9) | 1/3 (33.3) | 0.284 |
| **Lymphovascular invasion** | 0/20 (0) | 0/17 (0) | 0/3 (0) |  |
| **Melanoma subtype** |  |  |  | 0.286 |
| Superficial spreading | 3 (13.6) | 3 (15.8) | 0 (0) |  |
| Lentigo maligna | 3 (13.6) | 3 (15.8) | 0 (0) |  |
| Acral lentiginous | 11 (50.0) | 10 (52.6) | 1 (33.3) |  |
| Nodular | 5 (22.7) | 3 (15.8) | 2 (66.7) |  |
| **Cancer stage at diagnosis** | | |  |  |
| **AJCC Stage** |  |  |  | 0.002 |
| Stage 0 | 7 (35.0) | 7 (41.2) | 0 (0) |  |
| Stage 1 | 3 (15.0) | 3 (17.6) | 0 (0) |  |
| Stage 2 | 7 (35.0) | 7 (41.2) | 0 (0) |  |
| Stage 3 | 2 (10.0) | 0 (0) | 2 (66.7) |  |
| Stage 4 | 1 (5.0) | 0 (0) | 1 (33.3) |  |
| **Treatment and follow-up data** | | | | |
| **Surgical excision** | 21/22 (95.5) | 18/19 (94.7) | 3/3 (100) | >0.999 |
| **Chemotherapy** | 2/22 (9.1) | 0/19 (0) | 2/3 (66.7) | 0.013 |
| **Radiotherapy** | 1/22 (4.5) | 0/19 (0) | 1/3 (33.3) | 0.136 |
| **Death during follow-up** | 1/22 (4.5) | 0/19 (0) | 1/3 (33.3) | 0.136 |

AJCC, American Joint Committee on Cancer; IQR, interquartile range; SD, standard deviation; BMI, Body mass index

Median values with ranges are presented for all continuous variables
